# Tumor *NLRP3* amplification promotes immunotherapy resistance by inhibiting STAT1 signaling and MHC class I expression

**DOI:** 10.1172/JCI201816

**Published:** 2026-08-13

**Authors:** Balamayroon Theivanthiran, Nagendra Yarla, Kaylee Villarreal, Y-Van Nguyen, Mahere Rezazade Bazaz, Ernesto Pena Calderin, Linda Cao, Kyra Majors, Michael P. Plebanek, Alisha Holtzhausen, Emily Bolch, Douglas B. Johnson, Hope Uronis, John H. Strickler, Nicholas C. DeVito, Brent A. Hanks

**Affiliations:** 1 Lineberger Comprehensive Cancer Center, Department of Medicine, Division of Medical Oncology, Department of Microbiology and Immunology, UNC School of Medicine, University of North Carolina at Chapel Hill, Chapel Hill, North Carolina, USA.; 2 Duke Cancer Institute, Department of Medicine, Division of Medical Oncology, Duke University, Durham, North Carolina, USA.; 3 Department of Pharmacology, UNC School of Medicine, University of North Carolina at Chapel Hill, Chapel Hill, North Carolina, USA.; 4 Department of Medicine, Vanderbilt University Medical Center, Nashville, Tennessee, USA.

**Keywords:** Immunology, Oncology, Cancer immunotherapy, Cellular immune response, MHC class 1

## Abstract

Immunotherapy resistance remains a challenge in immuno-oncology, and predictive biomarkers are needed to guide combination immunotherapy selection for the individual patient. We showed that elevated tumor-intrinsic NOD-, LRR-, and pyrin domain-containing protein 3 (NLRP3) signaling activity correlates with checkpoint inhibitor resistance in several independent cohorts of stage III/IV melanoma and gastroesophageal (GE) adenocarcinoma. In situ hybridization demonstrated tumor *NLRP3* copy number gain in immunotherapy-resistant melanomas and GE adenocarcinomas harboring enhanced NLRP3 signaling activity. *Nlrp3* amplification suppressed NOD-, LRR-, and CARD-containing 5–mediated (NLRC5-mediated) MHC class I upregulation, while spatial transcriptomic analysis of patient-derived GE adenocarcinomas confirmed that NLRP3 signaling activity inversely correlated with NLRC5 and MHC class I–associated gene expression. Mechanistically, NLRP3 binds to and inhibits signal transducer and activator of transcription 1 (STAT1) dimerization, nuclear translocation, and NLRC5 transcription. Consistent with these findings, pharmacologic inhibition of the NLRP3 inflammasome augmented tumor STAT1/NLRC5 signaling, enhanced MHC class I surface expression, and overcame anti–programmed cell death 1 resistance in an orthotopic model of gastric adenocarcinoma. This work reveals a fundamental link between cellular stress and tumor-mediated immune evasion and indicates that the tumor NLRP3 signaling pathway merits further clinical study as a therapeutic target and a source of companion biomarkers for overcoming checkpoint inhibitor resistance in patients with cancer.

## Abstract 2

Tumor stress signaling pathway interferes with immunosurveillance and provides candidate targets and biomarkers for overcoming cancer immunotherapy resistance

## Graphical abstract

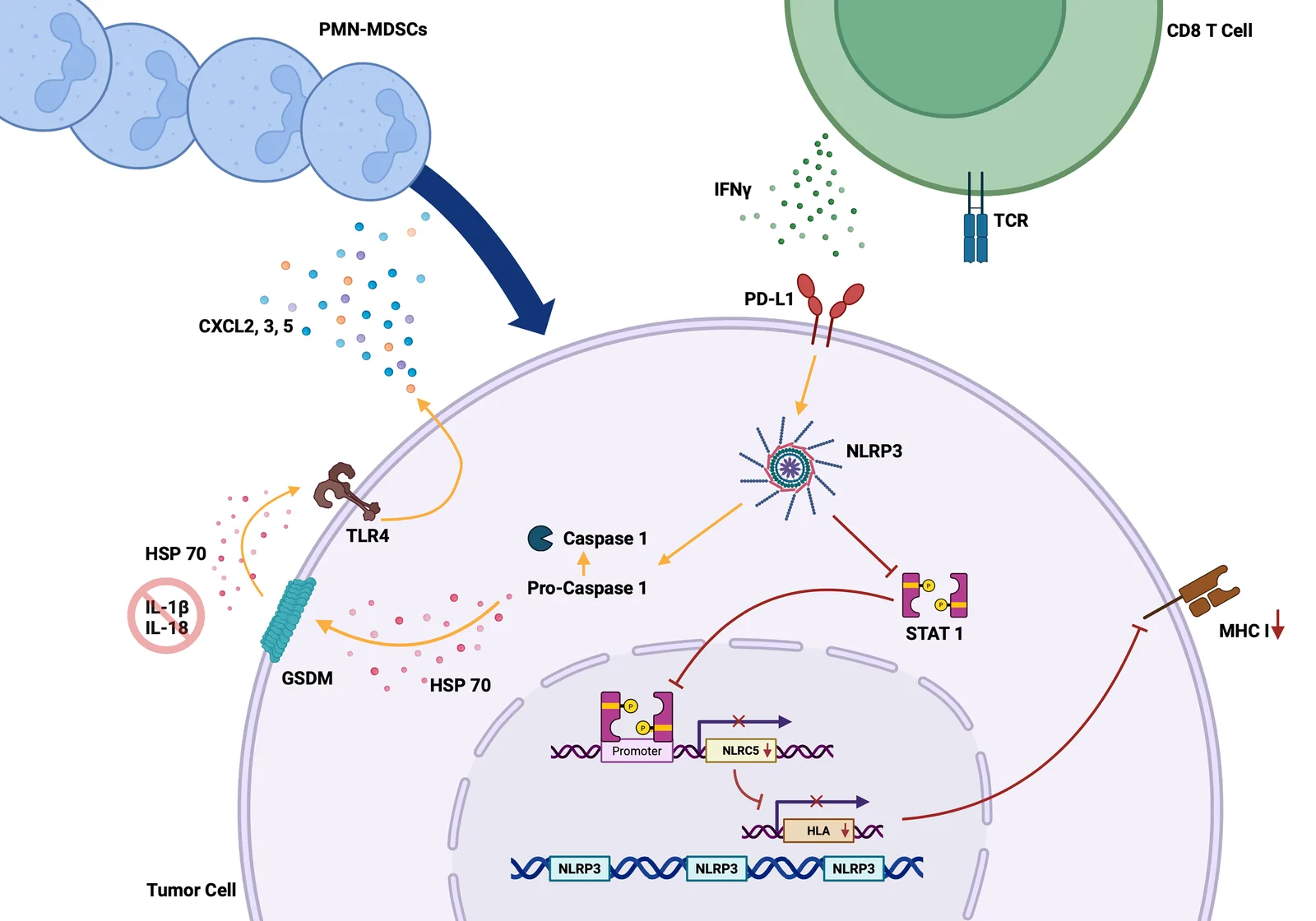

## Introduction

While checkpoint inhibitor immunotherapies targeting programmed cell death 1 (PD-1) have revolutionized clinical oncology, this benefit has been limited to less than 20% of patients with cancer (1). In addition, multiple combination regimens including anti–PD-1 immunotherapy have either failed to improve or only modestly improved outcomes over anti–PD-1 monotherapy for a variety of indications (2, 3). The identification of novel immunotherapeutic targets and companion predictive biomarkers allowing for rational selection of tailored combination regimens for the individual patient is now a priority for advancing the field of immuno-oncology (4).

Using an array of preclinical tumor models, we have previously demonstrated that activation of CD8^+^ T cells in response to PD-1 blockade stimulates a tumor-intrinsic NOD-, LRR-, and pyrin domain-containing protein 3 (NLRP3) inflammasome/heat shock protein 70 (HSP70) signaling axis that generates a CXCR2-dependent chemokine gradient, facilitating the recruitment of granulocytic polymorphonuclear cell–myeloid-derived suppressor cells (PMN-MDSCs) into the tumor bed as well as their accumulation in more distant tissues (5). Both genetic silencing and pharmacologic inhibition of either tumor-expressed NLRP3 or HSP70 enhance CD8^+^ T cell tumor infiltration and augment responses to anti–PD-1 immunotherapy in an autochthonous model of melanoma. We have further shown that elevated baseline levels of activation of the tumor-intrinsic NLRP3 inflammasome correlates with resistance to anti–PD-1 immunotherapy in a cohort of stage IV melanoma (6). These studies also revealed that the cytoplasmic domain of programmed cell death ligand 1 (PD-L1) can trigger tumor NLRP3 inflammasome activation via a STAT3-dsRNA-dependent protein kinase R signaling axis, thus linking tumor PD-L1 expression with NLRP3-dependent adaptive resistance to anti–PD-1 immunotherapy (5). Indeed, genetic silencing of tumor PD-L1 eliminates the activation of the NLRP3/HSP70 pathway, suggesting that the tumor-intrinsic NLRP3/HSP70 signaling pathway may contribute to anti–PD-1 resistance in select PD-L1–positive tumors (5). Interestingly, *NLRP3* copy number gains have been observed in an array of solid tumors (7). However, it is currently unclear if the genetic state of *NLRP3* influences carcinogenesis or determines whether tumor-intrinsic NLRP3 activation occurs in response to anti–PD-1 immunotherapy.

Previous studies have shown that NOD-, LRR-, and CARD-containing 5 (NLRC5) regulates various genes that play a role in MHC class I transcription (8). As such, tumor expression of NLRC5 has been positively correlated with tumor MHC class I expression, tumor-infiltrating CD8^+^ T cell activation levels, and the overall survival of several populations of patients with cancer (9). Functional loss of various genes associated with MHC class I antigen processing and presentation by tumors often serves as a mechanism for evading antitumor immunity, including responses to checkpoint inhibitor immunotherapy (10). Unfortunately, our ability to overcome this evasion strategy to enhance antitumor immunity has been limited (11).

Unlike in melanoma, there have been modest benefits achieved with anti–PD-1 immunotherapy in patients with advanced gastroesophageal (GE) adenocarcinoma, and these responses are rarely durable, leaving patients with few compelling treatment options beyond first-line therapy (12). Even with available combination immunotherapy regimens, the median overall survival of patients with advanced GE adenocarcinoma remains limited to approximately 14 months, and the incidence of this cancer is now increasing, particularly in younger patient populations (13, 14).

Here, we explore the role of *NLRP3* genetic amplification in driving tumor-mediated immune escape during anti–PD-1 immunotherapy in patients with melanoma as well as GE adenocarcinoma and demonstrate tumor-intrinsic NLRP3 activation suppresses NLRC5-mediated transcription of the tumor’s MHC class I antigen presentation machinery. Pharmacological inhibition of NLRP3 is shown to reverse MHC class I downregulation and overcome resistance to anti–PD-1 immunotherapy in autochthonous and orthotopic models of these malignancies, respectively. These studies support the exploration of the tumor-intrinsic NLRP3 inflammasome pathway as a promising source of therapeutic targets and companion biomarkers to overcome anti–PD-1 resistance in difficult-to-treat patient populations.

## Results

### Tumor-intrinsic NLRP3 inflammasome activity and tumor NLRP3 amplification are associated with anti–PD-1 resistance in patients with advanced melanoma.

Using an NLRP3-ASC proximity ligation assay (PLA) as a surrogate marker for NLRP3 inflammasome activity, we have previously shown that elevated baseline tumor-intrinsic NLRP3 signaling activity correlates with resistance to checkpoint inhibitor immunotherapy in previously untreated patients with stage IV melanoma (5). To verify these findings, we examined baseline tumor tissue specimens harvested from an independent cohort of patients with naive stage IV melanoma treated at an outside institution using the same NLRP3-ASC PLA (Figure 1A and Supplemental Table 1; supplemental material available online with this article; https://doi.org/10.1172/JCI201816DS1). This work supported our previous findings, demonstrating that elevated baseline tumor NLRP3 signaling activity correlates with resistance in patients who have received either anti–PD-1 monotherapy or combination anti–PD-1/anti–cytotoxic T lymphocyte antigen 4 (anti–CTLA-4) therapy. Indeed, multivariate analysis of our cumulative patient cohort shows that elevated baseline tumor NLRP3 signaling activity is associated with diminished progression-free survival (PFS) in patients with naive stage IV melanoma undergoing checkpoint inhibitor immunotherapy (HR 0.013, 95% CI: 0.03–0.69, *P* = 0.02) (Figure 1B and Supplemental Tables 1 and 2).

We reasoned that an underlying genetic mechanism was likely determining whether a tumor could leverage the NLRP3 inflammasome pathway to evade antitumor immunity. Prior studies have noted that *NLRP3* genetic amplification occurs in several solid tumors (7). Indeed, approximately 16% of cutaneous melanomas, including a combination of both early- and late-stage disease in The Cancer Genome Atlas (TCGA), harbor amplified *NLRP3* (6, 15). Based on these data, we performed an *NLRP3* fluorescence in situ hybridization (FISH) assay on baseline tumor tissue specimens derived from patients with stage IV melanoma initiating either anti–PD-1 monotherapy or combination anti–PD-1/anti–CTLA-4. This analysis found copy number gains of *NLRP3* in 48% of these patients and an association of this marker with resistance to first-line checkpoint inhibitor immunotherapy (Figure 1C and Supplemental Table 1). Indeed, *NLRP3* gene copy number was found to correlate closely with NLRP3 signaling activity in melanoma tissues, suggesting that copy number gain is the primary genetic mechanism influencing tumor NLRP3 signaling activity (Figure 1D). We further validated these findings by performing *NLRP3* FISH assays on baseline tissue specimens derived from an independent cohort of patients with stage IV melanoma receiving checkpoint inhibitor immunotherapy (Figure 1E). Overall, this work suggests that somatic copy number gains of *NLRP3* by melanoma tissues influence tumor NLRP3 signaling activity and promote resistance to checkpoint inhibitor immunotherapy.

There is currently a substantial need for predictive biomarkers for treating patients with stage III melanoma, as many of these patients derive no clinical benefit from adjuvant checkpoint inhibitor immunotherapy yet are being placed at risk for developing immune-related adverse events. As a result, we conducted NLRP3-ASC PLA and NLRP3 FISH assays on baseline tumor specimens along with an HSP70 enzyme-linked immunosorbent assay (ELISA) on baseline plasma specimens derived from patients with stage III melanoma before initiating adjuvant anti–PD-1 immunotherapy. These studies demonstrated elevated tumor NLRP3 signaling activity, increased baseline plasma levels of HSP70, and tumor *NLRP3* amplification to be associated with anti–PD-1 failure and melanoma recurrence (Figure 1, F–H, and Supplemental Table 3). Again, we found baseline plasma HSP70 levels to correlate closely with tumor NLRP3 activity levels and, in turn, with *NLRP3* amplification levels in this cohort of stage III melanoma (Figure 1, I and J). Consistent with these findings, stage III melanoma patients with tumor NLRP3 activity levels above the median associated with inferior recurrence-free survival relative to those harboring melanomas with NLRP3 activity below the median (*P* = 0.025) (Figure 1K). These cumulative data suggest that tumor-intrinsic NLRP3 signaling activity contributes to immune evasion and checkpoint inhibitor immunotherapy resistance in melanoma (5, 6).

### An NLRP3 transcriptional signature correlates with resistance to anti–PD-1 immunotherapy in patients with advanced melanoma.

To examine the relationship between activation of the tumor-intrinsic NLRP3 inflammasome and immunotherapy resistance in a larger number of patients with melanoma in publicly available clinical datasets, we constructed an unbiased transcriptional signature of NLRP3 activation in melanoma tissues by utilizing a genetically engineered murine model of melanoma (Figure 2A). This included capturing upregulated genes in BRAF^V600E^ PTEN^–/–^ melanomas modified via CRISPRa to exhibit enhanced *Nlrp3* transcriptional activity (BRAF^V600E^ PTEN^–/–^ NLRP3a) and integrating this gene set with the downregulated genes in a newly generated transgenic Tyr-cre BRAF^V600E^ PTEN^fl/fl^ NLRP3^fl/fl^ tumor model exhibiting melanoma-restricted ablation of *Nlrp3* (Figure 2B). Melanoma tissue-restricted knockout of *Nlrp3* expression was first confirmed, and diminished expression of genes known to play a role in PMN recruitment was also verified in BRAF^V600E^ PTEN^–/–^ NLRP3^–/–^ tumors (Supplemental Figure 1, A and B). This was followed by flow cytometry verification of a reduction in PMN-MDSC recruitment by BRAF^V600E^ PTEN^–/–^ NLRP3^–/–^ melanomas versus control BRAF^V600E^ PTEN^–/–^ melanomas (Supplemental Figure 1C). In line with our previous data, we found BRAF^V600E^ PTEN^–/–^ NLRP3^–/–^ tumor growth to be suppressed relative to control BRAF^V600E^ PTEN^–/–^ tumors (Figure 2B). By combining these models, we generated a 48-gene transcriptional profile reflecting tumor-intrinsic NLRP3 activation and found this NLRP3-tsig to positively correlate with a PMN-MDSC transcriptional signature in melanoma tissues using single-sample gene set enrichment analysis (ssGSEA) (Figure 2C and Supplemental Table 4). We then performed receiver-operator characteristic (ROC) curve analyses of NLRP3-tsig applied to independent previously reported datasets of patients with stage IV melanoma treated with anti–PD-1 immunotherapy (16). This work showed the NLRP3 signature to predict resistance to anti–PD-1 immunotherapy with an area under the curve (AUC) value of 0.724 in the Hugo et al. dataset (26 patients) and an AUC value of 0.679 in the Gide et al. dataset (29 patients) (Figure 2D) (16, 17). Indeed, the NLRP3-tsig signature outperformed both the PMN-MDSC signature and a monocytic MDSC (M-MDSC) signature in predicting response to anti–PD-1 immunotherapy in melanoma (Figure 2E) (18). Importantly, the NLRP3-tsig signature also performed favorably compared with previously reported signatures in predicting anti–PD-1 resistance in patients with advanced melanoma, including the ICBresistance, X18signature, TIDE, IMPRES, and IPRES signatures, in the Hugo et al. dataset, as well as the larger Lee et al. dataset (43 patients) (Figure 2, F and G) (16, 19–22). We further found this NLRP3-associated transcriptional signature to be superior in predicting resistance relative to a previously developed NLRP3pancancersig signature constructed from various components of the NLRP3 signaling pathway in both of these datasets (Figure 2, F and G) (23). Finally, our studies indicate that the elevated NLRP3-tsig also correlates with a diminished disease-free survival in TCGA melanoma dataset (Figure 2H). Our cumulative data suggest that the tumor-intrinsic NLRP3 signaling pathway represents a promising source of biomarkers for predicting poor outcomes in patients with advanced melanoma undergoing checkpoint inhibitor immunotherapy.

### Tumor-intrinsic NLRP3 signaling activity suppresses tumor NLRC5 and MHC class I expression levels.

As *NLRP3* amplification correlates with anti–PD-1 immunotherapy resistance in patients with melanoma as well as in the BRAF^V600E^ PTEN^–/–^ melanoma model, we investigated whether tumor-intrinsic NLRP3 signaling triggers additional mechanisms to evade antitumor immunity. We therefore expanded our analysis of the transcriptional alterations induced by *Nlrp3* amplification using the previously engineered murine BRAF^V600E^ PTEN^–/–^ NLRP3a melanoma cell line (6). This work revealed a downregulation in the expression of genes associated with antigen processing and presentation relative to control BRAF^V600E^ PTEN^–/–^ NTC tumors (Figure 3A). While Western blot studies showed IFN-γ stimulation enhanced expression levels of the MHC class I transactivator, NLRC5, in a control BRAF^V600E^ PTEN^–/–^ melanoma cell line (BRAF^V600E^ PTEN^–/–^ NTC), this increase was suppressed in the BRAF^V600E^ PTEN^–/–^ NLRP3a melanoma cell line (Figure 3B). Consistent with these findings, we also found that a small molecule inhibitor of the NLRP3 inflammasome, MCC950, enhanced NLRC5 expression levels in both murine and human melanoma cell lines, as well as a pancreatic ductal adenocarcinoma cell line, indicating this effect also occurs in nonmelanoma tumors (Figure 3C and Supplemental Figure 2, A and B) (24). To determine whether corresponding alterations in MHC class I surface expression are observed in response to NLRP3 signaling activity, we performed pan-MHC class I flow cytometry analysis of BRAF^V600E^ PTEN^–/–^ NLRP3a and BRAF^V600E^ PTEN^–/–^ NTC melanomas following their surgical resection and observed a marked diminishment in MHC class I surface expression in *Nlrp3*-amplified melanomas (Figure 3D). Indeed, pharmacologic inhibition of NLRP3 in BRAF^V600E^ PTEN^–/–^ melanomas in vivo and a pancreatic ductal adenocarcinoma cell line in vitro correspondingly resulted in enhanced levels of MHC class I surface expression (Figure 3D and Supplemental Figure 2C). We have previously demonstrated that the tumor-intrinsic NLRP3 inflammasome pathway triggers the recruitment of PMN-MDSCs into the TME, and others have reported a link between tumor-infiltrating PMN-MDSCs and MHC class I downregulation (25). However, we found no evidence that PMN-MDSC ablation enhances tumor MHC class I expression levels in the transgenic BRAF^V600E^ PTEN^–/–^ melanoma model (Supplemental Figure 2D).

We have previously shown that PD-L1 triggers activation of the NLRP3 inflammasome signaling pathway in tumors (5). As a result, we performed Western blot studies in *Cd274*-silenced BRAF^V600E^ PTEN^–/–^ melanoma cell lines (BRAF^V600E^ PTEN^–/–^ PDL1^KD^) and noted an increase in NLRC5 expression levels, a finding that was phenocopied by an *Nlrp3*-silenced BRAF^V600E^ PTEN^–/–^ melanoma cell line (Supplemental Figure 2E). These findings were consistent with an observed increase in surface MHC class I in response to IFN-γ stimulation in both BRAF^V600E^ PTEN^–/–^ NLRP3^KD^ and BRAF^V600E^ PTEN^–/–^ PDL1^KD^ tumor cell lines (Figure 3E and Supplemental Figure 2F).

In order to examine this pathway more rigorously in vivo, we turned to the autochthonous melanoma model genetically silenced for melanocyte-specific *Nlrp3* expression (Tyr-cre BRAF^V600E^ PTEN^fl/fl^ NLRP3^fl/fl^) described above (Figure 2B). Flow cytometry analysis of the resected melanoma tissues revealed an increase in MHC class I surface expression in BRAF^V600E^ PTEN^–/–^ NLRP3^–/–^ tumors in situ compared with control BRAF^V600E^ PTEN^–/–^ tumors (Figure 3F). Together, this work describes a potentially previously unrecognized role for the NLRP3 inflammasome in suppressing NLRC5 and the MHC class I antigen processing and presentation machinery of tumors.

### Tumor-intrinsic NLRP3 inflammasome inhibits NLRC5 expression by blocking STAT1-mediated transcription.

We initially hypothesized that the tumor-intrinsic NLRP3 pathway may be eliciting the upregulation of a soluble factor that suppresses NLRC5-MHC class I tumor expression, prompting experiments demonstrating that the conditioned medium of *Nlrp3*-amplified BRAF^V600E^ PTEN^–/–^ melanoma cells modestly suppresses IFN-γ–dependent induction of NLRC5 and MHC class I expression (Supplemental Figure 3, A and B). Since soluble HSP70 is released downstream of tumor-intrinsic NLRP3 activation, we also compared NLRC5-MHC class I expression between BRAF^V600E^ PTEN^–/–^ HSP70^–/–^ versus BRAF^V600E^ PTEN^–/–^ NTC control cells and found NLRC5 expression to be moderately elevated in HSP70-ablated tumor cells (Supplemental Figure 3C). Indeed, there was a slight increase in MHC class I surface levels in HSP70-silenced BRAF^V600E^ PTEN^–/–^ melanoma cells, as well as BRAF^V600E^ PTEN^–/–^ melanoma cells treated with an anti-HSP70 antagonistic antibody (Supplemental Figure 3D). However, given the modest overall changes in tumor NLRC5-MHC class I expression observed, we initiated studies to explore other pathways that may be regulated more directly by NLRP3. Therefore, we immunoprecipitated NLRP3 from BRAF^V600E^ PTEN^–/–^ melanoma cells following NLRP3 activation via IFN-γ/anti–PD-L1 ab treatment and analyzed these products by tandem mass spectrometry versus untreated BRAF^V600E^ PTEN^–/–^ melanoma cells. Of the 27 unique proteins found to bind to NLRP3 upon IFN-γ/anti–PD-L1 ab activation in tumor cells, STAT1 was determined to be the most abundant (Figure 4A, Supplemental Figure 4A, and Supplemental Table 5). Since NLRC5 is transcriptionally regulated by STAT1, we conducted a series of co-immunoprecipitation experiments to confirm NLRP3-STAT1 binding in the BRAF^V600E^ PTEN^–/–^ melanoma cell line (Figure 4B). We noted that IFN-γ/anti–PD-L1 ab treatment of BRAF^V600E^ PTEN^–/–^ melanoma cells suppressed phosphorylated STAT1 levels, an effect that was enhanced with NLRP3-FLAG expression (Figure 4C). Indeed, time course studies showed NLRP3-FLAG expression more rapidly suppressed both total and phosphorylated levels of STAT1 in BRAF^V600E^ PTEN^–/–^ melanoma cells (Figure 4D). Based on these findings, we looked for and found no evidence that NLRP3-STAT1 binding promotes STAT1 proteasomal degradation (Supplemental Figure 4B). However, additional Western blot studies following nuclear-cytoplasmic fractionation showed NLRP3-FLAG expression nearly eliminated total STAT1 levels in the nucleus (Figure 4E). As a result, we conducted native non-denaturing Western blots and found NLRP3 suppressed STAT1 dimerization, a process necessary for STAT1 nuclear translocation (Figure 4F) (26). Consistent with these findings, we used a luciferase reporter system to further demonstrate that NLRP3 inhibits STAT1-dependent transcriptional activity (Figure 4G). As STAT1 is known to autoregulate its own transcription, we further confirmed a downregulation of STAT1 and other MHC class I transcript levels in both BRAF^V600E^ PTEN^–/–^ melanoma cells and BRAF^V600E^ PTEN^–/–^ melanoma tissues harboring amplified *Nlrp3* (Figure 4H). Notably, we also determined NLRP3 activation similarly suppressed STAT1 phosphorylation and *Nlrc5* expression in primary bone marrow–derived dendritic cells, suggesting that NLRP3-mediated inhibition of STAT1 and NLRC5 is not restricted to tumors (Supplemental Figure 4, C and D). Finally, we found NLRP3 upregulation to have no impact on tumor cell IFN-γ receptor surface levels or for caspase-1 activity to influence NLRC5 expression (Supplemental Figure 4, E and F).

In summary, this work suggests that the NLRP3 inflammasome complexes with STAT1, thereby inhibiting STAT1 dimerization and nuclear translocation. This process suppresses STAT1-dependent transcription of NLRC5 and appears to be the most dominant mechanism inhibiting downstream expression of MHC class I–associated genes in tumor cells.

### NLRP3 inhibition reverses STAT1-NLRC5-MHC class I suppression and enhances response to anti–PD-1 immunotherapy in an orthotopic model of gastric adenocarcinoma.

A review of TCGA has revealed that several other solid tumor malignancies, including GE adenocarcinoma, also exhibit *NLRP3* amplification (6). As a result, we conducted a series of studies in a murine model of chromosomal instability (CIN) gastric adenocarcinoma derived from a recently engineered Anxa10-CreER^T2^ Kras^G12D/+^ Tp53^R172H/+^ Smad4^fl/fl^ transgenic mouse model (KPS) (Supplemental Figure 5A) (27). Similar to our previous studies, we confirmed IFN-γ/anti–PD-L1 treatment to induce NLRP3 and caspase-1 activation and for this to be associated with enhanced HSP70 release and suppressed NLRC5 expression in the CIN KPS gastric cancer cell line in vitro (Supplemental Figure 5, B–D). After demonstrating that NLRP3 activation also suppresses STAT1 signaling in the CIN KPS gastric cancer model, we confirmed that inhibition of the NLRP3 inflammasome augments nuclear phosphorylated STAT1 levels both in vitro and in CIN KPS gastric tumors in vivo (Figure 5A and Supplemental Figure 6, A–C). In line with these findings, we found NLRP3 inhibition with MCC950 increased levels of NLRC5 and MHC class I expression in vitro (Supplemental Figure 6, D and E). Importantly, we also confirmed increased MHC class I expression by in situ tumor tissues following treatment with an NLRP3 inhibitor (Figure 5, B and C). These observations prompted us to explore whether an NLRP3 inhibitor may overcome resistance to anti–PD-1 immunotherapy in a syngeneic subcutaneous CIN KPS gastric cancer model. This study demonstrated NLRP3 inhibitor therapy alone and in combination with anti–PD-1 to suppress tumor progression more effectively than anti–PD-1 monotherapy (Supplemental Figure 6F). Flow cytometry analysis of these resected tumors showed this effect correlated with diminished PMN-MDSC recruitment and enhanced levels of CD8^+^ T cell infiltration (Supplemental Figure 6G). To evaluate the impact of an NLRP3 inhibitor in a more rigorous model of GE adenocarcinoma, we evaluated NLRP3 inhibitor/anti–PD-1 combination therapy in murine hosts harboring orthotopic CIN KPS gastric cancers (Figure 5D). These tumors were allowed to progress for 8 days followed by treatment on 3 arms: (a) anti–PD-1 immunotherapy every 3 days alone, (b) NLRP3 inhibitor dosed every other day alone, and (c) combination treatment (Figure 5E). This study demonstrated NLRP3 inhibitor/anti–PD-1 immunotherapy enhanced the survival of mice harboring orthotopic KPS gastric tumors versus those mice receiving either agent alone (Figure 5F). Indeed, NLRP3 inhibitor/anti–PD-1 combination therapy suppressed KPS gastric tumor progression based on gastric tumor weight measurements (Supplemental Figure 6H). In order to continuously monitor these orthotopic tumors during the treatment, we utilized an In Vivo Imaging System (IVIS) platform to track tandem-dimer Tomato–expressing (tdTomato-expressing) CIN KPS gastric tumors implanted into the gastric lining of syngeneic mice (Figure 5D). Consistent with our prior data, the addition of an NLRP3 inhibitor suppressed primary CIN gastric tumor growth while anti–PD-1 immunotherapy alone had only a modest suppressive effect (Figure 5, G and H). This study further showed anti–PD-1 immunotherapy enhanced tumor-intrinsic NLRP3 signaling activity and HSP70 release into the plasma, effects that were reversed with the NLRP3 inhibitor, MCC950 (Figure 5I) (5, 6). Based on both flow cytometry and IHC, we further found NLRP3 inhibition to suppress PMN-MDSC accumulation while enhancing CD8^+^ T cell infiltration into these orthotopic CIN gastric tumors (Figure 5J and Supplemental Figure 6, I and J). Also consistent with these findings, an *Nlrp3*-amplified CIN KPS gastric cancer cell line was engineered and verified to exhibit diminished STAT1 phosphorylation and MHC class I expression in response to IFN-γ (Supplemental Figure 6K). Indeed, additional orthotopic tumor studies demonstrated *Nlrp3*-amplified CIN KPS gastric tumors progressed more rapidly than control CIN KPS gastric tumors and to be associated with inferior survival, lower MHC class I surface expression, enhanced levels of PMN-MDSCs, and suppressed CD8^+^ T cell tumor infiltration (Supplemental Figure 6, L–O).

Cumulatively, these data indicate that the tumor-intrinsic NLRP3 inflammasome contributes to immune evasion via multiple mechanisms and that blocking this pathway may facilitate anti–PD-1 responses in immunotherapy-refractory tumors, such as GE adenocarcinoma. Based on these findings, we sought to examine the role of the tumor-intrinsic NLRP3 pathway in chemoimmunotherapy resistance in patients with GE adenocarcinoma.

### Tumor-intrinsic NLRP3 inflammasome activity and tumor NLRP3 amplification is associated with chemoimmunotherapy resistance in patients with advanced GE cancer.

An opportunity to further explore the role of the tumor-intrinsic NLRP3 signaling pathway in immune evasion in GE adenocarcinoma patients was provided by the phase II KeyLARGO clinical trial, which evaluated first-line capecitabine/oxaliplatin chemotherapy with pembrolizumab (anti–PD-1 antibody) in unresectable stage III or stage IV microsatellite-stable, human epidermal growth factor receptor 2–negative GE adenocarcinoma patients following an 8-day lead-in of pembrolizumab monotherapy (28). In this study, both tissue and blood specimens were acquired at baseline and at day 8 prior to initiating chemotherapy, providing a window of opportunity to analyze the impact of anti–PD-1 immunotherapy alone on GE adenocarcinoma tissues (Figure 6, A and B, and Supplemental Table 6). Notably, prior studies observed no relationship between PD-L1 Combined Positive Score testing in these tumor tissue specimens and treatment response in this patient cohort (Supplemental Figure 7A) (28). Paired tissue specimens were harvested from 10 patients, evaluated by a board-certified gastrointestinal pathologist, and analyzed utilizing IF microscopy. We found an increased number of tumor-infiltrating CD8^+^ T cells at day 8 relative to baseline following treatment with pembrolizumab (Figure 6C). This analysis also demonstrated an increased number of baseline tumor-infiltrating CD8^+^ T cells in responders versus nonresponders (Figure 6D). Furthermore, this increase in tumor-infiltrating CD8^+^ T cells following pembrolizumab immunotherapy was noted to occur only in responding patients (Figure 6E). Using elastase as a marker of neutrophils or PMNs, an increase in this cell population was also noted to occur by day 8 following pembrolizumab immunotherapy (Figure 6F). Baseline PMNs were elevated in nonresponders relative to responding patients, and an increase in PMNs with pembrolizumab immunotherapy was noted to occur only in nonresponding patients (Figure 6, G and H). Assuming that these PMNs are functionally suppressive, and therefore consistent with PMN-MDSCs, these data are compatible with our prior work in melanoma showing an increase in tumor-infiltrating PMN-MDSCs upon treatment with anti–PD-1 antibody in resistant tumors (5). As we previously showed that this effect was secondary to activation of the tumor-intrinsic NLRP3 inflammasome, we assessed these tissue specimens using the NLRP3-ASC PLA and found the tumors of nonresponders to exhibit elevated NLRP3 signaling activity (Figure 6I). In light of this relationship, we then evaluated the accompanying plasma samples from an expanded patient cohort derived from this study and found elevated baseline HSP70 plasma levels to correlate with progressive disease (Figure 6J). Similar to our previous findings related to PMNs, we also found only nonresponding patients to demonstrate an increase in plasma HSP70 upon treatment with pembrolizumab immunotherapy (Figure 6K). Notably, baseline levels of CD8^+^ T cells and PMNs, as well as plasma HSP70, correlated with tumor NLRP3 signaling activity based on the NLRP3-ASC PLA (Supplemental Figure 7B). Given our prior data implicating *NLRP3* amplification as an important factor determining response to checkpoint inhibitor immunotherapy in melanoma (Figure 1, C–E), we also performed *NLRP3* FISH analysis of these GE adenocarcinoma tissue specimens. Consistent with our previous studies, this work determined that GE adenocarcinomas exhibiting baseline *NLRP3* amplification failed to respond to this chemoimmunotherapy regimen (Figure 6L). Indeed, patients harboring GE adenocarcinomas exhibiting elevated levels of *NLRP3* amplification were found to associate with an inferior PFS (*P* = 0.0087) and overall survival (*P* = 0.0091) relative to those patients with nonamplified tumors (Figure 6M). We then utilized the NLRP3-tsig described above and found it to also correlate with the PMN-MDSC signature and with disease-free survival in the gastric adenocarcinoma TCGA dataset (Figure 6, N and O). Although these data are derived from a limited patient cohort, the results are consistent with our preclinical studies, suggesting that tumor *NLRP3* amplification and enhanced NLRP3 signaling correlate with anti–PD-1 resistance in GE adenocarcinoma.

### Tumor-intrinsic NLRP3 expression levels inversely correlate with NLRC5 and MHC class I expression levels in patients with advanced GE cancer.

Applying spatial transcriptomics to day 0 and day 8 paired tumor specimens derived from the GE adenocarcinoma patient cohort undergoing anti–PD-1 immunotherapy, we found *NLRP3* expression levels to be elevated in nonresponding relative to responding patients (Figure 7A). We further found increased expression of genes associated with the NLRP3 inflammasome complex in baseline tumor tissue specimens based on KEGG pathway analysis in nonresponding patients while these tumor tissues also demonstrated increased NLRP3 inflammasome expression levels by day 8 of anti–PD-1 therapy relative to day 0 (Figure 7B). Other transcripts associated with the tumor-intrinsic NLRP3 signaling pathway, such as *CXCL5*, were also found to be upregulated in nonresponding patients (Supplemental Figure 8A) (5). This transcriptional analysis further revealed several genes associated with MHC class I antigen processing and presentation to be downregulated by day 8 of anti–PD-1 therapy, including *B2M* (β2-microglobulin), *TAP1/2* (transporter-associated with antigen processing-1/2), and several *HLA* (human leukocyte antigen) alleles (Figure 7C). Interestingly, a decrease in *HLA-A* and *HLA-B* expression by day 8 of anti–PD-1 immunotherapy was also found to be restricted to the tumor tissues harvested from nonresponders, while this was not observed in the tumor tissues of responders (Figure 7D). Indeed, these observations were concordant with pan-MHC class I IHC studies, where increased expression levels of MHC class I were noted exclusively in the tumor tissues of responders (Figure 7D). In light of our previous data, we also examined the relationship between NLRP3 activation and NLRC5 expression levels in these tissues. These studies confirmed NLRC5 expression levels were also diminished in day 8 relative to day 0 tumor tissues while IHC showed elevated NLRC5 expression levels in responding relative to nonresponding patients with GE adenocarcinoma (Figure 7, E and F). Interestingly, transcripts associated with cellular stress-related pathways were also found to be increased, while transcripts associated with antigen processing and presentation were diminished in the same nonresponding patients following anti–PD-1 immunotherapy (Figure 7G) (29).

Consistent with our prior work, we also found tumor tissues with elevated *NLRP3* expression to exhibit lower *NLRC5* transcriptional levels (Figure 7H). While *NLRP3* expression levels uniformly increased in nonresponders in day 8 tumor tissues, *NLRC5* expression levels decreased in these same tumors by day 8 (Figure 7H). Further analysis of individual tumor regions with increased CD8^+^ T cells and PMNs correlated with enhanced expression of the NLRP3 inflammasome and diminished MHC class I–associated gene expression (Supplemental Figure 8B). Indeed, we found individual MHC class I–associated transcripts to be elevated in CD8^+^ T cell–rich regions and diminished in PMN-rich regions (Supplemental Figure 8C). This reciprocal relationship was true for select myeloid markers that were elevated in PMN-rich regions and downregulated in CD8^+^ T cell–rich regions (Supplemental Figure 8C). To confirm that this relationship between NLRP3 and NLRC5/MHC class I also existed in other tumors beyond GE adenocarcinoma, we turned again to tumor tissue specimens harvested from patients with melanoma and performed the NLRP3-ASC PLA described above along with NLRC5 and pan-MHC class I IHC. This study verified that tumor-intrinsic NLRP3 inflammasome activity inversely correlates with both NLRC5 and MHC class I expression in patient-derived melanoma tissues, consistent with our findings in the transgenic BRAF^V600E^ PTEN^–/–^ melanoma model (Figure 7I). Overall, our studies in patient-derived GE adenocarcinoma tissues support our preclinical mechanistic data and further indicate that the activation of the tumor-intrinsic NLRP3 pathway suppresses MHC class I expression and correlates with inferior response to treatment.

## Discussion

Using an array of preclinical tumor models, we have previously reported that the tumor-intrinsic NLRP3 inflammasome triggers the recruitment of PMN-MDSCs into the TME in response to the induction of CD8^+^ T cell activation (5, 6). We now report that activation of the tumor-intrinsic NLRP3 inflammasome also suppresses NLRC5-mediated transcriptional activation of MHC class I–associated genes, thus impeding CD8^+^ T cell immunosurveillance. Mechanistic studies in a transgenic melanoma model and an orthotopic gastric adenocarcinoma model demonstrate that upon oligomerization, NLRP3 binds to STAT1, inhibiting its dimerization, nuclear translocation, and transcriptional activation of NLRC5 (30). This inverse relationship between tumor-intrinsic NLRP3 signaling and NLRC5 expression was also confirmed in patient-derived melanoma and GE adenocarcinoma tissue specimens. The current study further suggests that tumor NLRP3 signaling activity, as well as the *NLRP3* genetic amplification level, correlates with resistance to anti–PD-1 immunotherapy based on tumor and plasma specimens derived from 2 independent cohorts of stage IV melanoma (62 patients) as well as smaller cohorts of stage III melanoma and advanced GE adenocarcinoma (13 and 35 patients, respectively). Along with a generated transcriptional signature of tumor-intrinsic NLRP3 signaling, these findings are also supported by an analysis of 3 independent datasets of advanced melanoma treated with anti–PD-1 immunotherapy (98 patients). Given these cumulative data, we propose that tumor-intrinsic NLRP3 serves as a stress-responsive modulator of adaptive resistance that may ultimately contribute to either primary or secondary immunotherapy resistance depending upon its activation kinetics.

There has been an extensive body of literature describing the activation mechanisms of the NLRP3 inflammasome in myeloid cell populations, and investigators have described 2 distinct steps involved in this process, including a priming phase that serves to enhance NLRP3 expression levels and an activation phase composed of a trigger to induce the formation of the multimolecular NLRP3 inflammasome complex (31). We propose that *NLRP3* copy number gains functionally serve as the priming phase in malignant cells, whereby those tumor cells harboring copy number gains of *NLRP3* are more likely to stimulate the NLRP3 signaling pathway in response to the activation of tumor-infiltrating CD8^+^ T cells. While stimulation of the tumor-intrinsic NLRP3 inflammasome is augmented following anti–PD-1–mediated activation of CD8^+^ T cells, we have also observed basal induction of the NLRP3 pathway in *NLRP3*-amplified tumors. Interestingly, a prior study evaluating somatic mutations and copy number alterations in a series of solid tumors found *NLRP3* copy number gains to be particularly common (7). This is further consistent with previous work demonstrating that the 1q44 chromosomal region harboring *NLRP3* is frequently gained and associated with an inferior prognosis in several solid tumors (32, 33). While it would seem that *NLRP3* amplification may place tumors at risk for enhanced pyroptotic death, we do not observe this effect and are investigating whether certain tumor-intrinsic alterations are uncoupling pyroptosis from NLRP3 activation. Overall, these findings suggest that additional studies are needed to verify the use of *NLRP3* FISH assays to predict immunotherapy outcomes in patients with cancer.

Importantly, our prior work has indicated that the activation of this pathway is dependent upon tumor PD-L1 expression (5). Indeed, genetic silencing of tumor PD-L1 expression inhibits NLRP3 inflammasome activation and downstream expression of the necessary CXCR2 chemokine gradient required for PMN-MDSC recruitment. The impact of checkpoint inhibitor immunotherapy in oncology has remained limited in many tumor types despite the execution of an array of clinical trials testing combination anti–PD-(L)1 regimens (34). One of the primary factors contributing to this barrier in immuno-oncology is the lack of a clinically practical predictive biomarker capable of reliably identifying those tumors that are unlikely to respond to anti–PD-(L)1 regimens (2). While PD-L1 IHC-based scoring has been shown to enrich for those tumors more likely to respond to anti–PD-(L)1 immunotherapy, PD-L1 IHC testing often fails to correlate with clinical outcomes (35). This has contributed to controversies regarding PD-L1 scoring thresholds for use of anti–PD-(L)1 immunotherapy in certain tumor types, such as in GE adenocarcinoma (36, 37). Based on our findings, we propose that the tumor-intrinsic NLRP3 inflammasome pathway contributes to checkpoint inhibitor resistance in PD-L1–positive tumors and that tumor-restricted NLRP3 activation markers or the *NLRP3* FISH assay may serve to improve our ability to predict clinical outcomes for candidates of checkpoint inhibitor immunotherapies.

We developed an unbiased transcriptional signature of tumor-intrinsic NLRP3 inflammasome signaling activity in order to examine the impact of this pathway on clinical response to anti–PD-1 immunotherapy in additional cohorts of patients with melanoma. In addition to extending our analysis to larger patient numbers, this approach was taken to allow for a direct comparison with previously published transcriptional signatures developed to predict response to checkpoint inhibitor immunotherapy (16, 17). Prior studies have developed NLRP3 inflammasome signatures utilized to study the impact of this pathway on tumor biology (23, 38). However, the approach used to construct these signatures has been problematic in that they often will utilize selected components of the known NLRP3 inflammasome signaling pathway and do not consider feedback inhibition of NLRP3 signaling activity on the expression of genes involved in the pathway. In addition, NLRP3 may have functions that do not involve components of the canonical inflammasome pathway. To overcome these deficiencies, we engineered and transcriptionally profiled an autochthonous *Nlrp3*-silenced BRAF^V600E^ PTEN^–/–^ melanoma model (BRAF^V600E^ PTEN^–/–^ NLRP3^–/–^) as well as an *Nlrp3*-amplified BRAF^V600E^ PTEN^–/–^ melanoma cell line (BRAF^V600E^ PTEN^–/–^ NLRP3a) in situ. Those genes upregulated in syngeneic BRAF^V600E^ PTEN^–/–^ NLRP3a tumors were integrated with genes found to be downregulated in autochthonous BRAF^V600E^ PTEN^–/–^ NLRP3^–/–^ tumors. The resulting 48-gene transcriptional signature for tumor-intrinsic NLRP3 signaling activity (NLRP3-tsig) correlates closely with a previously reported transcriptional signature for PMN-MDSCs in both human melanomas and gastric adenocarcinomas yet is superior to the PMN-MDSC signature in predicting resistance to anti–PD-1 immunotherapy in patients with advanced melanoma. Additional studies demonstrate that the NLRP3 activity signature performed favorably versus other published transcriptional signatures in predicting resistance to anti–PD-1 immunotherapy in 2 additional cohorts of patients with stage IV melanoma (16, 17, 22). These results further support the importance of tumor-intrinsic NLRP3 signaling in mediating resistance to anti–PD-1 immunotherapy.

MHC class I downregulation is a well-known mechanism of tumor-mediated immune evasion and the development of anti–PD-1 resistance. Studies have also suggested that inducing tumor MHC class I expression can augment responses to anti–PD-1 immunotherapy (39). Once a tumor loses the capacity for MHC class I antigen processing and presentation, there are few pathways that can be leveraged to reverse this effect, and there are no therapeutics available to overcome this barrier in the clinic. Herein, we show that systemic pharmacologic inhibition of NLRP3 enhances NLRC5-mediated upregulation of MHC class I expression in tumors in vivo, suggesting that NLRP3 inhibition promises to augment other immunotherapy strategies. It is also possible that NLRP3-mediated inhibition of MHC class I antigen processing and presentation may contribute to tumorigenesis by interfering with immunosurveillance, serving as a potential mechanistic link between inflammation and carcinogenesis. Indeed, *H*. *pylori* has been associated with an estimated 70%–90% of gastric cancer cases worldwide, and the *H*. *pylori*-expressed cytotoxin-related gene A protein has been shown to promote gastric cancer cell invasion via the activation of NLRP3 (40, 41).

Recent studies have demonstrated that chronic IFN-γ stimulation serves to suppress antitumor immunity (42). We have found that IFN-γ is necessary to stimulate the tumor-intrinsic NLRP3 signaling pathway, raising the possibility that the induction of tumor NLRP3 activation may contribute to the immunosuppressive effects of chronic IFN-γ exposure.

This work suggests that tumor NLRP3 inflammasome activity correlates with enhanced levels of PMN-MDSCs within the TME and resistance to immunotherapy in patients with GE adenocarcinoma. These findings were further supported using both genetically engineered syngeneic and orthotopic models of gastric adenocarcinoma, showing that pharmacologic inhibition of NLRP3 suppresses PMN-MDSC recruitment, enhances tumor MHC class I expression, and suppresses tumor progression. Recent clinical studies have demonstrated limited benefit of CXCR2 inhibitors in combination with pembrolizumab in tumor types that typically respond poorly to immunotherapy (43). Based on our data, we would conjecture that targeting tumors harboring copy number gains in *NLRP3* are more likely to benefit from this treatment approach. We would further propose that targeting the NLRP3 inflammasome pathway is likely to be more effective than direct CXCR2 inhibition given its ability to block tumor MHC class I antigen presentation based on the studies presented here.

The treatment arsenal available for patients with GE adenocarcinoma is extremely limited, and the prognosis of patients with advanced GE adenocarcinoma remains poor. We acknowledge that the retrospective nature of the clinical studies cited in this study is based on limited patient cohorts, and they are only hypothesis generating. However, in light of our cumulative data, we propose that clinical trial testing of NLRP3 inhibitors in combination with anti–PD-1 immunotherapy is indicated. Such a study would further provide a platform for verifying our hypotheses that *NLRP3*-amplified GE adenocarcinomas are resistant to anti–PD-1 immunotherapy regimens regardless of their level of PD-L1 expression and that targeting NLRP3 is more likely to be effective as a treatment strategy for *NLRP3*-amplified tumors.

## Methods

### Sex as a biological variable.

All animal studies included an equal number of both male and female mice in each experimental group, and similar results were observed in both sexes.

### Statistics.

Unpaired *t* tests were used to compare mean differences between control and treatment groups. One- or 2-way ANOVAs followed by Tukey’s multiple comparisons test or Šídák’s multiple comparisons test, respectively, were performed to analyze data containing 3 or more groups. Various parameters were correlated based on Pearson’s correlation coefficient analysis. Multivariate analysis performed for PFS and overall survival in naive stage IV melanoma patients undergoing checkpoint inhibitor immunotherapy was based on a Cox proportional hazards model. Recurrence-free survival in stage III melanoma patients undergoing anti–PD-1 immunotherapy and PFS/overall survival in GE adenocarcinoma patients undergoing chemoimmunotherapy were analyzed using a log-rank test. All tests were 2-sided with *P* values reported. A *P* value of less than 0.05 was considered significant. All quantitative data are presented as the mean ± SEM. Specific statistical tests are reported in the figure legends. GraphPad Prism 10 was used for all statistical analyses.

### Study approval.

All animal procedures followed protocols approved by the IACUC at either Duke University Medical Center (A089-24-04) or the University of North Carolina at Chapel Hill (25-076.0). All clinical specimens were acquired following informed consent on IRB-approved clinical protocols conducted at Duke University Medical Center (NCT02694965, NCT03342937, NCT03342937) and Vanderbilt University Medical Center (Institutional Protocol Number: 100178).

### Data availability.

All RNA-seq and Nanostring GeoMx spatial transcriptomic data have been deposited in the Gene Expression Omnibus under accession numbers GSE331449 and GSE334591, respectively. All tandem mass spectrometric data have been deposited in MassIVE under accession number MSV000101820. Requests for research materials generated in this study should be addressed to the corresponding author and will be shared upon completion of a Materials Transfer Agreement. Values for all data points are reported in the Supporting Data Values file.

Additional methods are available in Supplemental Methods.

## Author contributions

BT and BAH conceptualized and managed the project. BT or NY performed or directed all experiments. KV, YN, LC, MRB, and EPC supported Western blot, qRT-PCR, cell line generation, and mouse studies. NCD and KM supported the bioinformatic studies. BAH, DBJ, EB, HU, and JHS provided clinical resources for the project. BAH wrote the manuscript. BAH, BT, NCD, EPC, MPP, and AH reviewed and edited the manuscript.

## Conflict of interest

BAH receives research funding from Merck & Co. BAH is a consultant for Regeneron, Amgen, Astellas, and Compugen. BAH and BT are inventors on patent application DU7650PROV submitted by Duke University that covers the tumor-intrinsic NLRP3 inflammasome signaling pathway as a genetic and functional biomarker for immunotherapy response.

## Funding support

This work is the result of NIH funding, in whole or in part, and is subject to the NIH Public Access Policy. Through acceptance of this federal funding, the NIH has been given a right to make the work publicly available in PubMed Central.

- NIH R37CA249085 (to BAH).
- AACR-Debbie’s Dream Foundation Innovation and Discovery Grant (to BAH).
- Department of Defense Mid-Career Award in Melanoma (HT9425-24-1-0282 to BAH).
- Ross Bierkan Melanoma Research Fund (to BAH).
- Duke University Health Scholar Award (to BAH).
- Duke Strong Start Award (to BAH).
- Merck & Co. Pre-Clinical Award (to BAH).
- NIH National Cancer Institute Center Core Support Grant (P30CA016086) (to UNC Pathology Services Core, PSC).

## Supplementary Material

Supplemental data

Unedited blot and gel images

Supporting data values

## Figures and Tables

**Figure 1:**
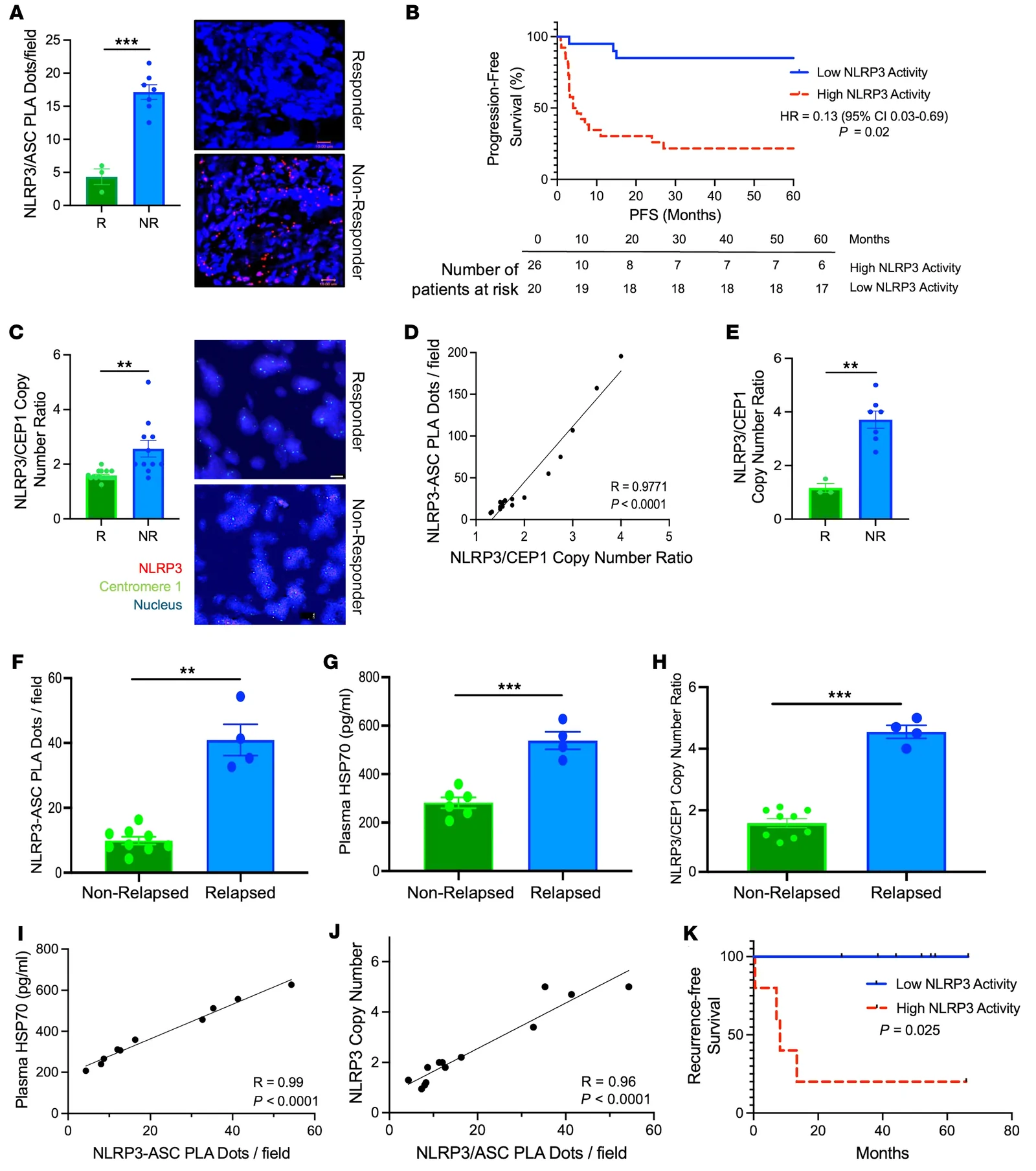
Elevated tumor-intrinsic NLRP3 activity and *NLRP3* amplification correlate with anti–PD-1 resistance in multiple cohorts of patients with advanced melanoma. (**A**) Tumor NLRP3 inflammasome activity levels measured based on NLRP3-ASC proximity ligation assays (PLAs) in responder (R) and nonresponder (NR) patients with stage IV melanoma treated with anti–PD-1 monotherapy. Quantified as dots per high-power field (hpf). Original magnification, 40×. Right, representative PLA images in R and NR patients. Scale bar = 10 μm. Vanderbilt patient cohort. (**B**) Progression-free survival (PFS) of patients with naive stage IV melanoma treated with anti–PD-1 monotherapy stratified based on baseline NLRP3-ASC PLA scores below and above the median. Multivariate analysis performed using the Cox proportional hazards model. (**C**) Baseline tumor NLRP3/CEP1 copy number ratios based on FISH in R and NR patients with stage IV melanoma undergoing anti–PD-1 monotherapy. Right, representative FISH images in R and NR patients. Original magnification, 100×. Scale bar = 5 μm. Duke patient cohort. CEP1, chromosome enumeration probe 1. (**D**) Spearman’s correlation analysis between NLRP3 activity levels based on NLRP3-ASC PLAs and *NLRP3* copy number based on FISH in stage IV melanoma tissues. (**E**) Tumor NLRP3/CEP1 copy number ratios based on FISH in R and NR patients with stage IV melanoma. Vanderbilt patient cohort. (**F**) Baseline tumor NLRP3 inflammasome activity levels measured based on NLRP3-ASC PLAs in nonrelapsed and relapsed patients with stage III melanoma treated with adjuvant anti–PD-1 immunotherapy. (**G**) Plasma HSP70 levels based on ELISA in nonrelapsed and relapsed patients with stage III melanoma treated with adjuvant anti–PD-1 immunotherapy. (**H**) Tumor NLRP3/CEP1 copy number ratios in nonrelapsed and relapsed patients with stage III melanoma treated with adjuvant anti–PD-1 immunotherapy. (**I** and **J**) Spearman’s correlation analysis between NLRP3 activity levels based on NLRP3-ASC PLAs and HSP70 plasma levels based on ELISA (**I**) and NLRP3-ASC PLAs and *NLRP3* copy number based on FISH (**J**) in stage III melanoma tissues. (**K**) Recurrence-free survival (RFS) of patients with stage III melanoma treated with adjuvant anti–PD-1 immunotherapy stratified based on baseline NLRP3-ASC PLA scores below and above the median. Statistical analysis performed by log-rank test. **A**, **C**, and **E**–**H**: statistical analysis performed using unpaired *t* tests. Data expressed as mean values ± SEM. **P < 0.005, ***P < 0.0005.

**Figure 2:**
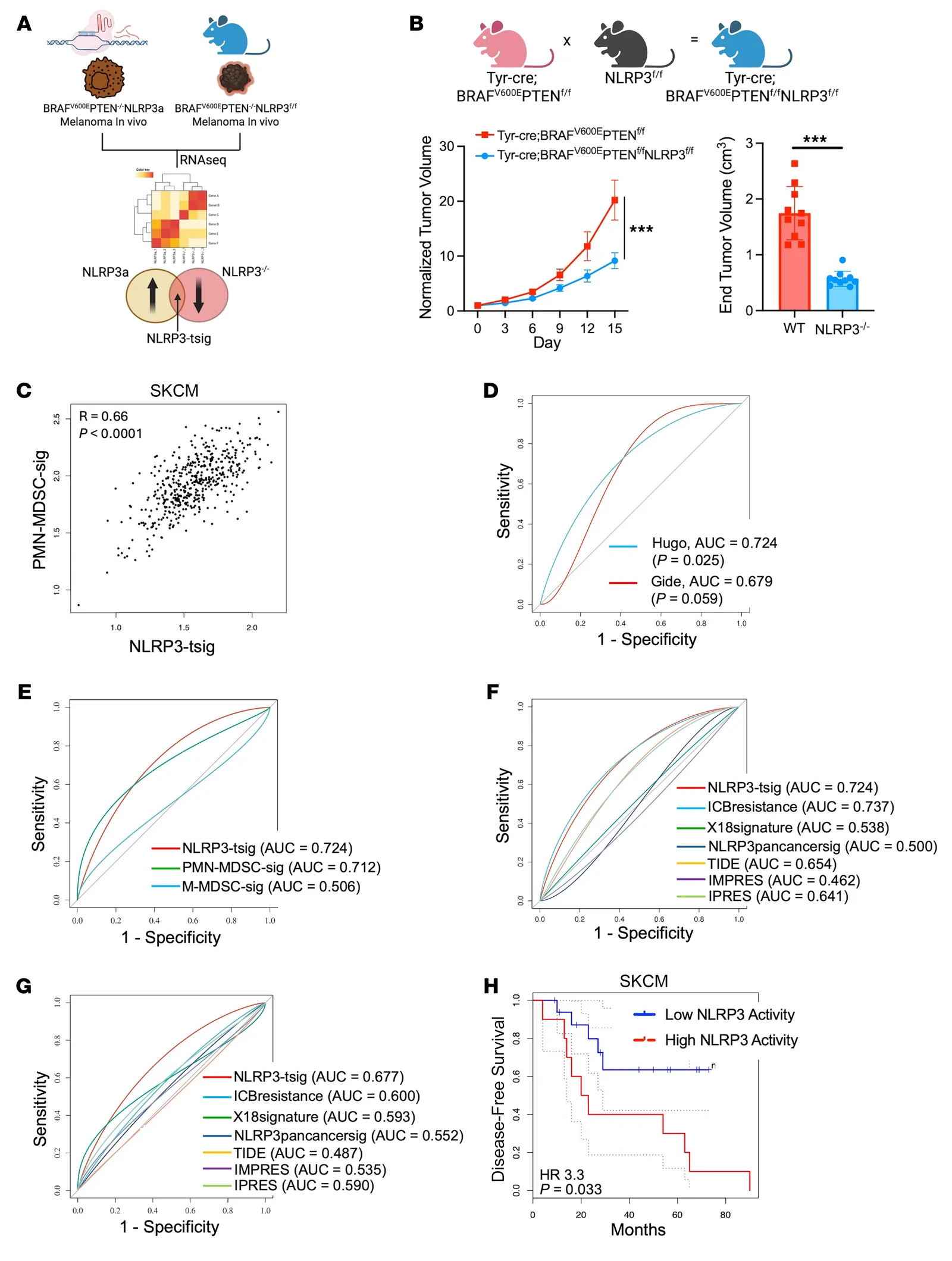
Tumor NLRP3 inflammasome transcriptional signature predicts for anti–PD-1 resistance in multiple cohorts of patients with stage IV melanoma. (**A**) Schematic describing the generation of a tumor NLRP3 transcriptional signature (NLRP3-tsig). BRAF^V600E^ PTEN^–/–^NLRP3a cell line was generated using a CRISPR activation (CRISPRa) protocol and implanted into syngeneic mice by subcutaneous injection. The Tyr-cre BRAF^V600E^ PTEN^–/–^ NLRP3^fl/fl^ tumor model is a transgenic melanoma model with melanoma tissue–restricted silencing of *Nlrp3*. Each of these tumor models were resected for RNA-seq. Generated using BioRender. NLRP3a, *Nlrp3* amplification. (**B**) Top, schematic illustrating the generation of the melanoma-specific *Nlrp3*^–/–^ transgenic mouse model: Tyr-cre BRAF^V600E^ PTEN^fl/fl^ NLRP3^fl/fl^ (NLRP3^–/–^). Generated using BioRender. Bottom left, induced primary melanoma progression in the NLRP3^–/–^ and ctrl transgenic melanoma mouse strains. Bottom right, end tumor volumes in the NLRP3^–/–^ and ctrl transgenic melanoma models. Statistical analysis performed using unpaired *t* tests. (**C**) Single-sample gene set enrichment analysis (ssGSEA) shows a PMN-MDSC transcriptional signature (PMN-MDSC-sig) correlates with NLRP3-tsig in the melanoma (SKCM) TCGA. (**D**) Receiver operating characteristic (ROC) curve analysis of the NLRP3-tsig signature in the Hugo et al. and Gide et al. datasets of patients with stage IV melanoma treated with anti–PD-1 immunotherapy. AUC, area under the curve. (**E**) ROC curve analysis comparing the NLRP3-tsig signature with PMN-MDSC-sig and M-MDSC-sig transcriptional signatures in the Hugo et al. dataset. (**F**) ROC curve analysis comparing the NLRP3-tsig signature with previously published transcriptional signatures in the Hugo et al. dataset. (**G**) ROC curve analysis comparing the NLRP3-tsig signature with previously published anti–PD-1 resistance transcriptional signatures in the Lee et al. dataset of patients with stage IV melanoma treated with anti–PD-1 immunotherapy. (**H**) Gene Expression Profiling Interactive Analysis (GEPIA) utilized to analyze the disease-free survival of patients with melanoma in TCGA (SKCM) based on the NLRP3-tsig transcriptional signature (high, above the median; low, below the median). Statistical analysis performed by log-rank test. ***P < 0.0005.

**Figure 3:**
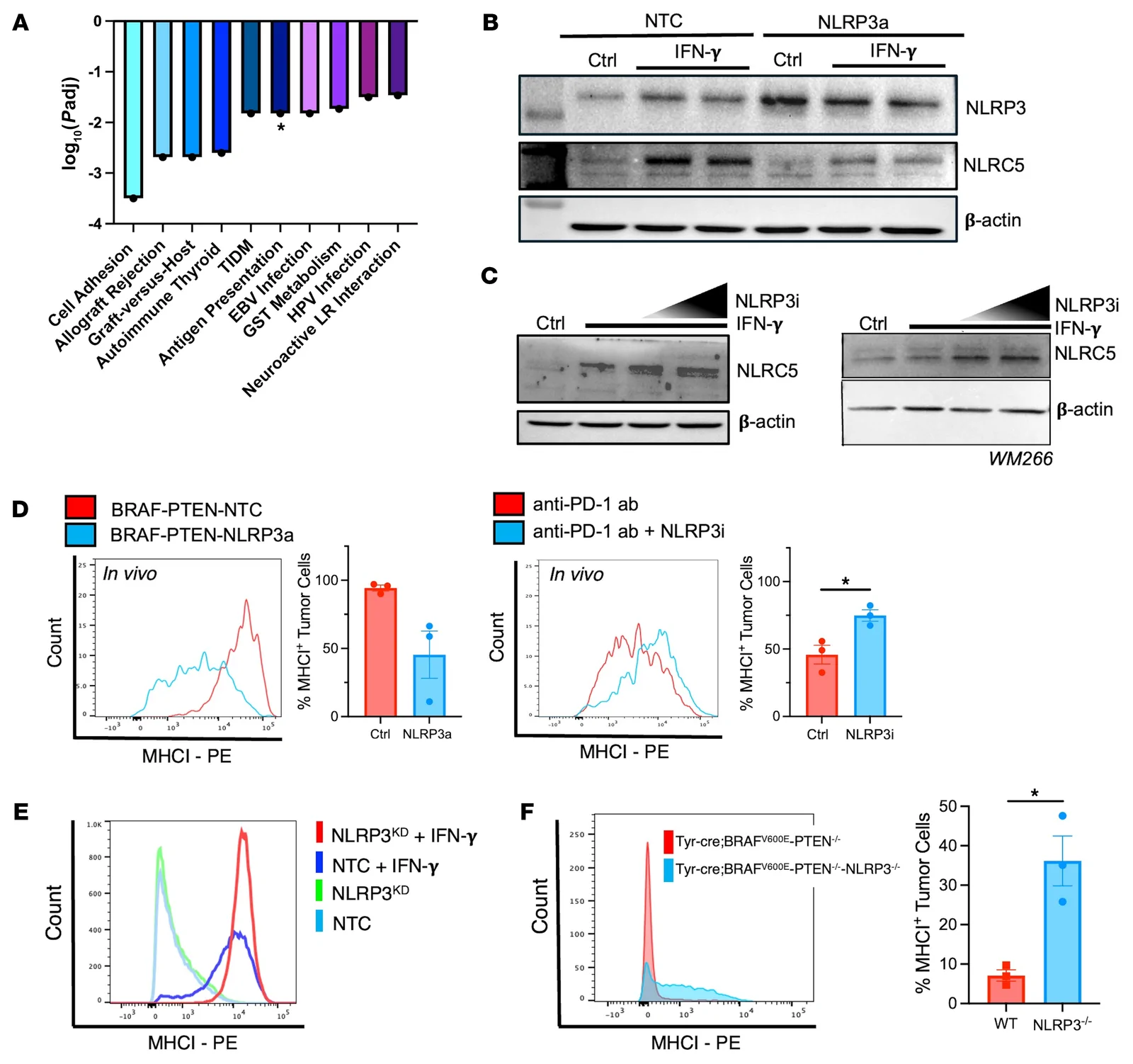
Genetic and pharmacologic inhibition of NLRP3 in tumors enhances NLRC5 and MHC class I expression levels. (**A**) Most downregulated pathways in the *Nlrp3*-amplified BRAF^V600E^ PTEN^–/–^ melanoma cell line (BRAF^V600E^ PTEN^–/–^ NLRP3a) versus the parental BRAF^V600E^ PTEN^–/–^ melanoma cell line based on KEGG pathway analysis of RNA-seq data (*n* = 2). KEGG, Kyoto Encyclopedia of Genes and Genomes. (**B**) NLRP3 and NLRC5 Western blot analysis of the BRAF^V600E^ PTEN^–/–^ NTC and BRAF^V600E^ PTEN^–/–^ NLRP3a melanoma cell lines following PBS versus IFN-γ treatment. NTC, nontarget control (*n* = 3). (**C**) Left, NLRC5 Western blot analysis of the BRAF^V600E^ PTEN^–/–^ melanoma cell line following PBS or IFN-γ treatment with increasing concentrations of the NLRP3 inhibitor (NLRP3i), MCC950 (*n* = 3). Right, NLRC5 Western blot analysis of the human WM266 melanoma cell line following PBS or IFN-γ treatment with increasing concentrations of the NLRP3 inhibitor, MCC950 (*n* = 3). (**D**) Left, pan-MHC class I flow cytometry analysis of resected in situ BRAF^V600E^ PTEN^–/–^ NTC and BRAF^V600E^ PTEN^–/–^ NLRP3a melanomas. Right, pan-MHC class I flow cytometry analysis of resected in situ BRAF^V600E^ PTEN^–/–^ NTC melanomas following treatment with anti–PD-1 ab versus anti–PD-1 + MCC950. ab, antibody (*n* = 3). (**E**) pan-MHC class I flow cytometry analysis of BRAF^V600E^ PTEN^–/–^ NTC versus *Nlrp3*-knockdown (KD) BRAF^V600E^ PTEN^–/–^ NLRP3^KD^ cell line following PBS versus IFN-γ treatment (*n* = 3). (**F**) Left, flow cytometry analysis of pan-MHC class I expression levels of resected primary melanomas in the NLRP3^–/–^ and ctrl transgenic melanoma mouse strains. Right, percentage MHC class I–positive tumor cells in the NLRP3^–/–^ and ctrl transgenic melanoma mouse strains (*n* = 3). **D** and **F**: statistical analysis performed using unpaired *t* test. Data expressed as mean values ± SEM. *P < 0.05.

**Figure 4:**
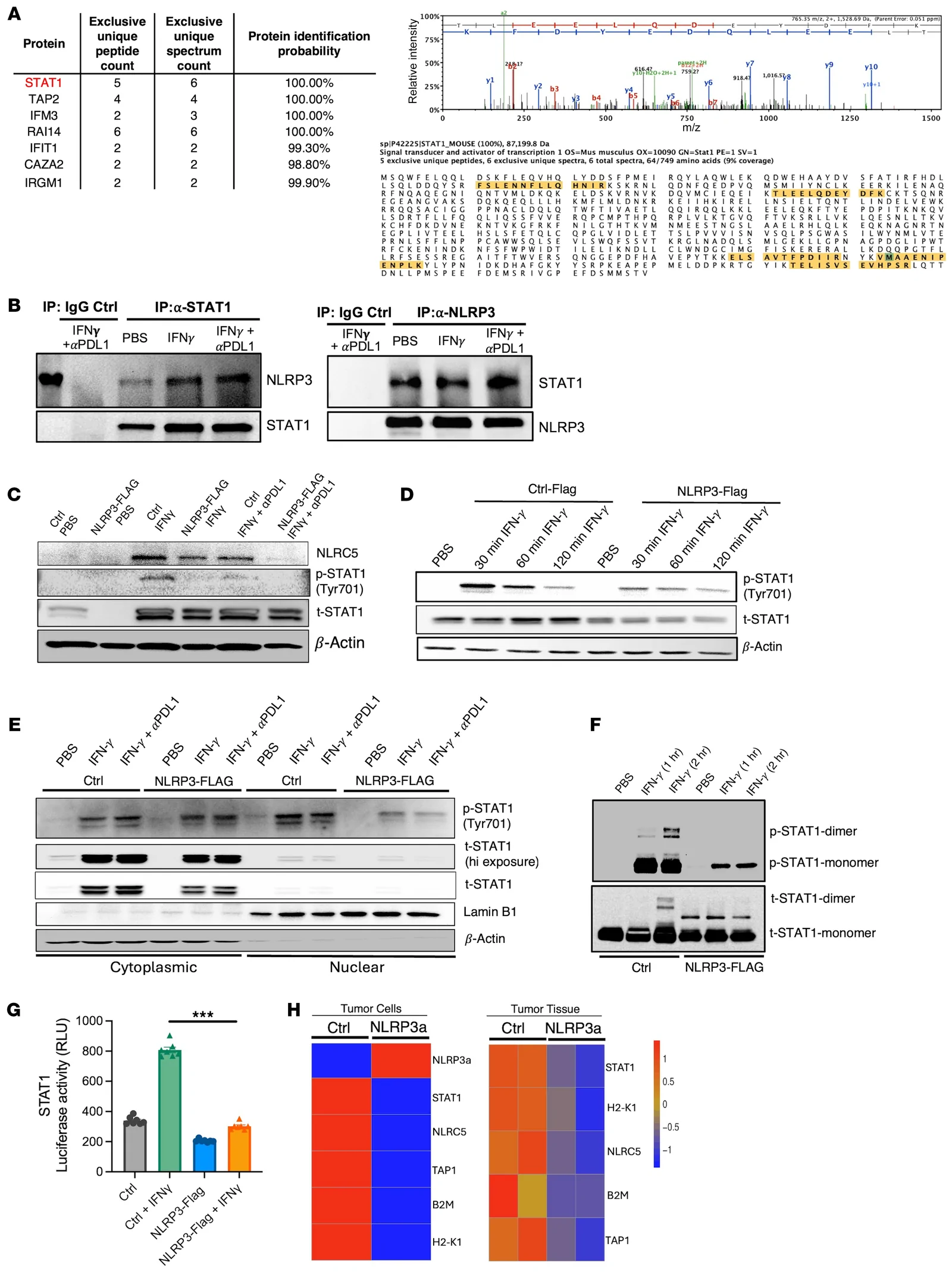
NLRP3 binds to STAT1, inhibits STAT1 dimerization, and suppresses STAT1-dependent transcriptional activation of NLRC5. (**A**) Tandem mass spectrometry identified STAT1 as the most abundant NLRP3-associated protein in tumor cells following IFN-γ + anti–PD-L1 ab treatment (*n* = 2). (**B**) Left, NLRP3 and STAT1 Western blot analysis following STAT1 versus IgG ctrl immunoprecipitation (IP) of BRAF^V600E^ PTEN^–/–^ melanoma cells treated with PBS, IFN-γ, or IFN-γ + anti–PD-L1 ab (*n* = 3). Right, STAT1 and NLRP3 Western blot analysis following NLRP3 versus IgG ctrl IP of BRAF^V600E^ PTEN^–/–^ melanoma cells treated with PBS, IFN-γ, or IFN-γ + anti–PD-L1 ab (*n* = 3). (**C**) NLRC5, phosphorylated STAT1 (Tyr701) (p-STAT1), and total STAT1 (t-STAT1) Western blot analysis of ctrl BRAF^V600E^ PTEN^–/–^ melanoma cells versus NLRP3-FLAG-expressing BRAF^V600E^ PTEN^–/–^ melanoma cells treated with PBS, IFN-γ, or IFN-γ + anti–PD-L1 ab (*n* = 3). (**D**) p-STAT1 and t-STAT1 Western blot analysis of control BRAF^V600E^ PTEN^–/–^ melanoma cells and NLRP3-FLAG-expressing BRAF^V600E^ PTEN^–/–^ melanoma cells following IFN-γ stimulation at various time points (*n* = 3). (**E**) p-STAT1 and t-STAT1 Western blot analysis of cytoplasmic and nuclear lysates isolated from ctrl BRAF^V600E^ PTEN^–/–^ melanoma cells or NLRP3-FLAG-expressing BRAF^V600E^ PTEN^–/–^ melanoma cells treated with PBS, IFN-γ, or IFN-γ + anti–PD-L1 ab (*n* = 3). (**F**) Non-denaturing p-STAT1 and t-STAT1 Western blot analysis of ctrl BRAF^V600E^ PTEN^–/–^ melanoma cells or NLRP3-FLAG-expressing BRAF^V600E^ PTEN^–/–^ melanoma cells treated with PBS or IFN-γ for 1 hour or 2 hours (*n* = 3). (**G**) STAT1 luciferase reporter activity in ctrl BRAF^V600E^ PTEN^–/–^ melanoma cells or NLRP3-FLAG-expressing BRAF^V600E^ PTEN^–/–^ melanoma cells treated with either PBS or IFN-γ (*n* = 3). RLU, relative light units. Statistical analysis based on a 2-way ANOVA followed by Tukey’s multiple comparison test. (**H**) Left, RNA-seq analysis of ctrl BRAF^V600E^ PTEN^–/–^ and BRAF^V600E^ PTEN^–/–^ NLRP3a melanoma cells. Right, RNA-seq analysis of FACS-sorted CD90.2^–^CD31^–^CD45^–^ ctrl BRAF^V600E^ PTEN^–/–^ and BRAF^V600E^ PTEN^–/–^ NLRP3a melanoma tumor cells (*n* = 2). All data expressed as mean values ± SEM. ***P < 0.0005.

**Figure 5:**
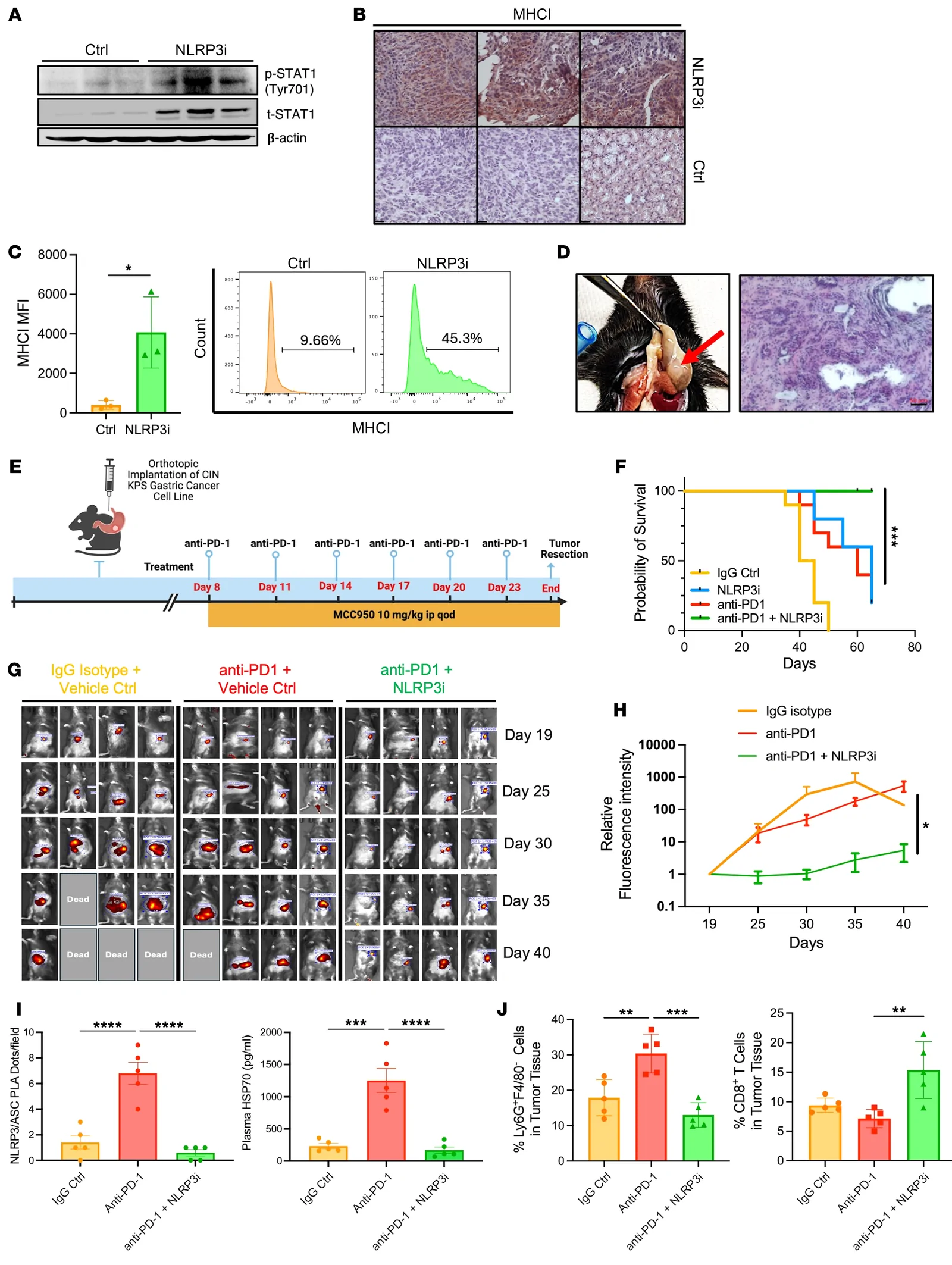
Inhibiting NLRP3 overcomes resistance to anti–PD-1 Immunotherapy in an orthotopic model of CIN gastric adenocarcinoma. (**A**) p-STAT1 and t-STAT1 Western blot of whole tumor tissue lysate following treatment of orthotopic KPS CIN gastric tumors with vehicle control (ctrl) versus MCC950 (NLRP3i) (*n* = 3). (**B**) Pan-MHC class I IHC analysis of tumor tissues derived from orthotopic KPS CIN gastric tumors following treatment with either NLRP3i versus vehicle ctrl (*n* = 3). Representative images shown. Scale bar = 20 μm. (**C**) Pan-MHC class I flow cytometry analysis of FACS-sorted CD90.2^–^CD31^–^CD45^–^ ctrl tumor cells following treatment of orthotopic KPS CIN gastric tumors with either NLRP3i versus vehicle ctrl. Left, representative pan-MHC class I flow histograms. Statistical analysis based on unpaired *t* test (*n* = 3). (**D**) Gross image of an orthotopic KPS CIN gastric tumor. Red arrow, implanted tumor cells in the gastric mucosa. Right, representative H&E microscopy of an orthotopic KPS CIN gastric tumor. Scale bar = 50 μm. (**E**) Schematic describing the anti–PD-1/NLRP3i treatment schedule for the orthotopic KPS CIN gastric cancer model. (**F**) Survival analysis of mice harboring orthotopic KPS CIN gastric tumors following treatment with IgG isotype control, anti–PD-1 alone, NLRP3i alone, versus combination anti–PD-1/NLRP3i (*n* = 10 mice/group). Statistical analysis based on log-rank test. (**G**) IVIS monitoring of orthotopic tdTomato-expressing KPS gastric tumors in syngeneic mice while undergoing IgG isotype control, anti–PD-1 alone, versus combination anti–PD-1/NLRP3i therapy. (**H**) IVIS quantification of tdTomato-expressing KPS gastric tumors while undergoing IgG isotype control, anti–PD-1 alone, versus combination anti–PD-1/NLRP3i therapy over time (*n* = 8 mice/group). (**I**) Left, NLRP3-ASC PLA of orthotopic KPS CIN gastric tumors following treatment with IgG isotype ctrl, anti–PD-1 ab, and anti–PD-1/NLRP3i. Right, plasma HSP70 ELISA performed on day 25 following treatment with IgG isotype ctrl, anti–PD-1 ab, and anti–PD-1/NLRP3i (*n* = 5 mice/group). (**J**) Left, flow cytometry analysis of tumor-infiltrating CD11b^+^Ly6G^+^F4/80^–^ PMN-MDSCs following treatment with IgG isotype ctrl, anti–PD-1 ab, and anti–PD-1/NLRP3i. Right, flow cytometry analysis of tumor-infiltrating CD8^+^ T cells following treatment with IgG isotype ctrl, anti–PD-1 ab, and anti–PD-1/NLRP3i (*n* = 5 mice/group). (**H**) Statistical analysis performed using 2-way ANOVA followed by Tukey’s multiple-comparison test. (**I** and **J**) Statistical analysis performed using a 1-way ANOVA followed by Tukey’s multiple-comparison test. All data expressed as mean values ± SEM. *P < 0.05, **P < 0.005, ***P < 0.0005, ****P < 0.00005.

**Figure 6:**
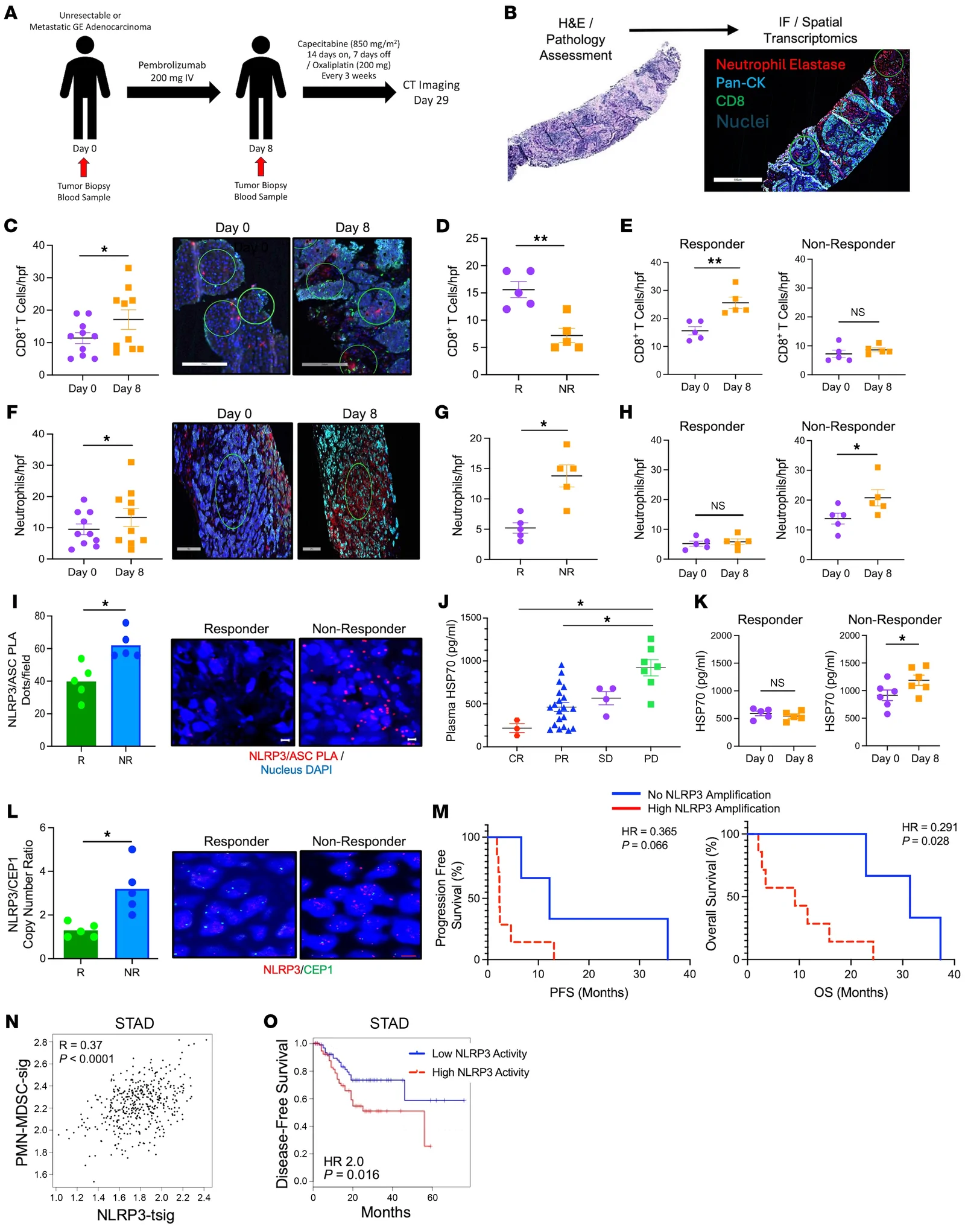
Elevated tumor-intrinsic NLRP3 activity and *NLRP3* amplification correlate with anti–PD-1 resistance in patients with advanced gastroesophageal adenocarcinoma. (**A**) KeyLARGO clinical protocol included a pembrolizumab-only lead-in phase. Data collected from 5 responding (R) and 5 non-responding (NR) patients. (**B**) H&E, IF, and spatial transcriptomics workflow on GE adenocarcinoma tissue specimens. (**C**) Left, mean CD8^+^ T cells in ROIs day 0 and day 8. Original magnification, 40×. ROIs, regions of interest. Right, representative ROIs in a responder on day 0 and day 8. Scale bar = 200 μm. Green, CD8. Cyan, pan-cytokeratin (pan-CK). Red, elastase. Dark blue, nuclei. (**D**) Baseline mean CD8^+^ T cells in ROIs based on CD8 IF collected from R and NR. (**E**) Mean CD8^+^ T cells in ROIs based on CD8 IF on day 0 and day 8 from R (left) and NR (right). (**F**) Left, mean neutrophils in ROIs based on elastase IF collected on day 0 and day 8. Right, representative ROIs in an NR patient on day 0 and day 8. Scale bar = 200 μm. (**G**) Baseline mean neutrophils in ROIs based on elastase IF collected from R and NR. (**H**) Mean neutrophils in ROIs based on elastase IF collected on day 0 and day 8 from R (left) and NR (right). (**I**) Left, baseline NLRP3-ASC PLA dots per hpf collected from R and NR. Right, representative PLA IF field in R and NR. Scale bar = 5 μm. (**J**) Baseline plasma HSP70 levels categorized according to best objective response. CR, complete response; PR, partial response; SD, stable disease; PD, progressive disease. One-way ANOVA followed by Tukey’s multiple comparisons test. (**K**) Baseline HSP70 levels collected on day 0 and day 8 from R (left) and NR (right). (**L**) NLRP3/CEP1 copy number ratio based on FISH collected from R and NR. Right, representative IF field in R and NR. Red, NLRP3 probe. Green, chromosome enumeration probe for chromosome 1 (CEP1). Original magnification, 100×. Scale bar = 5 μm. (**M**) PFS (left) and OS (right) stratified based on NLRP3 amplification status. Log-rank test. (**N**) ssGSEA used to correlate PMN-MDSC-sig with NLRP3-tsig in the gastric cancer (STAD) TCGA. (**O**) Disease-free survival of patients with gastric cancer in TCGA (STAD) based on the NLRP3-tsig transcriptional signature (High, above the median; Low, below the median). Log-rank test. All other statistical analyses performed using unpaired *t* tests. Data expressed as mean values ± SEM. *P < 0.05, **P < 0.005.

**Figure 7:**
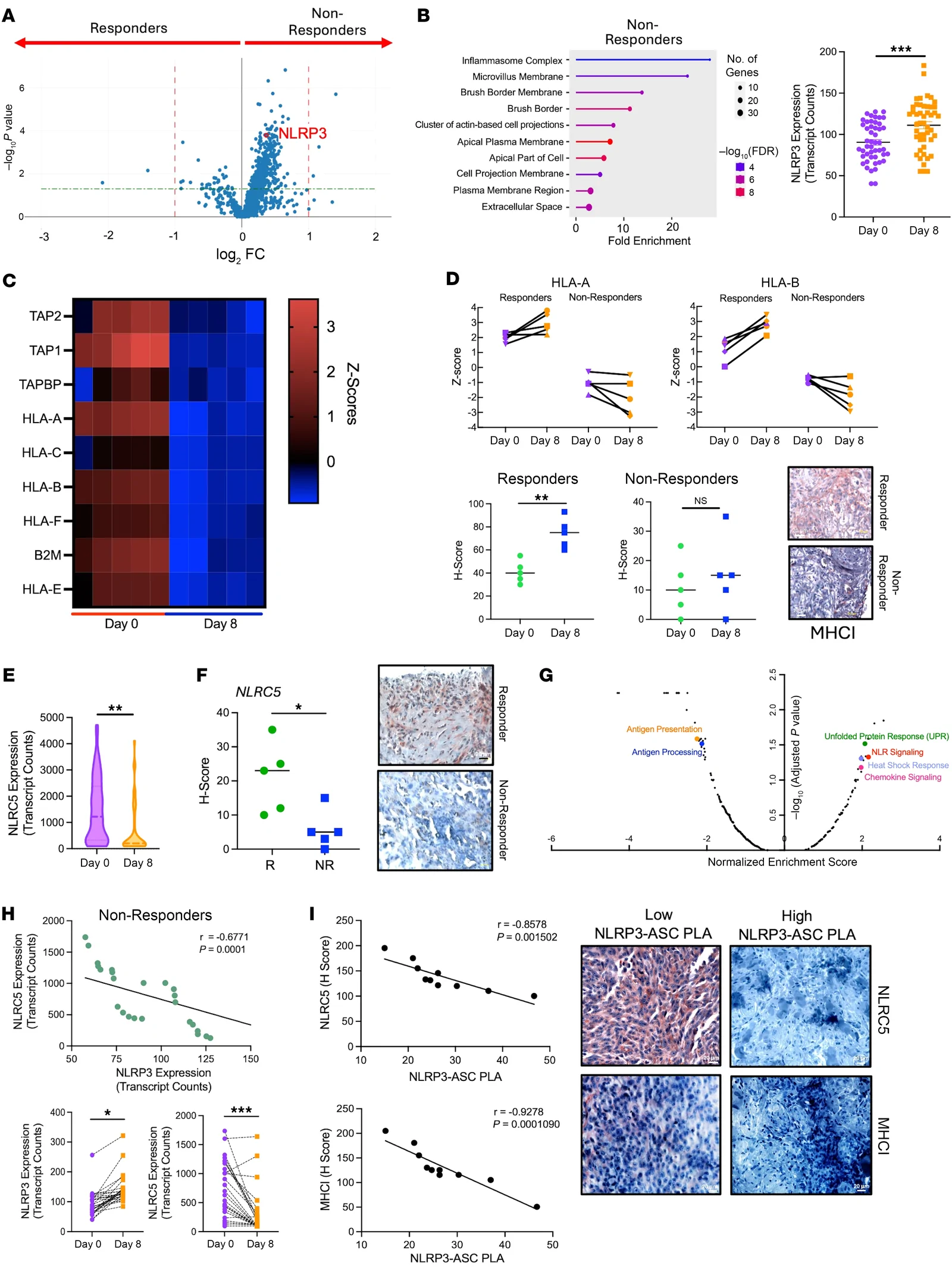
Tumor NLRP3 expression inversely correlates with diminished NLRC5 and pan-MHC class I expression in nonresponding patients with GE adenocarcinoma. (**A**) Volcano plot of spatial transcriptomic (ST) analysis of all selected ROIs in responders and nonresponders. (**B**) Left, KEGG pathway analysis of ST data derived from nonresponders. Right, *NLRP3* transcript counts per ROI in tumor tissues collected on day 0 and day 8. (**C**) Heatmap of select mean MHC class I–associated transcript levels collected on day 0 and day 8 from nonresponders. Right, *Z*-scores. (**D**) Top, tumor *HLA-A* and *HLA-B* expression levels based on *Z*-score derived from the same responding and nonresponding patients on day 0 and day 8. Bottom, H-scores determined for pan-MHC class I expression based on IHC on day 0 and day 8 tumor tissues derived from responders and nonresponders. Right, representative pan-MHC class I IHC images. Scale bar = 20 μm. (**E**) *NLRC5* transcript counts per ROI in tumor tissues collected on day 0 and day 8. (**F**) H-scores determined for NLRC5 expression based on IHC of tumor tissues derived from responders (R) and nonresponders (NR). Right, representative NLRC5 IHC images. Scale bar = 20 μm. (**G**) Volcano plot of KEGG pathway ST analysis of nonresponding tumor tissues. (**H**) Top, Spearman’s correlation of *NLRC5* and *NLRP3* expression levels based on ROI ST analysis of nonresponding tumor tissue specimens. Bottom, tumor *NLRP3* and *NLRC5* expression levels derived from the same patients on day 0 and day 8. (**I**) Left, Spearman’s correlation of NLRP3-ASC PLA levels with NLRC5 and pan-MHC class I H-scores in human melanoma tissues. Right, representative images of NLRC5 and pan-MHC class I IHC analysis of human melanoma tissues with low and high NLRP3-ASC PLA levels. Scale bar = 20 μm. **B**, **D**–**F**, and **H**: Statistical analysis performed using unpaired *t* tests. Data expressed as mean values ± SEM. *P < 0.05, **P < 0.005. ***P < 0.0005.

## Version 1 08/13/2026

In-Press Preview

## Version 2 10/15/2026

Electronic publication
