# Supplementary material for: Tumor NLRP3 amplification promotes immunotherapy resistance by inhibiting STAT1 signaling and MHC class I expression
Source: J Clin Invest. 2026 Aug 13;136(20):e201816. doi: 10.1172/JCI201816 (PMC13668618; doi:10.1172/JCI201816)
Supplement: Supplementary file 1 [file jci-136-201816-s133.pdf]

Supplemental Materials For

Tumor *NLRP3* Amplification Promotes Immunotherapy Resistance by Inhibiting STAT1 Signaling and MHC Class I Expression

Balamayroon Theivanthiran[1], Nagendra Yarla[2], Kaylee Villarreal[2], Y-Van Nguyen[1], Mahere Rezazade Bazaz[1], Ernesto Pena Calderin[1], Linda Cao[1], Kyra Majors[1], Michael P. Plebanek[1], Alisha Holtzhausen[3], Emily Bolch[2], Douglas B. Johnson[4], Hope Uronis[2], John H. Strickler[2], Nicholas C. DeVito[2], Brent A. Hanks[1,3§].

[1]Lineberger Comprehensive Cancer Center, Department of Medicine, Division of Medical Oncology, Department of Microbiology and Immunology
University of North Carolina, Chapel Hill, NC 27599 USA

[2]Duke Cancer Institute, Department of Medicine, Division of Medical Oncology
Duke University , Durham, NC 27710 USA

[3]Department of Pharmacology, University of North Carolina, Chapel Hill, NC 27599 USA

[4]Vanderbilt University Medical Center, Department of Medicine, Nashville, TN USA

[§]Corresponding Author: Lineberger Comprehensive Cancer Center, Department of Medicine, Division of Medical Oncology, Department of Microbiology and Immunology, Department of Pharmacology
University of North Carolina , 450 West Drive, CB# 7295, Chapel Hill, NC 27599-7295, E-mail: Brent_Hanks@med.unc.edu

This PDF file includes:

Supplemental Methods : Pages 2 – 9

Supplemental Figures 1 - 8 : Pages 10 – 19

Supplemental Tables 1 - 7 : Pages 20 – 27

Supplemental Methods

Clinical Samples

Patients provided written informed consent for use of biological specimens on institutional review board (IRB)-approved protocols at Duke Cancer Institute (NCT02694965, NCT03342937) or at Vanderbilt University Medical Center (Institutional Protocol #: 100178). Baseline formalin-fixed paraffin-embedded (FFPE) tissues and plasma specimens were collected from 52 patients with untreated stage IV melanoma prior to initiating anti-PD-1 monotherapy or ipilimumab/nivolumab combination immunotherapy at either Duke Cancer Institute or Vanderbilt University Medical Center (**Supplementary Table 1**). Treatment response at week 12 and every 12 weeks thereafter was determined based on independent radiologic review of computed tomography (CT) imaging using RECIST1.1 (Response Evaluation Criteria in Solid Tumors v1.1) criteria. Patients were considered responders (R) if they demonstrated complete or partial response and considered non-responders (NR) if they demonstrated progressive disease. All patients were monitored for disease recurrence for a total of 5 years. Age, gender, disease stage, number of involved organs, BRAF mutation status, and treatment were evaluated as covariates (**Supplementary Table 2**). Baseline FFPE tissues and plasma specimens were collected from 13 patients with untreated stage III melanoma prior to initiating adjuvant anti-PD-1 monotherapy at Duke Cancer Institute (**Supplementary Table 3**). Patients underwent CT imaging every 12 weeks and were monitored for disease recurrence for a total of 5 years. Baseline FFPE tissues and plasma specimens were collected from 35 patients with unresectable stage III or stage IV GE adenocarcinoma at Duke Cancer Institute prior to initiating pembrolizumab on the IRB-approved KeyLARGO clinical trial (NCT03342937) (**Supplementary Table 4**). Repeat tissue biopsies and plasma specimens were collected on day 8 of treatment prior to initiating capecitabine/oxaliplatin chemotherapy. All collected tumor tissues were reviewed by a board-certified pathologist prior to analysis. Following pathology review, 10 paired GE adenocarcinoma tissue specimens were utilized for spatial transcriptomic analysis. PFS and best objective response (BOR) were recorded based on day 29 CT imaging followed by subsequent CT imaging every 12 weeks. Assessment of PFS and best overall response rate (BORR) were the primary clinical objectives of this study.

Animal Models

C57BL/6J (C57, H-2^{b}) (Stock #: 000664) and B6.CgBraf^{tm1Mmcm}Pten^{tm1Hwu}Tg(Tyr-cre/ERT2)13Bos/BosJ (Braf^{V600E}Pten$^{-/-}$, H-2^{b}) (Stock #: 012328) mice were purchased from Jackson Labs. C57BL/6-Nlrp3$^{tm1.1Mrl}$ (NLRP3$^{flox/flox}$, Stock #: 12809) were obtained from Taconic. The NLRP3$^{flox/flox}$ mice were crossed with B6.Cg-*Braf*^{tm1Mmcm} *Pten*^{tm1Hwu} Tg(Tyr-cre/ERT2 H-2^{b})13Bos/BosJ transgenic mice to produce a melanoma tissue-specific *Nlrp3* conditional knockout (Tyr-cre;Braf^{V600E}Pten$^{-/-}$;Nlrp3$^{flox/flox}$). NLRP3 silencing was verified by PCR and immunoblotting. All experimental groups consisted of randomly chosen littermates of both sexes, aged 6 to 10 weeks, from the same strain. All animal procedures followed protocols approved by the Institutional Animal Care and Use Committee at either Duke University Medical Center or the University of North Carolina at Chapel Hill.

Autochthonous Tumor Studies

B6.Cg-*Braf*^{tm1Mmcm} *Pten*^{tm1Hwu} Tg(Tyr-cre/ERT2 H-2^{b})13Bos/BosJ and Tyr-cre;Braf^{V600E}Pten$^{-/-}$;Nlrp3$^{flox/flox}$ transgenic mice were subdermally injected with 4-Hydroxytomoxifen (4-HT) (Sigma-Aldrich, H6278-50MG CCF, 38.75 µg/mouse) to induce primary melanoma development at the base of the tail. Mice were randomly assigned to treatment cohorts when tumor volumes reached 64 mm^3. For select experiments, mice were treated with the following agents: NLRP3 inhibitor, MCC950 (Invivogen, inh-mcc) 10 mg/kg i.p. injection every other day, anti-PD-1 antibody (BioXCell, BE0146) or rat IgG2a isotype control (BioXCell, BE0089) at 200 µg i.p. every 3 days, anti-Ly6G antibody (BioXCell, BE0075-1), initial dose at 200 µg/mouse followed by 100 µg/mouse/day x 2. Primary tumor volumes were monitored by orthogonal caliper measurements every 3 days. Tumor volume was calculated according to the formula: $cm^3 = [(\text{length, cm}) \times (\text{width, cm})^2]/2$.

Syngeneic and Orthotopic Tumor Studies

BRAF^{V600E}PTEN$^{-/-}$, BRAF^{V600E}PTEN$^{-/-}$NLRP3a, and BRAF^{V600E}PTEN$^{-/-}$NTC control cell lines (0.5×10^5 to 1×10^6 cells) were implanted by subcutaneous injection into the base of the tail or chest of syngeneic C57BL/6 mice (NTC, non-target control). Chromosomal instability (CIN) Kras$^{G12D/+}$Tp53$^{R172H/+}$Smad4$^{-/-}$ (KPS) gastric cancer cell line, KPS-NLRP3a cell line, and KPS-NTC control cell line (0.5×10^6 cells) in 50 µl of cell suspension with 50% Matrigel were injected either orthotopically into the subserosa of the gastric wall or implanted subcutaneously in syngeneic C57BL/6 mice (0.5×10^5 to 1×10^6 cells) (28). Subcutaneous tumor growth was monitored using caliper measurements every 3 days and treatment was initiated when tumor volumes reached either 64 mm^3 or 90 mm^3, depending on the study. Surgical procedures were performed under a small animal heat lamp and post-surgery recovery was monitored closely. Surgical wounds were managed with surgical clips and topical iodine solution. Treatment consisting of either anti-PD-1 (200 µg i.p. every 3 days), MCC950 (10 mg/kg i.p. injection qod), or both was initiated on day 8 post-implantation. Orthotopic tumor growth was measured based on caliper and stomach weight on day 25. Additional studies were performed for 60 days with survival as the primary end-point. Mice were sacrificed if they met Institutional Animal Care and Use Committee (IACUC)-defined humane endpoints. A tdTomato-expressing KPS gastric cancer cell line was generated and implanted into the subserosa of the gastric wall of syngeneic mice. Tumor growth was monitored using an In Vivo Imaging System (IVIS) Lumina at the UNC Biomedical Research Imaging Center (BRIC). When tumors reached a detectable size and exhibited similar fluorescence intensity, animals were grouped for treatment. Mice were treated with either a rat IgG2a isotype control, anti-PD-1 (200 µg i.p. every 3 days), or a combination of anti-PD-1 every 3 days with MCC950 (20 mg/kg i.p. daily). Relative fluorescence intensities were calculated and used to monitor gastric tumor growth for each treatment arm over 40 days.

Cell Lines and Culture Conditions

$BRAF^{V600E}PTEN^{-/-}$NLRP3a and $BRAF^{V600E}PTEN^{-/-}$NTC cell lines as well as the KPS-NLRP3a and KPS-NTC cell lines were engineered using the CRISPR amplification technique as previously described (6). The NLRP3 and control CRISPRa plasmids (Santa Cruz, sc-432122-ACT) were packaged into a lentiviral vector in HEK293T cells as previously described. Clones were selected using a combination of antibiotics, including puromycin (Santa Cruz, sc-108071), hygromycin B (Santa Cruz, sc-29067), and blasticidin S HCL (Santa Cruz, sc-495389). The $BRAF^{V600E}PTEN^{-/-}$ (male, BPD6) melanoma cell line was derived from the transgenic $BRAF^{V600E}PTEN^{-/-}$ melanoma model as previously described (45, 46). The $BRAF^{V600E}PTEN^{-/-}NLRP3^{KD}$ and $BRAF^{V600E}PTEN^{-/-}$NTC cell lines were derived from the $BRAF^{V600E}PTEN^{-/-}$ cell line as previously described [54]. Stable cell lines were selected based on puromycin resistance (Sigma-Aldrich, P8833). The $BRAF^{V600E}PTEN^{-/-}$NLRP3-F and the $BRAF^{V600E}PTEN^{-/-}$Flag control cell lines were generated using the pcDNA3-N-Flag-NLRP3 (Addgene, 75127) and pcDNA3-Flag (Addgene, 20011) plasmids, respectively. The $BRAF^{V600E}PTEN^{-/-}$STAT1-GFP cell line was created using the pLenti-C-STAT1-mGFP-P2A-Puro vector (Addgene, 199345). Drs. Therese Seidlitz and Daniel E Stange (Carl Gustav Carus Unversitätsklinikum, Dresden, Germany) generously provided the CIN KPS gastric cell line. All $BRAF^{V600E}PTEN^{-/-}$ cell lines were maintained at 37°C in Dulbecco's Modified Eagle Medium (DMEM, Invitrogen) with 2 mM L-glutamine, supplemented with 10% fetal bovine serum (FBS) and 100 units/ml penicillin. The CIN KPS cell line was cultured in advanced DMEM medium with 10% FBS, glutamine, and 1x B27 supplement. For select *in vitro* experiments, cell lines were treated with IFN-γ (100 ng/ml, BioAbChem, 42-IFNg), anti-PD-L1 antibody, clone 10F.9G2 (1 to 2 μg/ml, BioXCell, BE0101), MCC950 NLRP3 inhibitor (2.5 μM to 10 μM, Invivogen, inh-MCC), or Caspase-1 inhibitor (1 μg/ml, Invivogen, inh-yvad). The tdTomato-expressing KPC cell line was generated using a lentiviral transfer plasmid encoding a mammalian codon-optimized tdTomato coding sequence downstream of the spleen focus-forming virus (SFFV) promoter. Following transfection using Lipofectamine 3000, tdTomato-expressing KPC cells were isolated using a Sony SH800 Sorter and tdTomato expression was verified by flow cytometry prior to performing orthotopic tumor experiments. The KPCY murine pancreatic cell line (Kerafast, EUP014-FP) was derived from the $Kras^{LSL\text{-}G12D/+}$;$Trp53^{LSL\text{-}R172H/+}$;Pdx1-Cre;$Rosa26^{YFP/YFP}$ mouse model and cultured in DMEM with 10% fetal bovine serum (FBS) and Glutamax (47). All cell lines were screened annually for mycoplasma contamination by the Duke University and University of North Carolina Tissue Culture Facilities.

RNA Isolation and qRT-PCR Analysis

Tissue specimens were harvested from tumors and stored in RNAlater at –80°C. Tissues were lysed in RLT buffer by using a gentleMACS dissociator (Miltenyi Biotec) and cell lines were lysed in RLT buffer and stored at –80°C. Total RNA was isolated with the RNeasy Plus Mini Kit (Qiagen, 74134) and quantified using a NanoDrop. RNA (500 ng to 1000 ng) was used for cDNA synthesis with the iScript Reverse Transcription Supermix (BioRad, 1708841). qRT-PCR was performed on an ABI7500 Real-Time PCR system (Life Technologies). All qRT-PCR reactions were performed using validated primers (**Supplementary Table 6**) and SsoAdvanced Universal SYBR Green Super Mix (BioRad, 1725271) or SsoAdvanced Universal Probes Supermix (BioRad, 1725281). All data were normalized to b-actin expression and relative gene expression was quantified using the 2^{-DDCt} method.

Western Blot Analysis

Tissues or cells were lysed in NP40 lysis buffer (Sigma-Aldrich) supplemented with a complete protease inhibitor and phosphatase inhibitor (Roche). After lysing in Laemmli sample buffer, equal volumes of lysates were separated using 10% or 15% SDS–PAGE and then transferred to a PVDF membrane (Bio-Rad Laboratories Inc., 162017). After blocking for 30 minutes in tris-buffered saline (TBS) containing 0.1% Tween-20 and 5% milk, the membranes were probed with various primary antibodies followed by horseradish peroxidase (HRP)-conjugated secondary antibodies. Primary antibodies included: Anti-β-actin mouse monoclonal (clone c4, 1:3000, Santa Cruz Biotechnology, sc-47778), anti-NLRP3 rabbit monoclonal (clone D4D8T, 1:1000, Cell Signaling, 15101S; clone EPR23073-96, 1:1000, Abcam, 270499), anti-Caspase-1-p20 mouse monoclonal (clone Casper-1, 1:500, Adipogen, AG-20B-0042-C100), anti-HSP70 mouse monoclonal (clone C92F3A-5, 1:1000, Santa Cruz Biotechnology, sc-66048). anti-NLRC5 mouse polyclonal (clone IN113 ,1:500, Adipogen, AG-25B-0038-C100). Anti-STAT1 rabbit monoclonal (clone D1K9Y, 1:1000, Cell Signaling, 14994S), anti-P-STAT1 rabbit monoclonal (clone 58D6, 1:1000, Cell Signaling, 9167S), and anti-Lamin B1 mouse monoclonal (clone B-10, Santa Cruz Biotechnology, SC-374015). Immunoreactivity was visualized using a chemiluminescence substrate (Thermo Fisher Scientific, 34095/34075) and imaged with a ChemiDoc XRS+ System (Bio-Rad).

Co-immunoprecipitation Studies

Cells were lysed using IP lysis buffer (Thermo Fisher, 87787). Either Anti-NLRP3 antibody (Abcam, 270449) or Anti-STAT1 (Cell Signaling, 14994S), along with IgG isotype (Cell Signaling, 3900), was conjugated with Dynabeads Protein A beads (Thermo Fisher, 10006D). These conjugates were added to the lysate and processed according to the manufacturer's protocol. The NLRP3-bound and isotype-bound beads were pulled down using magnetic isolation techniques. After washing, proteins from the beads were eluted and analyzed by Western blot, then probed for either Anti-STAT1 (Cell Signaling, 14994S) or Anti-NLRP3 antibody (Abcam, 270449).

Liquid Chromatography–Tandem Mass Spectrometry

Target proteins were immunoprecipitated using magnetic beads coated with anti-NLRP3 or isotype control antibodies. Beads were washed 5 times with 50 mM ammonium bicarbonate, followed by digestion with 20 μL of 20 ng/μL trypsin overnight. An additional 20 μL of trypsin was added, and supernatants were recovered after 4 hours. Digests were adjusted to 70 μL with 0.1% TFA and then subjected to Stagetip cleanup. After lyophilization, peptides were resuspended in 30 μL, and 5 μL of each sample was analyzed using a 90-minute FAIMS-LC-MS/MS method on Thermo Fusion Lumos MS at the Duke Proteomics and Metabolomics Shared Resource. Data were processed using FragPipe 16 and visualized in Scaffold v5.3.4 (Portland, OR).

ELISA

Human plasma HSP70 levels were measured in a blinded fashion using the human HSP70 DuoSet assay (R&D Systems, DY1663). All ELISAs were performed according to the manufacturer's protocol.

Flow Cytometry Analysis

Cells (1 to 2 x 10^6) were diluted in 150 µl flow buffer per well of V bottom culture plates, incubated with Live/Dead Fixable Violet Dead Cell Stain Kit (0.5 ml/1 x 10^6 cells, Thermo Fisher Scientific, L34964A) on ice for 20 minutes, washed twice, and incubated with Fc Block (anti-CD16/CD32, 2 µg/mL) before staining with appropriate conjugated antibodies. Cells were washed, resuspended in 2% paraformaldehyde for 15 minutes at 4°C, and analyzed using a BD FACSCanto flow cytometry system. Compensation was performed using fluorescence minus one (FMO) controls. Cells were characterized using the following combinations of cell surface markers after gating on viable single cell populations. All antibodies were obtained from commercial vendors and utilized at 1 mg / 1 x 10^6 cells: Anti-Mouse CD11b, phycoerythrin (PE)-conjugated, clone: MIH5 (BD Pharmingen, 558091), Anti-Mouse Ly6G-GR1, fluorescein isothiocyanate (FITC)-conjugated, clone: RB6-8C5 (BD Pharmingen, 5532127), Anti-Mouse Ly6G, FITC-conjugated, clone: 1A8 (BioLegend,127605), Anti-Mouse Ly-6G, FITC-conjugated, clone: 1A8-Ly6g (Thermo Fisher, 11-9668-82), Anti-Mouse F4/80, APC-conjugated, clone: BM8 (BD Pharmingen, 560408), Anti-Mouse CD45, PerCP-Cy5.5-conjugated, clone: 145-2C11 (BD Pharmingen, 551163), Anti-Mouse CD90.2, FITC-conjugated, clone: 53-2.1 (BD Bioscience, 553003), Anti-Mouse CD31, clone: 390 (Biolegend 102406), FITC-conjugated, clone: 53-2.1 (BD Bioscience, 553003), Anti-Mouse CD8α, brilliant violet (BV) 510-conjugated, clone: 53-6.7 (BD Pharmingen, 563068), Anti-Mouse CD3ε, PerCP-Cy5.5-conjugated, clone: 145-2C11 (BD Pharmingen, 551163), Anti-Mouse CD8α, APC-conjugated, clone: 53-6.7 (BD Bioscience, 553035), Anti-Mouse Ly6C, PE-Cy7-conjugated, clone: AL-21 (BD Pharmingen, 560593), Anti-Mouse CD119, PE-conjugated, clone: 2E2 (Thermo Fisher,12-1191-82). Cell populations were characterized based on the following marker profiles: Ly6G+ PMN-MDSCS: $CD45^+CD11b^+Ly6G^{hi}Ly6C^{lo}F4/80^-$; Tumor cells : $CD45^-CD90.2^-CD31^-$; tumor-associated macrophages: $CD45^+CD11b^+Ly6G^+F4/80^+$. FACS was performed using a Beckman Astrios Cell Sorter at cell densities of 1 x 10^6 cells/mL in 1% RPMI supplemented with penicillin/streptomycin. All flow cytometry data was analyzed using FlowJo software v10.3.

Immunohistochemistry Analysis

Paraffin sections (5-μm) from human GE adenocarcinoma and mouse gastric cancer tissues were processed using standard protocols for immunohistochemistry (IHC) and immunofluorescence (IF) staining. Tissues were permeabilized by incubation in 0.4% Triton-X in TBS for 20 minutes. The following primary antibodies were incubated for 12-18 hours at 4°C: anti-NLRC5, rabbit polyclonal (clone: 84166, 1:200, Novus Biologicals, NBP3-17901), anti-NLRC5, rabbit polyclonal (clone: AB_2900294, 1:100, ThermoFisher, PA5-115659), anti-pan-MHC class I, rabbit monoclonal (clone: E8E7N, 1:100, Cell Signaling, 76828). Pan-MHC class I and NLRC5 expression levels were analyzed using ImageJ software based on six to eight random fields averaged per section across three to four sections per specimen. The staining intensity for all markers was scored as 0 (no staining), 1 (weak staining), 2 (moderate staining), or 3 (strong staining) and calculated based on the following formula: H-score = ((1 x # weakly stained cells) + (2 x # moderately stained cells) + (3 x # of strongly stained cells))/# total cells(48).

Spatial Transcriptomics

Paired human GE adenocarcinoma FFPE tissue specimens (10) were analyzed on the Nanostring GeoMx digital spatial profiling (DSP) platform (Bruker, Seattle, WA) at the UNC Pathology Services Core (PSC) Facility. FFPE tissue samples were prepared on positively charged slides. Pre-treatment was carried out using the Bond III Automated IHC Staining System (Leica Biosystems, Deer Park, IL) and the following primary antibodies: anti-CD8, rabbit monoclonal (Cell Marque, 108R-16) 1:500 for 1 hr; anti-pan-CK, mouse monoclonal (Leica Biosystems, AE1/AE3-601-L-U) 1:300 for 30 mins; anti-ELA2, mouse monoclonal (NovusBio, MAB9167AF647) 1:600 for 1 hr. Tyramide signal amplification was performed using Cy3, Alexa488, and Cy5 fluorophores, respectively. Tissues were dewaxed in Bond Dewax solution (Leica Biosystems, AR9222) and hydrated in Bond Wash solution (Leica Biosystems, AR9590). Post-fixation was accomplished using 10% Neutral Buffered Formalin (Electron Microscopy Science, 15740-04). Heat-induced antigen retrieval was performed in Bond-Epitope Retrieval solution 2 pH 9.0 (Leica Biosystems, AR9640) for 20 min, enzymatic retrieval with Bond Enzyme Pre-treatment Kit (Leica Biosystems, AR9551) at 0.1ug/mL for 15 min. Human Whole Transcriptome Atlas probe was hybridized overnight, slides were washed twice in fresh 2X saline-sodium citrate (SSC, Sigma-Aldrich, S6639-1L) and loaded onto the GeoMx DSP. A pan-cytokeratin IF marker was used to identify epithelial tumor tissue while CD8 and elastase IF markers were used to assess tumor-infiltrating $CD8^+$ T cell and PMN/neutrophil cell populations, respectively. Six regions of interests (ROI) were selected at 20X magnification for each tissue slide based on the abundance of each of these markers. The number of $CD8^+$ T cells and PMN/neutrophils within all ROIs were totaled for each tumor specimen. UV light was used to cleave oligos that were then collected in a 96-well plate for library preparation and were sequenced on an Illumina NextSeq2000 by the UNC High-Throughput Sequencing Facility (HTSF). All transcriptional data was analyzed using GeoMx DSP Data Analysis software. Following data normalization, differential expression analysis was performed using the ShinyGo gene-set enrichment tool (30).

For quality control, barcode yields were evaluated to ensure adequate RNA capture with negative control probes defining background signal. Probes were removed globally from the dataset if either of the following was true: 1) the geometric mean of that probe's counts from all segments divided by the geometric mean of all probe counts representing the target from all segments is less than 0.1, or 2) the probe is an outlier according to the Grubb's test in at least 20% of the segments. Read depth per ROI, alignment rates, unique molecular identifier (UMI) counts, and gene detection above background were reviewed. Outlier ROIs were flagged and removed prior to downstream analysis. The Limit of Quantification (LOQ), defining the minimum expression level at which a transcript can be reliably and confidently distinguished from background noise in each ROI was determined and segments with exceptionally low signal were filtered out.

$$LOQ_i = \left(\prod_{i=1}^{n} NegProbe_i\right)^{1/n} \left(\exp\left(\sqrt{\frac{1}{n}\sum_{i=1}^{n}(\ln NegProbe_i - ln\ \overline{NegProbe_i})}\right) \right)$$

, where n is the total number of probes. After quality control review, 81 samples (ROIs) x 1304 genes passed. Finally, dimensionality reduction (PCA) and normalization techniques (Q3 normalization) were used to identify potential outliers, and none were observed.

Proximity Ligation Assay

Proximity ligation assays (PLAs) were performed as previously described (6). In brief, human tumor tissues were deparaffinized, rehydrated, subjected to antigen retrieval, permeabilized in 0.4% Triton-X in TBS for 20 min, and blocked using 0.1% BSA in TBS and 0.05% Tween solution for 30 minutes at room temperature. Slides were incubated overnight with human NLRP3 (aa 540-689) antibody (1:200, 0.2 mg/mL, R&D Systems, AF7010) and anti-human ASC mouse monoclonal antibody (1:200, 0.2 mg/mL, Santa Cruz Biotechnology, sc-514414) at 4°C. A mixture of 1x DuoLink in situ PLA probe anti-mouse PLUS and 1× DuoLink PLA probe anti-goat MINUS (Sigma-Aldrich, DU092001-30RXN) was added to the section and incubated for 1 hour at 37°C. Ligase solution was added to each sample for an additional 30 minutes followed by an amplification solution (35 μl) and incubated for 100 minutes. Finally, mounting medium with 4',6-diamidino-2-phenylindole (DAPI) was added and a cover slip was placed. Images were captured using a SP5 Leica confocal microscope. ImageJ software was used to quantify fluorescent dots in a blinded fashion based on three fields per tissue section at 40X (averaged over two tissue sections per tissue sample). The number of PLA dots quantified for a particular patient cohort was utilized to designate a tumor has having 'low' or 'high' PLA activity if the PLA quantification was below or above the median value, respectively.

Fluorescence In Situ Hybridization
FFPE tissue slides were deparaffinized and pre-treated with citric acid buffer, then digested with pepsin. After washing, the tissue slides were denatured and hybridized with a *NLRP3* FISH probe (Empire Genomics, NLRP3-10-GR) at 75°C for 10 minutes and 37°C for 16 hours and then counterstained with DAPI. A centromere probe for chromosome 1 (CEP1) served as an internal control (Empire Genomics, 1Q-10-O-A). The slides were examined under an SP8 Leica Confocal Microscope and the NLRP3:CEP1 amplification ratio was quantified using the LASX suite in a blinded fashion. NLRP3 and CEP1 ratios were quantified using established guidelines for the enumeration of HER2 amplification (49). The total number of NLRP3 counts were divided by the total number of CEP1 counts in at least 20 non-overlapping nuclei.

RNAseq and Tumor-intrinsic NLRP3 Transcriptional Signature Analysis
Flash-frozen tumors were placed in M-tubes (Miltenyi, 130-093-236) with RLT PLUS (QIAGEN, 1053393) containing β-mercaptoethanol (VWR, VWRV0482-250ML) and homogenized using the gentleMACS automated homogenization system. RNA was extracted from the tumors with the RNeasy Micro Kit (Qiagen, 74004). Messenger RNA was isolated from total RNA using poly-T oligo-attached magnetic beads. Subsequently, cDNA was synthesized. The library was prepared with the NEBNext Ultra II RNA Library Prep Kit. Quantified libraries were pooled and sequenced on the Illumina NovaSeq 6000 system based on their effective concentration and data requirements. Each sample yielded twenty million reads at PE150 depth.

Based on the bulk RNA-seq data from the whole tumor, common elements of the gene set were identified between the most upregulated genes in *Nlrp3*-amplified tumors and the most downregulated genes in *Nlrp3*-silenced tumors. This gene set was cross-referenced with detectable transcripts in human melanoma tissues based on the TCGA in order to generate a 48-transcript NLRP3-tsig signature. Publicly available RNA-seq datasets were filtered for samples with baseline tissue specimens and restricted to only naive stage IV melanoma patients receiving anti-PD-1 monotherapy. The raw RNA-seq data from the patient's samples was initially normalized by converting it to transcripts per million. Next, the ssGSEA score was computed for each sample using the generated NLRP3-tsig signature. Finally, a ROC curve was created based on these ssGSEA scores and the binary treatment response outcomes (R or NR). Patient were considered responders (R) if they demonstrated complete response, partial response, or stable disease, while they were considered non-responders (NR) if they demonstrated progressive disease.

TCGA Data Analysis
The TCGA data were accessed and visualized through cBioPortal and GEPIA2 (16, 50).

Supplemental Figures

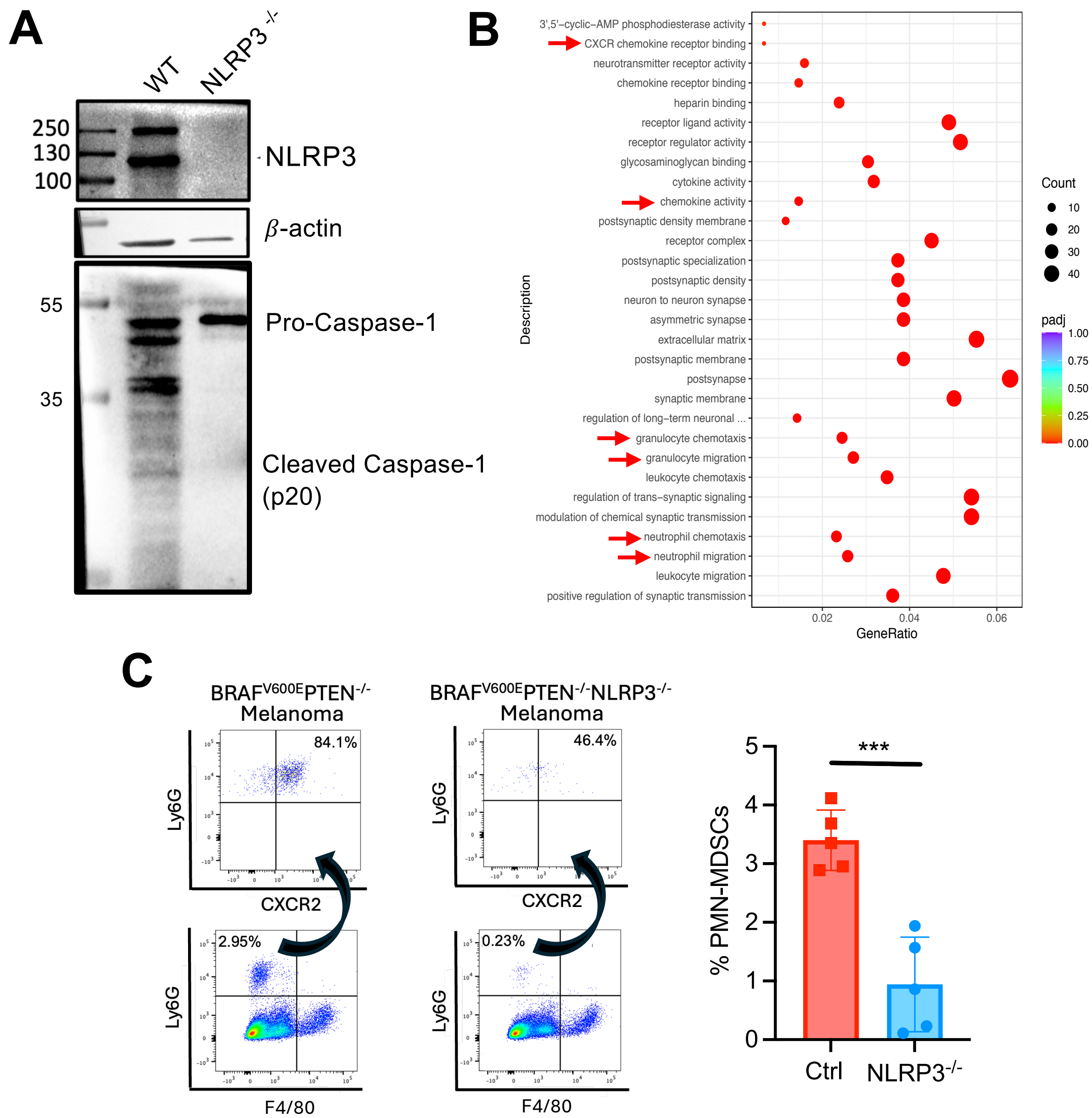

Supplemental Figure. 1. Characterization of Melanoma Tissues in the Transgenic Tyr-cre;BRAF^{V600E}PTEN$^{f/f}$NLRP3$^{f/f}$ Model. (A) NLRP3, pro-caspase-1, and cleaved caspase-1 (p20) Western blot analysis of BRAF^{V600E}PTEN$^{-/-}$NLRP3$^{-/-}$ and control BRAF^{V600E}PTEN$^{-/-}$ melanoma tissues (n = 2). **(B)** Most significant downregulated gene sets in BRAF^{V600E}PTEN$^{-/-}$NLRP3$^{-/-}$ melanoma tissues relative to control BRAF^{V600E}PTEN$^{-/-}$ melanoma tissues based on RNAseq. Red arrows, neutrophil and CXCR2-associated gene expression. GeneRatio, number of detected transcripts associated with a GO term divided by the total number of transcripts associated with the GO term. **(C)** PMN-MDSC quantification and measurement of CXCR2 expression by viable CD45^{+}CD11b^{+}Ly6G^{+}F4/80^{-} PMN-MDSCs in control BRAF^{V600E}PTEN$^{-/-}$ and BRAF^{V600E}PTEN$^{-/-}$NLRP3$^{-/-}$ melanoma tissues by flow cytometry. Statistical analysis performed using unpaired *t* tests (n = 5). Data expressed as mean values ± SEM. ****P*<0.0005.

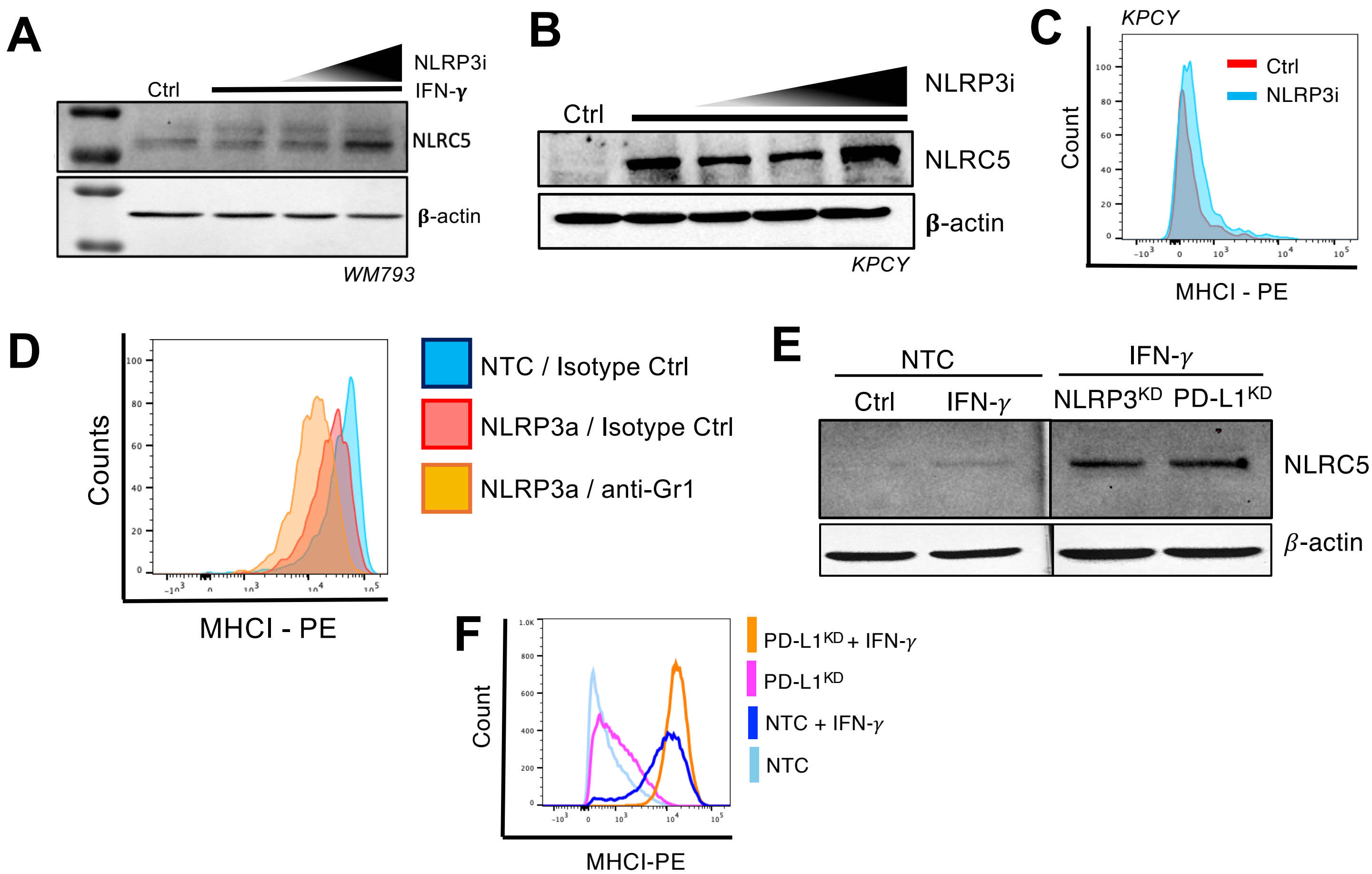

Supplemental Figure 2. Pharmacologic and Genetic Inhibition of the NLRP3 Signaling Pathway Enhances NLRC5 and MHC Class I Expression. (A) NLRC5 Western blot analysis of the human WM793 melanoma cell line following PBS or IFN-**γ** treatment with increasing concentrations of the NLRP3 inhibitor (NLRP3i), MCC950 (n = 3). **(B)** NLRC5 Western blot analysis of the KPCY pancreatic adenocarcinoma cell line following PBS or IFN-**γ** treatment with increasing concentrations of the NLRP3 inhibitor, MCC950 (n = 3). **(C)** pan-MHC class I flow cytometry analysis of the KPCY pancreatic adenocarcinoma cell line following treatment with a vehicle control versus MCC950 (n = 3). **(D)** Pan-MHC class I flow cytometry analysis of CD90.2$^-$CD31$^-$CD45$^-$ tumor cells derived from control BRAF^{V600E}-PTEN$^{-/-}$ melanoma and BRAF^{V600E}-PTEN$^{-/-}$NLRP3a melanoma cells following IgG isotype ctrl and anti-Gr-1 ab treatment. Elimination of tumor-infiltrating PMN-MDSCs does not reverse MHC class I downregulation. ab, antibody. (n = 2). **(E)** NLRC5 Western blot analysis of ctrl BRAF^{V600E}PTEN$^{-/-}$ cells, *Nlrp3*-silenced BRAF^{V600E}PTEN$^{-/-}$ cells, *Cd274 (PD-L1)*-silenced BRAF^{V600E}PTEN$^{-/-}$ cells. KD, knockdown (n = 3). **(F)** pan-MHC class I flow cytometry analysis of ctrl BRAF^{V600E}PTEN$^{-/-}$ cells and *Cd274 (PD-L1)*-silenced BRAF^{V600E}PTEN$^{-/-}$ cells following treatment with PBS versus IFN-**γ**. The 'NTC' and 'NTC + IFN-γ' controls were also utilized in Figure 5E. NTC, non-target control (n = 3).

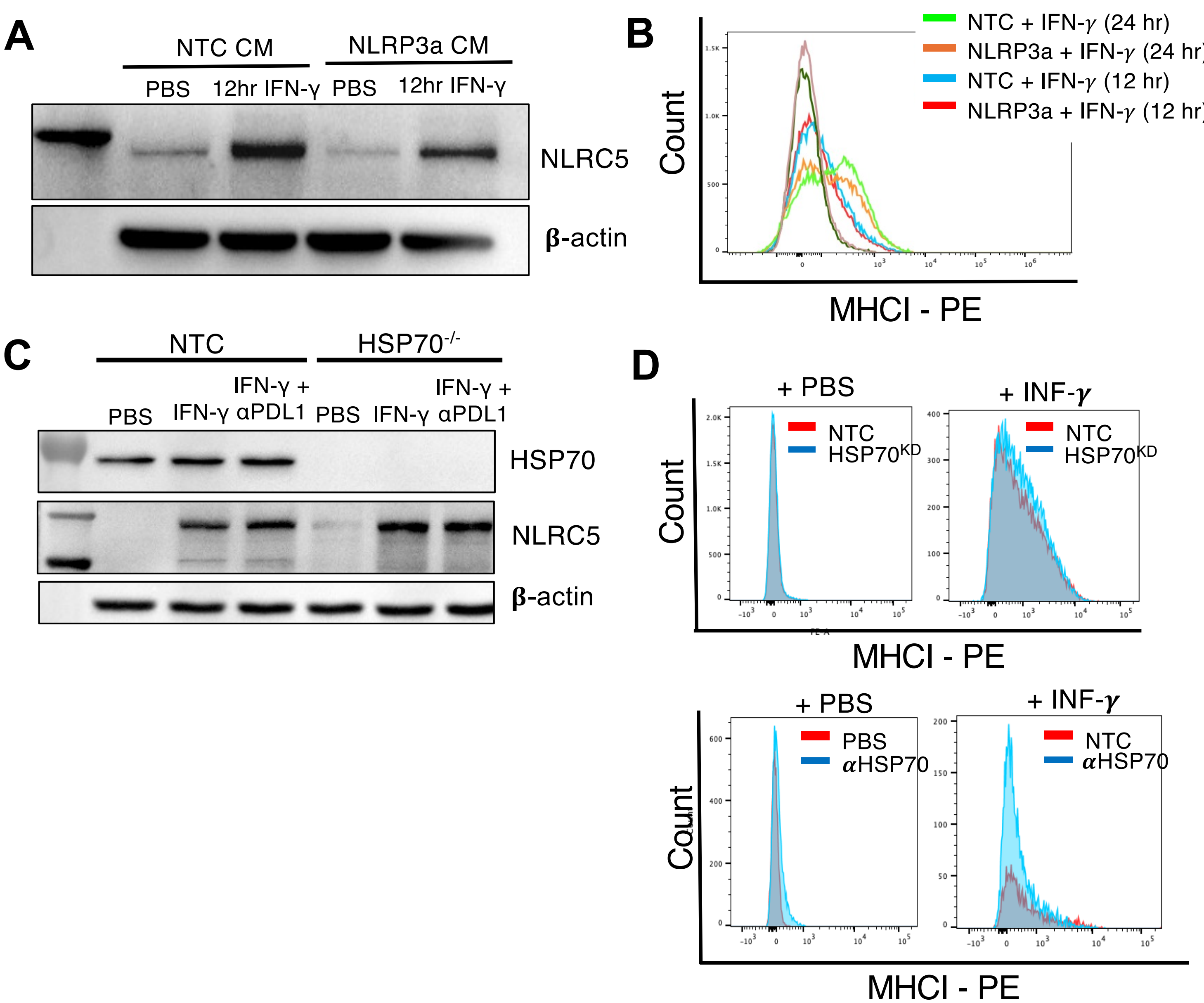

Supplemental Figure 3. HSP70 Modestly Suppresses Tumor NLRC5-MHC Class I Expression. **(A)** NLRC5 Western blot analysis of $BRAF^{V600E}PTEN^{-/-}$ melanoma cells following incubation in control $BRAF^{V600E}PTEN^{-/-}$ melanoma conditioned media (CM) versus $BRAF^{V600E}$-$PTEN^{-/-}$NLRP3a melanoma CM ± IFN-**γ** (n = 3). **(B)** pan-MHC class I flow cytometry analysis of $BRAF^{V600E}PTEN^{-/-}$ melanoma cells following incubation in control $BRAF^{V600E}PTEN^{-/-}$ melanoma CM versus $BRAF^{V600E}$-$PTEN^{-/-}$NLRP3a melanoma CM ± IFN-**γ** (n = 3). **(C)** HSP70 and NLRC5 Western blot analysis of $BRAF^{V600E}PTEN^{-/-}$ melanoma cells following incubation in control $BRAF^{V600E}PTEN^{-/-}$ melanoma CM versus $BRAF^{V600E}PTEN^{-/-}$ $HSP70^{-/-}$ CM plus treatment with PBS, IFN-**γ**, and IFN-**γ** + anti-PD-L1 ab (n = 3). **(D)** *Top,* pan-MHC class I flow cytometry analysis of control $BRAF^{V600E}PTEN^{-/-}$ melanoma cells versus *Hsp1a(Hsp70)*-silenced $BRAF^{V600E}PTEN^{-/-}$ melanoma cells ± IFN-**γ** (n = 3). *Bottom*, pan-MHC class I flow cytometry analysis of control $BRAF^{V600E}PTEN^{-/-}$ melanoma cells treated with anti-HSP70 ab ± IFN-**γ** (n = 3).

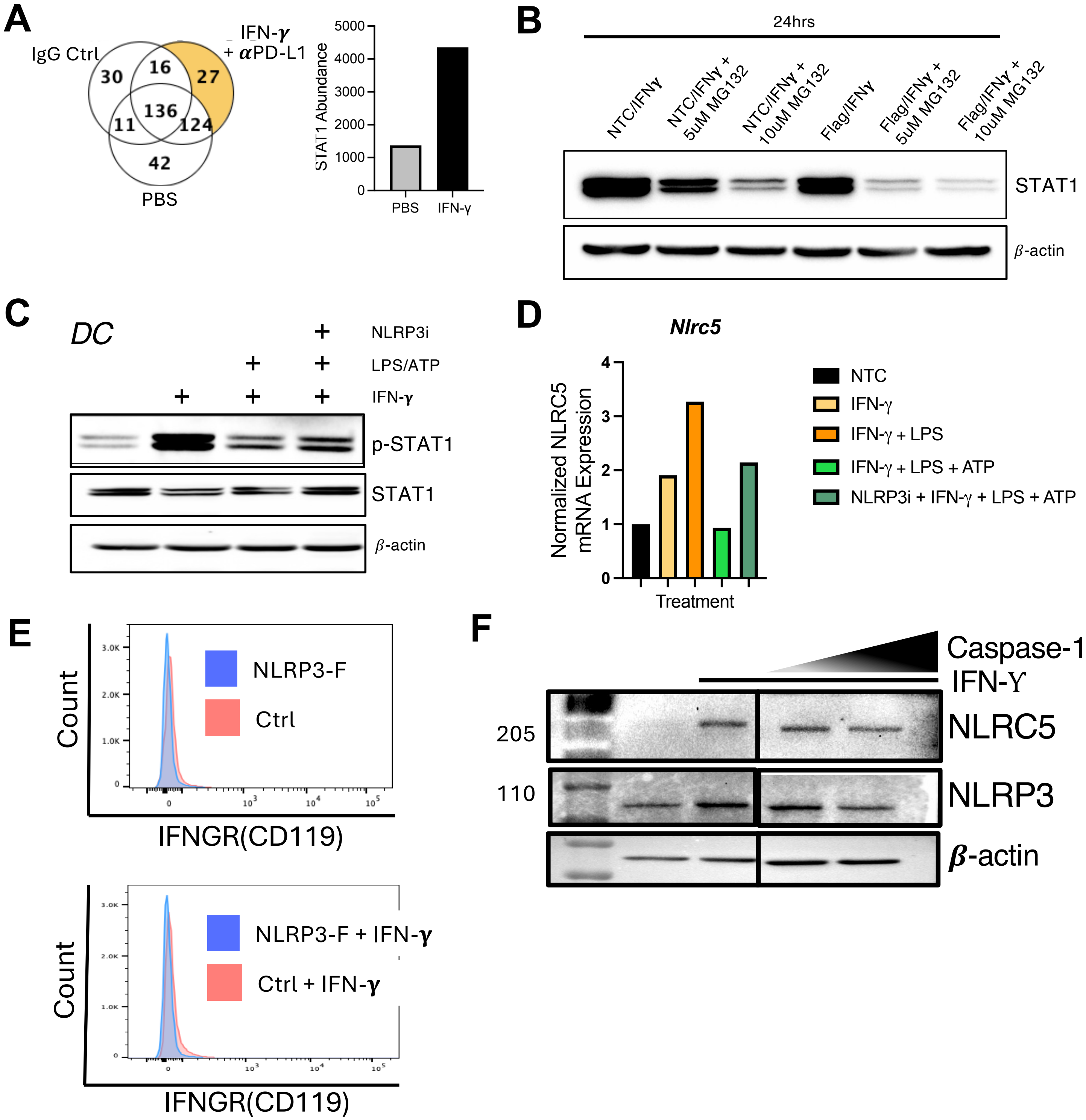
A
IgG Ctrl
IFN-γ + αPD-L1
30
16
27
136
11
124
42
PBS
STAT1 Abundance
5000
4000
3000
2000
1000
0
PBS
IFN-γ
B
24hrs
NTC/IFNγ
NTC/IFNγ + 5uM MG132
NTC/IFNγ + 10uM MG132
Flag/IFNγ
Flag/IFNγ + 5uM MG132
Flag/IFNγ + 10uM MG132
STAT1
β-actin
C
DC
NLRP3i
LPS/ATP
IFN-γ
p-STAT1
STAT1
β-actin
D
Nlrc5
Normalized NLRC5 mRNA Expression
Treatment
NTC
IFN-γ
IFN-γ + LPS
IFN-γ + LPS + ATP
NLRP3i + IFN-γ + LPS + ATP
E
Count
NLRP3-F
Ctrl
IFNGR(CD119)
Count
NLRP3-F + IFN-γ
Ctrl + IFN-γ
IFNGR(CD119)
F
Caspase-1i
IFN-γ
205
NLRC5
110
NLRP3
β-actin

Supplementary Figure 4. **(A)** *Left,* Venn diagram showing 27 unique proteins binding to NLRP3 as determined by tandem mass spectrometry following IFN-**γ**/anti-PD-L1 ab treatment. *Right,* IFN-**γ** treatment induced STAT1-NLRP3 binding based on tandem mass spectrometry (n = 2). **(B)** STAT1 Western blot analysis of control $BRAF^{V600E}PTEN^{-/-}$ melanoma cells and NLRP3-FLAG overexpressing $BRAF^{V600E}PTEN^{-/-}$ melanoma cells following treatment with IFN-**γ** and 5 or 10 mM of MG132. MG132, proteasomal inhibitor (n = 3). **(C)** p-STAT1 and t-STAT1 Western blot analysis of bone marrow-derived dendritic cells (DCs) following IFN-**γ**, IFN-**γ** + LPS/ATP, and IFN-**γ** + LPS/ATP + NLRP3i treatment (n = 3). **(D)** NLRC5 qrt-PCR analysis of bone marrow-derived DCs following IFN-**γ**, IFN-**γ** + LPS, IFN-**γ** + LPS/ATP, and IFN-**γ** + LPS/ATP + NLRP3i treatment (n = 3). **(E)** IFN-**γ** receptor (CD116) flow cytometry analysis of control (ctrl) and NLRP3-FLAG (NLRP3-F) overexpressing $BRAF^{V600E}PTEN^{-/-}$ melanoma cells ± IFN-**γ** (n = 3). **(F)** NLRC5 and NLRP3 Western blot analysis of $BRAF^{V600E}PTEN^{-/-}$ melanoma cells ± IFN-**γ** with increasing concentrations of caspase-1 inhibitor (n = 3).

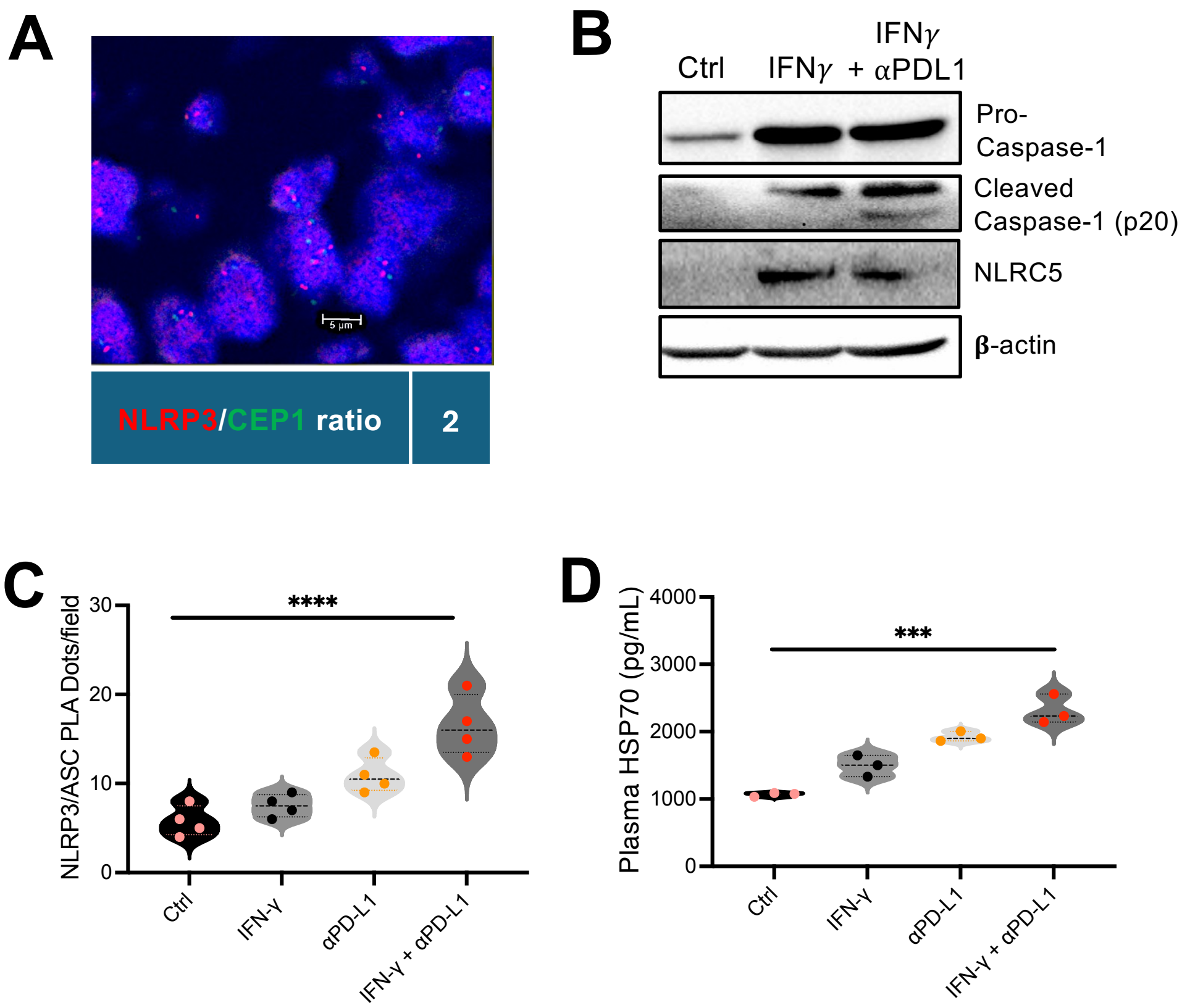

Supplementary Figure 5. Characterization of the CIN KPS Gastric Cancer Cell Line. (A) *NLRP3* FISH analysis of the KPS CIN gastric cancer cell line. Representative image shown. Scale bar = 5 μm. **(B)** Western blot analysis of pro-caspase-1, caspase-1, and NLRC5 expression levels in the KPS CIN gastric cancer cell line following treatment with PBS, IFN-γ, or IFN-γ + anti-PD-L1 ab (n = 3). β-actin loading control also used in **Supplementary Figure 6A**. **(C)** NLRP3-ASC PLA of the KPS CIN gastric cancer cell line following treatment with PBS, IFN-γ, or IFN-γ + anti-PD-L1 ab (n = 4). **(D)** HSP70 ELISA of conditioned media collected from the KPS CIN gastric cancer cell line following treatment with PBS, IFN-γ, or IFN-γ + anti-PD-L1 ab (n = 3). Statistical analysis performed using a one-way ANOVA followed by Tukey's multiple comparison test. All data presented in violin plots showing median and quartiles. ***P<0.0005, ****P<0.00005.

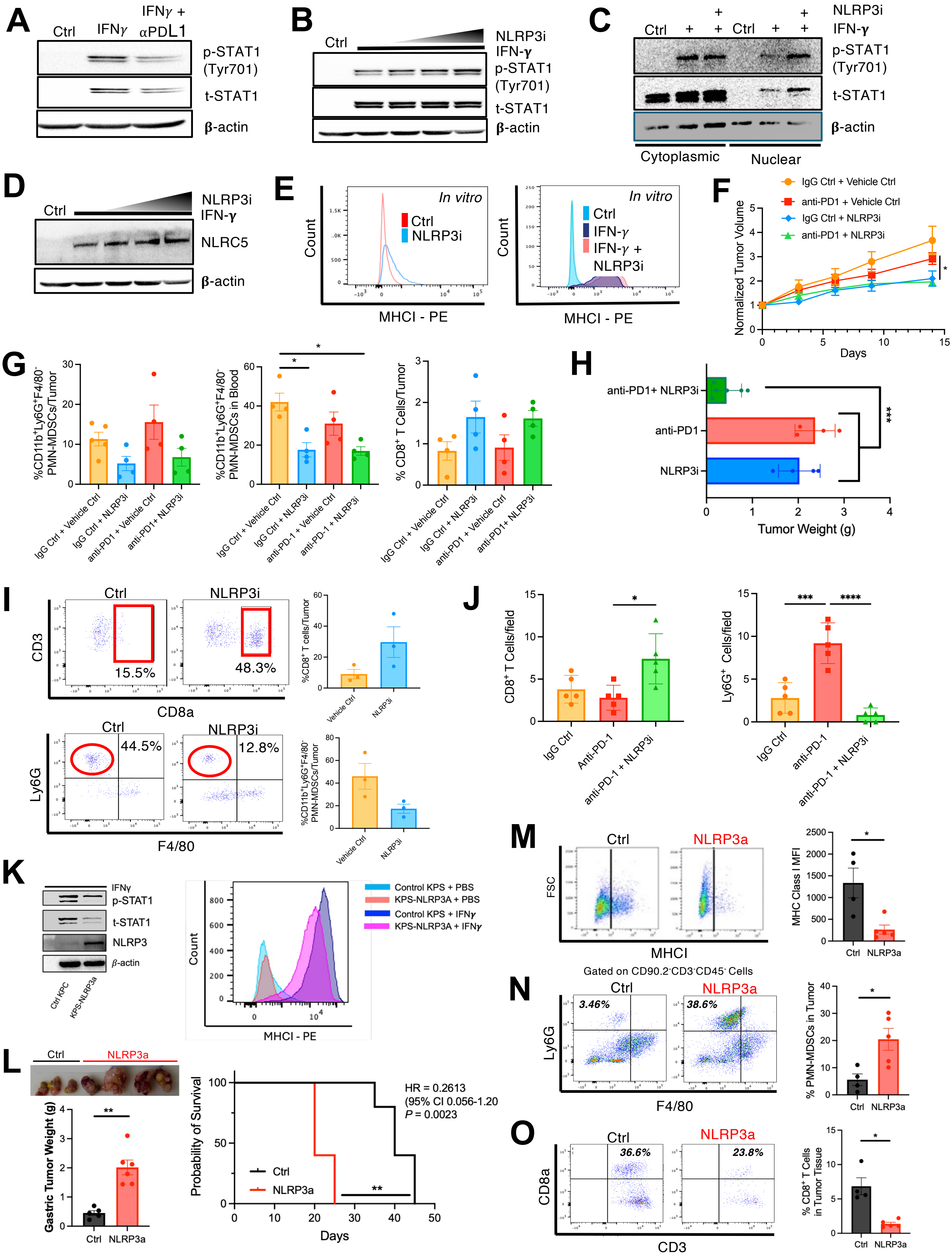
A
Ctrl
IFNγ
IFNγ + αPDL1
p-STAT1 (Tyr701)
t-STAT1
β-actin
B
Ctrl
NLRP3i
IFN-γ
p-STAT1 (Tyr701)
t-STAT1
β-actin
C
NLRP3i
IFN-γ
p-STAT1 (Tyr701)
t-STAT1
β-actin
Cytoplasmic
Nuclear
D
Ctrl
NLRP3i
IFN-γ
NLRC5
β-actin
E
In vitro
Ctrl
NLRP3i
IFN-γ
IFN-γ + NLRP3i
Count
MHCI - PE
F
IgG Ctrl + Vehicle Ctrl
anti-PD1 + Vehicle Ctrl
IgG Ctrl + NLRP3i
anti-PD1 + NLRP3i
Normalized Tumor Volume
Days
G
%CD11b+Ly6G+F4/80- PMN-MDSCs/Tumor
%CD11b+Ly6G+F4/80- PMN-MDSCs in Blood
% CD8+ T Cells/Tumor
IgG Ctrl + Vehicle Ctrl
IgG Ctrl + NLRP3i
anti-PD1 + Vehicle Ctrl
anti-PD1+ NLRP3i
H
anti-PD1+ NLRP3i
anti-PD1
NLRP3i
Tumor Weight (g)
I
Ctrl
NLRP3i
CD3
CD8a
15.5%
48.3%
Ly6G
F4/80
44.5%
12.8%
%CD8+ T cells/Tumor
%CD11b+Ly6G+F4/80- PMN-MDSCs/Tumor
Vehicle Ctrl
J
CD8+ T Cells/field
Ly6G+ Cells/field
IgG Ctrl
Anti-PD-1
anti-PD-1 + NLRP3i
K
IFNγ
p-STAT1
t-STAT1
NLRP3
β-actin
Ctrl KPC
KPS-NLRP3a
Control KPS + PBS
KPS-NLRP3A + PBS
Control KPS + IFNγ
KPS-NLRP3A + IFNγ
Count
MHCI - PE
L
Ctrl
NLRP3a
Gastric Tumor Weight (g)
Probability of Survival
HR = 0.2613
(95% CI 0.056-1.20
P = 0.0023
Days
M
FSC
MHCI
MHC Class I MFI
N
Gated on CD90.2-CD3-CD45- Cells
3.46%
38.6%
Ly6G
F4/80
% PMN-MDSCs in Tumor
O
36.6%
23.8%
CD8a
CD3
% CD8+ T Cells in Tumor Tissue

Supplementary Figure 6. NLRP3 Inhibition Enhances STAT1/MHC Class I Expression Levels, Augments Tumor CD8$^+$ T Cell Infiltration, and Suppresses Tumor Progression in the KPS CIN Gastric Adenocarcinoma Model. (**A)** p-STAT1 and t-STAT1 Western blot analysis of the KPS CIN gastric cancer cell line following treatment with PBS, IFN-γ, and IFN-γ/anti-PD-L1 ab (n = 3). β-actin loading control also used in **Supplementary Figure 5B**. **(B)** p-STAT and t-STAT1 Western blot analysis of the KPS CIN gastric cancer cell line following treatment with PBS, IFN-γ, increasing concentrations of NLRP3i (n = 3). β-actin loading control also used in **D**. **(C)** Nuclear and cytoplasmic p-STAT1 and t-STAT1 Western blot analysis of the KPS CIN gastric cancer cell line following treatment with PBS/DMSO, IFN-γ/DMSO, and IFN-γ/NLRP3i (n = 3). **(D)** NLRC5 Western blot analysis of the KPS CIN gastric cancer cell line following treatment with IFN-γ/NLRP3i (n = 3). β-actin loading control also used in **B**. **(E)** pan-MHC class I flow cytometry analysis of the KPS CIN gastric cancer cell line following treatment with NLRP3i ± IFN-γ (n = 3). **(F)** Tumor volume measurements of the syngeneic KPS CIN gastric cancer model during treatment with IgG isotype ctrl + vehicle ctrl, anti-PD-1 ab + vehicle ctrl, IgG isotype ctrl + NLRP3i, and anti-PD-1 ab + NLRP3i (n = 5). All data normalized to the control group. **(G)** *Left,* CD11b$^+$Ly6G$^+$F4/80$^-$ flow cytometry analysis of syngeneic KPS CIN gastric tumors; *Middle*, CD11b$^+$Ly6G$^+$F4/80$^-$ flow cytometry analysis of the peripheral blood of mice harboring syngeneic KPS CIN gastric tumors; *Right*, CD8$^+$ T cell flow cytometry analysis of syngeneic KPS CIN gastric tumors. (n = 5). **(H)** Tumor weight measurements (g) following treatment of the orthotopic KPS CIN gastric cancer model with NLRP3i + IgG isotype control, anti-PD-1 ab + vehicle ctrl, and anti-PD-1 ab + NLRP3i (n = 4). **(I)** *Top,* CD8$^+$ T cell flow cytometry analysis of orthotopic KPS CIN gastric tumors following treatment with vehicle control or NLRP3i. (n = 3). *Bottom*, CD11b$^+$Ly6G$^+$F4/80$^-$ flow cytometry analysis of orthotopic KPS CIN gastric tumors following treatment with vehicle control or NLRP3i. (n = 3). Representative data shown. **(J)** *Top,* CD8$^+$ T cell IHC analysis of orthotopic KPS CIN gastric tumors following treatment with vehicle control, anti-PD-1, or NLRP3i/anti-PD1 (n = 3). *Bottom*, CD11b$^+$Ly6G$^+$F4/80$^-$ flow cytometry analysis of orthotopic KPS CIN gastric tumors following treatment with vehicle control, anti-PD-1, or NLRP3i/anti-PD1 (n = 3). **(K)** *Left*, Total and phosphorylated STAT1 and NLRP3 Western blot analysis of a *Nlrp3*-amplified KPS CIN gastric cancer cell line (KPS-NLRP3a) versus a control KPS CIN gastric cancer cell line (Control KPS) treated with IFN-γ. *Right*, Pan-MHC class I flow cytometry analysis of a *Nlrp3*-amplified KPS CIN gastric cancer cell line versus a control KPS CIN gastric cancer cell line ± IFN-γ (n = 3). **(L)** *Left Top*, gross photos of control KPS gastric tumors vs KPS-NLRP3a gastric tumors. *Left Bottom*, control KPS gastric tumor vs KPS-NLRP3a gastric tumor weights. g, grams. *Right*, Survival analysis of mice harboring orthotopic control KPS gastric tumors or KPS-NLRP3a gastric tumors (n = 5). Statistical analysis based on log-rank test. **(M)** MHC class I flow cytometry analysis of CD90.2$^-$CD31$^-$CD45$^-$ tumor cells isolated from control KPS gastric tumors vs KPS-NLRP3a gastric tumors (n=4-5). Representative data shown. **(N)** CD11b$^+$Ly6G$^+$F4/80$^-$ PMN-MDSC flow cytometry analysis of control KPS gastric tumors vs KPS-NLRP3a gastric tumors (n=4-5). Representative data shown. **(O)** CD3$^+$CD8$^+$ T cell flow cytometry analysis of control KPS gastric tumors vs KPS-NLRP3a gastric tumors (n=4-5). Representative data shown. **F, G:** Statistical analysis performed using a two-way ANOVA followed by Tukey's multiple comparison test. **H, J:** Statistical analysis performed using a one-way ANOVA followed by Tukey's multiple comparison test. L – O: Statistical analysis performed using student's *t* test. All data expressed as mean values ± SEM. ns, non-significant. *P<0.05. **P<0.005. ***P<0.0005. ****P<0.00005.

A

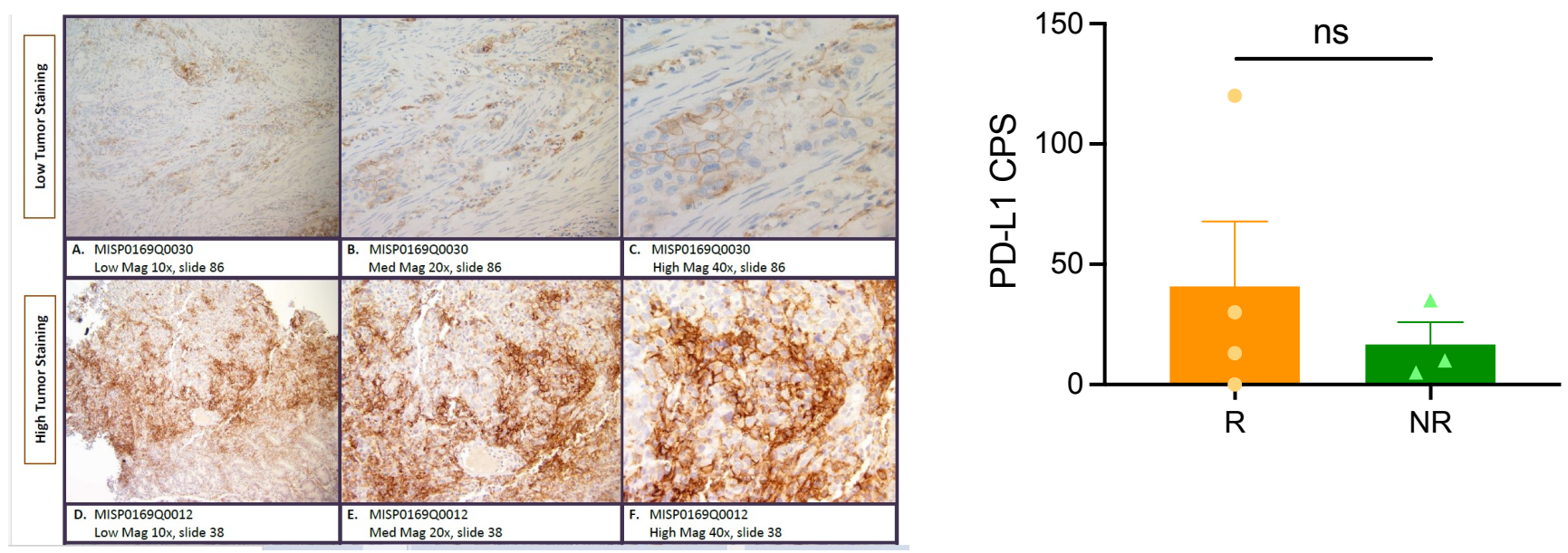

B

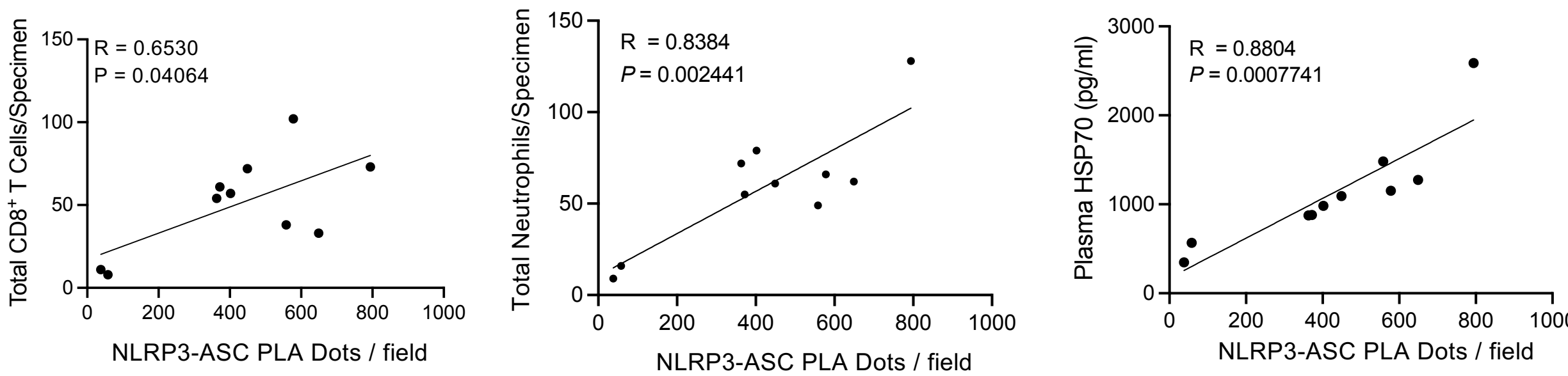

Supplementary Figure 7. PD-L1 Immunohistochemistry Does Not Correlate with Response to Pembrolizumab/CAPOX Therapy in KeyLARGO Advanced GE Adenocarcinoma Study. (A) *Left*, representative low and high PD-L1 IHC at 10x, 20x and 40x magnification images of GE adenocarcinoma tissues. *Right*, Available PD-L1 combined positive scores (CPS) of advanced gastroesophageal (GE) adenocarcinomas derived from responding (R) and non-responding (NR) patients (n=3-4). ns, non-significant. **(B)** NLRP3-ASC PLA correlation with tumor-infiltrating CD8$^+$ T cells and PMNs, as well as baseline plasma HSP70 levels in advanced GE adenocarcinoma patients. Statistical analysis performed using Spearman's rank correlation.

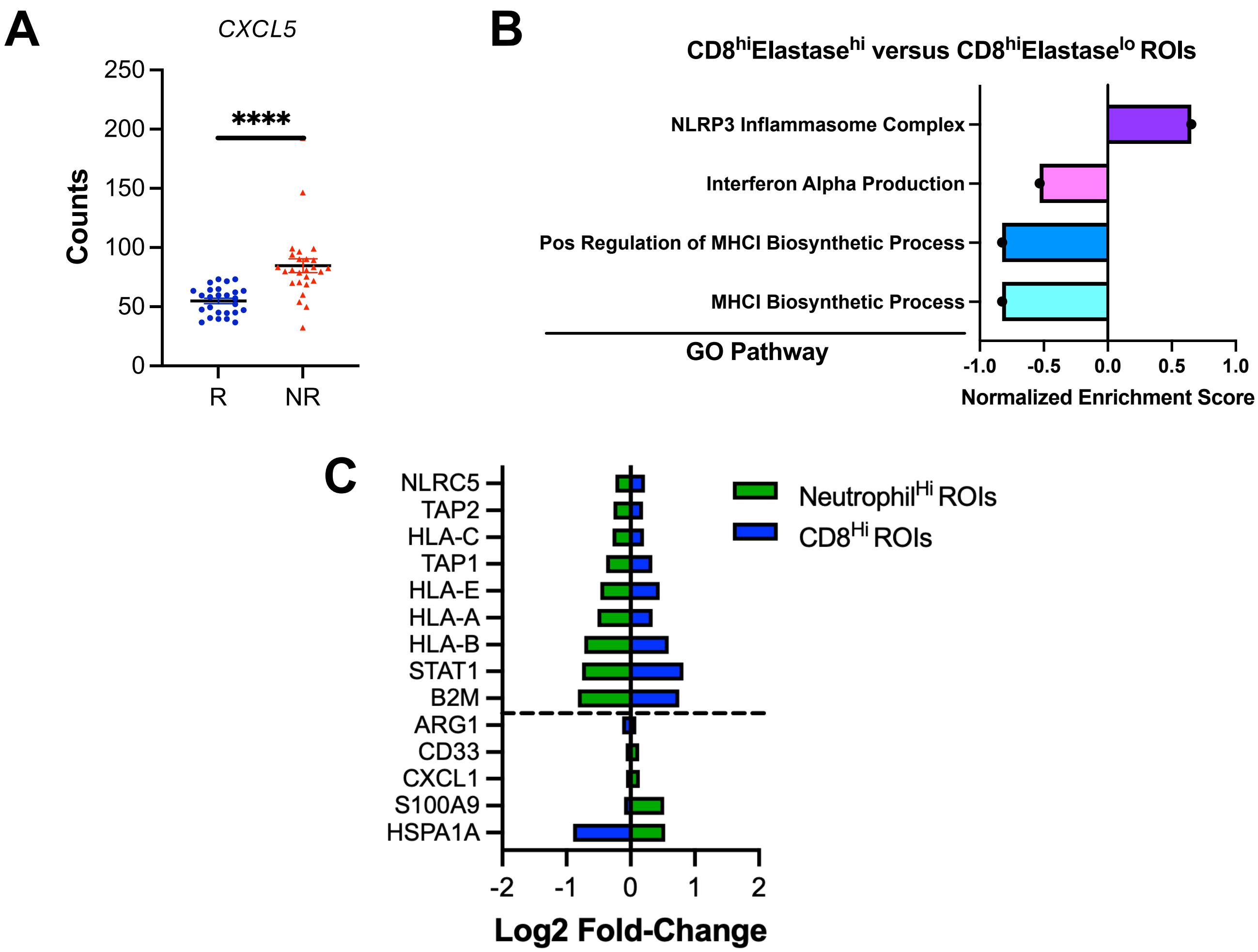

Supplementary Figure 8. Expression of NLRP3 Signaling Components Correlates with Enhanced PMNs in Patient-derived GE Adenocarcinoma Tissues. (A) RNAseq analysis of baseline tumor *CXCL5* expression in responding (R) and non-responding (NR) advanced GE adenocarcinoma patients treated with pembrolizumab/XELOX. Each dot represents a Region of Interest (ROI). **(B)** *Top*, GO Pathway Normalized Enrichment Scores based on Nanostring GeoMx spatial transcriptomic ROI analysis of GE adenocarcinoma tissues at baseline. $CD8^{hi}Elastase^{hi}$ versus $CD8^{hi}Elastase^{lo}$ ROIs selected based on IF analysis. Elastase, neutrophil/PMN marker. hi, above median of marker in ROI. lo, below median of marker in ROI. **(C)** Analysis of individual transcripts associated with MHC class I antigen processing and presentation and PMNs in individual $CD8^{+}$ T cell-rich ($CD8^{hi}$) and PMN-rich ($Elastase^{hi}$) ROIs. *HSPA1A*, encodes HSP70.

Supplementary Table 1.

Patient Number	Subject ID	Age (Decade)	Gender	Treatment	Stage	# of Involved Organs	BRAF Mutation	Objective Response	PFS (Months)	OS (Months)
1	H071	50s	F	anti-CTLA-4/anti-PD-1	M1b	3	WT	CR	60.0	60.0
2	H042	70s	M	anti-PD-1	M1c	2	WT	CR	60.0	60.0
3	V001	-	-	anti-PD-1	-	-	-	CR	60.0	60.0
4	V002	-	-	anti-PD-1	-	-	-	CR	60.0	60.0
5	V003	-	-	anti-CTLA-4	-	-	-	CR	60.0	60.0
6	H097	70s	F	anti-CTLA-4/anti-PD-1	M1b	4	WT	PR	6.1	17.7
7	H068	50s	M	anti-PD-1	M1a	2	WT	PR	60.0	60.0
8	H031	70s	M	anti-PD-1	M1a	1	WT	PR	60.0	60.0
9	H048	80s	F	anti-PD-1	M1a	1	WT	PR	60.0	60.0
10	H075	70s	M	anti-PD-1	M1b	3	WT	PR	60.0	60.0
11	H081	60s	F	anti-CTLA-4/anti-PD-1	M1a	2	WT	PR	60.0	60.0
12	H035	60s	M	anti-PD-1	M1c	1	WT	PR	60.0	60.0
13	H103	30s	M	anti-CTLA-4/anti-PD-1	M1d	2	WT	PR	60.0	60.0
14	H096	70s	M	anti-CTLA-4/anti-PD-1	M1d	6	WT	PR	60.0	60.0
15	H030	60s	M	anti-CTLA-4/anti-PD-1	M1c	4	MT	PR	60.0	60.0
16	H055	70s	M	anti-PD-1	M1a	1	WT	PR	60.0	60.0
17	H037	60s	M	anti-CTLA-4/anti-PD-1	M1b	2	WT	PR	60.0	60.0
18	H053	50s	F	anti-PD-1	M1b	2	WT	PR	60.0	60.0
19	H058	80s	M	anti-PD-1	M1c	4	WT	PR	22.7	33.3
20	H065	50s	M	anti-CTLA-4/anti-PD-1	M1c	2	MT	PR	60.0	60.0
21	H098	60s	M	anti-PD-1	M1a	2	WT	PR	60.0	60.0
22	H107	60s	F	anti-CTLA-4/anti-PD-1	M1a	3	WT	PR	60.0	60.0
23	H110	40s	M	anti-CTLA-4/anti-PD-1	M1c	1	WT	PR	11.6	60.0
24	V012	-	-	anti-PD-1	-	-	-	PR	60.0	60.0
25	H087	20s	F	anti-PD-1	M1a	1	MT	SD	2.1	29.0
26	H052	80s	M	anti-PD-1	M1c	2	WT	SD	60.0	60.0
27	V013	-	-	anti-PD-1	-	-	-	SD	14.2	52.8
28	H109	30s	M	anti-CTLA-4/anti-PD-1	M1d	3	MT	PD	1.6	60.0
29	H105	60s	F	anti-CTLA-4/anti-PD-1	M1d	7	MT	PD	17.0	60.0
30	H061	40s	F	anti-PD-1	M1a	2	MT	PD	2.0	60.0
31	H049	70s	M	anti-CTLA-4/anti-PD-1	M1a	1	WT	PD	2.7	12.3
32	H073	60s	M	anti-CTLA-4/anti-PD-1	M1b	1	WT	PD	3.0	13.4
33	H045	30s	M	anti-PD-1	M1a	1	MT	PD	26.8	60.0
34	H054	40s	F	anti-CTLA-4/anti-PD-1	M1c	3	MT	PD	0.7	23.4

Supplementary Table 1 (cont).

Patient Number	Subject ID	Age (Decade)	Gender	Treatment	Stage	# of Involved Organs	BRAF Mutation	Objective Response	PFS (Months)	OS (Months)
35	H059	60s	F	anti-PD-1	M1a	1	WT	SD	60.0	60.0
36	H093	80s	F	anti-PD-1	M1a	3	WT	PD	8.3	60.0
37	H129	60s	F	anti-CTLA-4/anti-PD-1	M1a	1	MT	SD	60.0	60.0
38	V004	-	-	anti-PD-1	-	-	-	PD	7	18
39	V005	-	-	anti-PD-1	-	-	-	PD	2.8	9.8
40	V006	-	-	anti-PD-1	-	-	-	PD	2.1	2.4
41	V007	-	-	anti-PD-1	-	-	-	PD	0.88	2.2
42	V008	-	-	anti-PD-1	-	-	-	PD	2.7	5.7
43	V009	-	-	anti-PD-1	-	-	-	PD	2.8	9.8
44	V010	-	-	anti-PD-1	-	-	-	PD	3.2	7.5
45	V011	-	-	anti-CTLA4	-	-	-	PD	2.8	8.0
46	H039	70s	M	anti-PD-1	M1d	3	MT	PD	2.8	60.0
47	H032	40s	F	anti-PD-1	M1c	1	MT	PD	5.4	13.2
48	H102	70s	M	anti-CTLA-4/anti-PD-1	M1a	1	MT	PD	2.8	60.0
49	H043	40s	M	anti-PD-1	M1a	1	MT	PR	1.1	8.0
50	H057	50s	F	anti-CTLA-4/anti-PD-1	M1a	3	MT	PR	11.0	60.0
51	H076	50s	M	anti-PD-1	M1a	2	-	PR	60.0	60.0
52	H078	60s	F	anti-PD-1	M1c	2	WT	PR	6.0	29.0
53	H083	60s	F	anti-CTLA-4/anti-PD-1	M1a	2	WT	PR	60.0	60.0
54	H090	50s	M	anti-CTLA-4/anti-PD-1	M1d	2	-	PR	60.0	60.0
55	H104	70s	M	anti-PD-1	M1c	1	WT	PR	60.0	60.0
56	H112	70s	F	anti-CTLA-4/anti-PD-1	M1a	3	WT	PR	60.0	60.0
57	H113	60s	F	anti-PD-1	M1a	1	MT	PR	60.0	60.0
58	H115	70s	M	anti-PD-1	M1d	2	WT	PR	60.0	60.0
59	H079	80s	M	anti-PD-1	M1a	1	WT	SD	10.0	16.1
60	H066	60s	M	anti-PD-1	M1a	1	WT	SD	60.0	60.0
61	H034	80s	F	anti-PD-1	M1b	2	MT	PD	3.3	3.8
62	H060	60s	F	anti-PD-1	M1c	2	MT	SD	5.0	60.0

Supplementary Table 1. Stage IV Melanoma Patient Data. All patients treatment naïve. F, female. M, male. WT, BRAF wild type. MT, BRAF(V600E) mutant. CR, complete response. PR, partial response. SD, stable disease. PD, progressive disease. All responses based on RECIST1.1 criteria and 5 years of follow-up.

Supplementary Table 2

Covariate	Hazard Ratio	P value	95% CI
Anti-PD-1 Treatment (n = 46)	0.84	0.71	(0.35, 2.05)
**Low NLRP3 Activity (n = 46)**	**0.12**	**0.0007**	**(0.04, 0.41)**
Anti-PD-1 Treatment (n = 35)	1.14	0.83	(0.35, 3.64)
Gender (n = 35)	0.86	0.81	(0.24, 3.08)
Age (n = 35)	0.99	0.57	(0.94, 1.03)
M1b (n = 35)	1.12	0.93	(0.09, 13.26)
M1c (n = 35)	1.76	0.39	(0.48, 6.50)
M1d (n = 35)	1.77	0.64	(0.16, 19.47)
# of Involved Organs (n = 35)	0.98	0.92	(0.59, 1.61)
BRAF Mutation Status (n = 35)	2.89	0.16	(0.66, 12.57)
**Low NLRP3 Activity (n = 35)**	**0.13**	**0.02**	**(0.03, 0.69)**

Supplementary Table 2. Multivariate Analysis of Progression-Free Survival of Naïve Stage IV Melanoma Patients Undergoing Checkpoint Inhibitor Immunotherapy. Treatment refers either to anti-PD-1 monotherapy (reference) or anti-CTLA-4/anti-PD-1 combination therapy. Treatment and NLRP3 activity level available for all 46 patients (n = 46). Gender, Age, Staging, BRAF mutation status covariates available for 35 patients (n = 35). Statistical analysis based on Cox proportional hazards model.

Supplementary Table 3.

Patient Number	Subject ID	Gender	BRAF Mutation Status	Treatment	RFS (Months)
1	H064	F	WT	anti-PD-1	66.5
2	H067	M	WT	anti-PD-1	65.9
3	H074	M	MT	anti-PD-1	52.0
4	H086	M	WT	anti-PD-1	8.2
5	H091	F	WT	anti-PD-1	56.3
6	H092	F	WT	anti-PD-1	54.9
7	H099	M	WT	anti-PD-1	13.4
8	H101	M	MT	anti-PD-1	7.1
9	H106	M	WT	anti-PD-1	44.1
10	H114	F	WT	anti-PD-1	0.47
11	H116	M	WT	anti-PD-1	27.3
12	H117	M	WT	anti-PD-1	38.5
13	H118	M	WT	anti-PD-1	38.5

Supplementary Table 3. Stage III Melanoma Patient Data. All patients treated with adjuvant anti-PD-1 monotherapy. All patients monitored by CT/PET CT over 5 years of follow-up. F, female. M, male. WT, BRAF wild type. Mt, BRAF(V600E) mutant.

Supplementary Table 4.

Gene Number	Gene Name
1	LBP
2	RASL10B
3	STRA6
4	MATN4
5	SERPING1
6	KCNMA1
7	KCNS1
8	NOS2
9	HAS1
10	C3
11	SFRP5
12	CEBPD
13	PDE8B
14	TMEM108
15	HS3ST1
16	PDE1A
17	AEBP1
18	CHRDL1
19	SULF1
20	GPC3
21	PTGER3
22	SERPINE2
23	SRPX2
24	KCNK10
25	SOD3
26	THBS2
27	MMP19
28	HSD11B1
29	NTRK2
30	FGF7
31	GRIK2
32	CHRDL2
33	CLEC4D
34	INHBA
35	RTN4RL2
36	IL1B
37	APOD
38	MMP3
39	BDKRB1
40	CLEC4E
41	CLSTN2
42	LIF
43	RBPJL
44	NFASC
45	CXCL5
46	SLC35D3
47	SAA3P
48	IL6

Supplementary Table 4. NLRP3-tsig Transcriptional Signature. Signature generated by an unbiased integrated assessment of the transcriptional profile of *Nlrp3*-amplified $BRAF^{V600E}PTEN^{-/-}$ melanomas and *Nlrp3*$^{-/-}$ $BRAF^{V600E}PTEN^{-/-}$ melanomas.

Supplementary Table 5.

Protein Name	Accession
Signal transducer and activator of transcription 1 (STAT1)	splP42225lSTAT1_MOUSE
Antigen peptide transporter 2 (TAP2)	splP36371lTAP2_MOUSE
Neurofilament medium polypeptide (NFM)	splP08553lNFM_MOUSE
Interferon-induced transmembrane protein 3 (IFM3)	splQ9CQW9lIFM3_MOUSE
Calsyntenin-1 (CSTN1)	rev_splQ9EPL2lCSTN1_MOUSE
Ankycorbin (RAI14)	splQ9EP71lRAI14_MOUSE
Interferon-induced protein with tetratricopeptide repeats 1 (IFIT1)	splQ64282lIFIT1_MOUSE
Ras-related protein Rab-14 (RAB14)	splQ91V41lRAB14_MOUSE
F-actin-capping protein subunit alpha-2 (CAZA2)	splP47754lCAZA2_MOUSE
Immunity-related GTPase family M protein 1 (IRGM1)	splQ60766lIRGM1_MOUSE
GRB2-associated-binding protein 3 (GAB3)	rev_splQ8BSM5lGAB3_MOUSE
DnaJ homolog subfamily A member 1 (DNJA1)	splP63037lDNJA1_MOUSE
2'-5'-oligoadenylate synthase 1A (OAS1A)	splP11928lOAS1A_MOUSE
Probable ATP-dependent RNA helicase DDX6 (DDX6)	splP54823lDDX6_MOUSE
Leucine-rich repeat serine/threonine-protein kinase 2 (LRRK2)	splQ5S006lLRRK2_MOUSE
2'-5'-oligoadenylate synthase-like protein 1 (OASL1)	splQ8VI94lOASL1_MOUSE
Cytochrome c oxidase subunit 7A2, mitochondrial (CX7A2)	splP48771lCX7A2_MOUSE
Ceramide synthase 2 (CERS2)	splQ924Z4lCERS2_MOUSE
Transducin beta-like protein 2 (TBL2)	splQ9R099lTBL2_MOUSE
Torsin-1A-interacting protein 1 (TOIP1)	splQ921T2lTOIP1_MOUSE
Prolow-density lipoprotein receptor-related protein 1 (LRP1)	splQ91ZX7lLRP1_MOUSE
Vigilin (VIGLN)	splQ8VDJ3lVIGLN_MOUSE
T-complex protein 1 subunit beta (TCPB)	splP80314lTCPB_MOUSE
UDP-glucuronosyltransferase 1-6 (UD16)	splQ64435lUD16_MOUSE
G-protein coupled receptor-associated protein LMBRD2 (LMBD2)	splQ8C561lLMBD2_MOUSE
Histone-arginine methyltransferase CARM1 (CARM1)	splQ9WVG6lCARM1_MOUSE
Basement membrane-specific heparan sulfate proteoglycan core protein (PGBM)	splQ05793lPGBM_MOUSE

Supplementary Table 5. NLRP3 Binding Proteins in Tumor Cells Following IFN-γ/anti-PD-L1 ab Activation. Binding proteins identified by tandem mass spectrometry performed in duplicate.

Supplementary Table 6.

Patient Number	Subject ID	Age (Decade)	Gender	Tumor Site	PD-L1 CPS	Objective Response	PFS (Months)	OS (Months)
1	111	60s	M	Esophagus	-	PR	35.6	37.3
2	129	70s	M	Esophagus	15	PR	13.1	24.3
3	114	50s	M	Esophagus	60	PR	12.2	22.9
4	125	30s	M	GE Junction	0	SD	6.6	31.4
5	141	60s	M	Esophagus	-	PR	2.1	2.1
6	118	60s	M	Esophagus	4	PD	2.4	2.8
7	127	60s	M	Esophagus	1	PD	2.0	3.0
8	139	60s	M	GE Junction	20	PD	2.3	9.2
9	140	40s	M	Esophagus	-	PD	2.3	11.6
10	121	40s	F	GE Junction	10	SD	4.6	15.8
11	120	60s	F	GE Junction	0	PD	1.8	3.5
12	101	60s	M	Esophagus	40	CR	42.1	44.6
13	103	40s	M	Stomach	15	PR	6.7	11.5
14	104	50s	M	Esophagus	3	PD	4.2	18.4
15	105	70s	M	GE Junction	25	CR	28.2	28.2
16	106	60s	M	Esophagus	2	PR	6.3	16.0
17	107	57	M	Esophagus	1	PR	11.2	40.9
18	108	50s	M	GE Junction	3	PR	8.51	8.51
19	109	50s	M	Esophagus	10	PR	35.1	39.1
20	112	50s	M	Esophagus	0	CR	24.5	35.3
21	117	50s	M	Esophagus	-	PR	7.6	7.6
22	119	30s	F	GE Junction	0	PR	9.7	30.9
23	122	70s	M	Esophagus	0	PR	23.0	31.3
24	123	40s	M	Stomach	-	PR	4.8	13.9
25	126	70s	M	GE Junction	1	PR	14.6	14.6
26	128	60s	M	Esophagus	-	PR	8.7	29.9
27	130	40s	F	Stomach	5	SD	5.8	8.5
28	132	60s	M	GE Junction	-	PR	6.6	11.6
29	133	70s	F	GE Junction	2	PD	2.5	2.7
30	134	60s	F	Stomach	0	SD	17.5	17.5
31	135	50s	M	GE Junction	20	PD	2.5	4.1
32	136	70s	M	GE Junction	20	PR	8.8	24.3
33	501	70s	M	GE Junction	-	PR	3.5	4.1
34	502	60s	M	Stomach	-	PR	7.1	17.7
35	503	30s	M	Stomach	-	PR	12.2	25.7

Supplementary Table 6. Advanced Gastroesophageal Adenocarcinoma Patient Data. All patients treatment naïve. F, female. M, male. GE, gastroesophageal. CPS, Combined Positive Score. CR, complete response. PR, partial response. SD, stable disease. PD, progressive disease. All responses based on RECIST1.1 criteria.

Supplementary Table 7.

Gene	Forward	Reverse
Stat1 (m)	TCACAGTGGTTCGAGCTTCA	GCAAACGAGACATCATAGGCA
β2m (m)	ACAGTTCCACCCGCCTCACATT	TAGAAAGACCAGTCCTTGCTGAAG
Nlrc5 (m)	TCAGCCCAGAACAAGTATCC	TGGGCACAGACTTCCATTAG
Actb (m)	GGCTGTATTCCCCTCCATCG	CCAGTTGGTAACAATGCCATGT

Supplementary Table 7. Qrt-PCR Primers. m, murine.
