# Supplementary material for: Tumor NLRP3 amplification promotes immunotherapy resistance by inhibiting STAT1 signaling and MHC class I expression
Source: J Clin Invest. 2026 Aug 13;136(20):e201816. doi: 10.1172/JCI201816 (PMC13668618; doi:10.1172/JCI201816)

Uncut Western Blots

Figure 3B

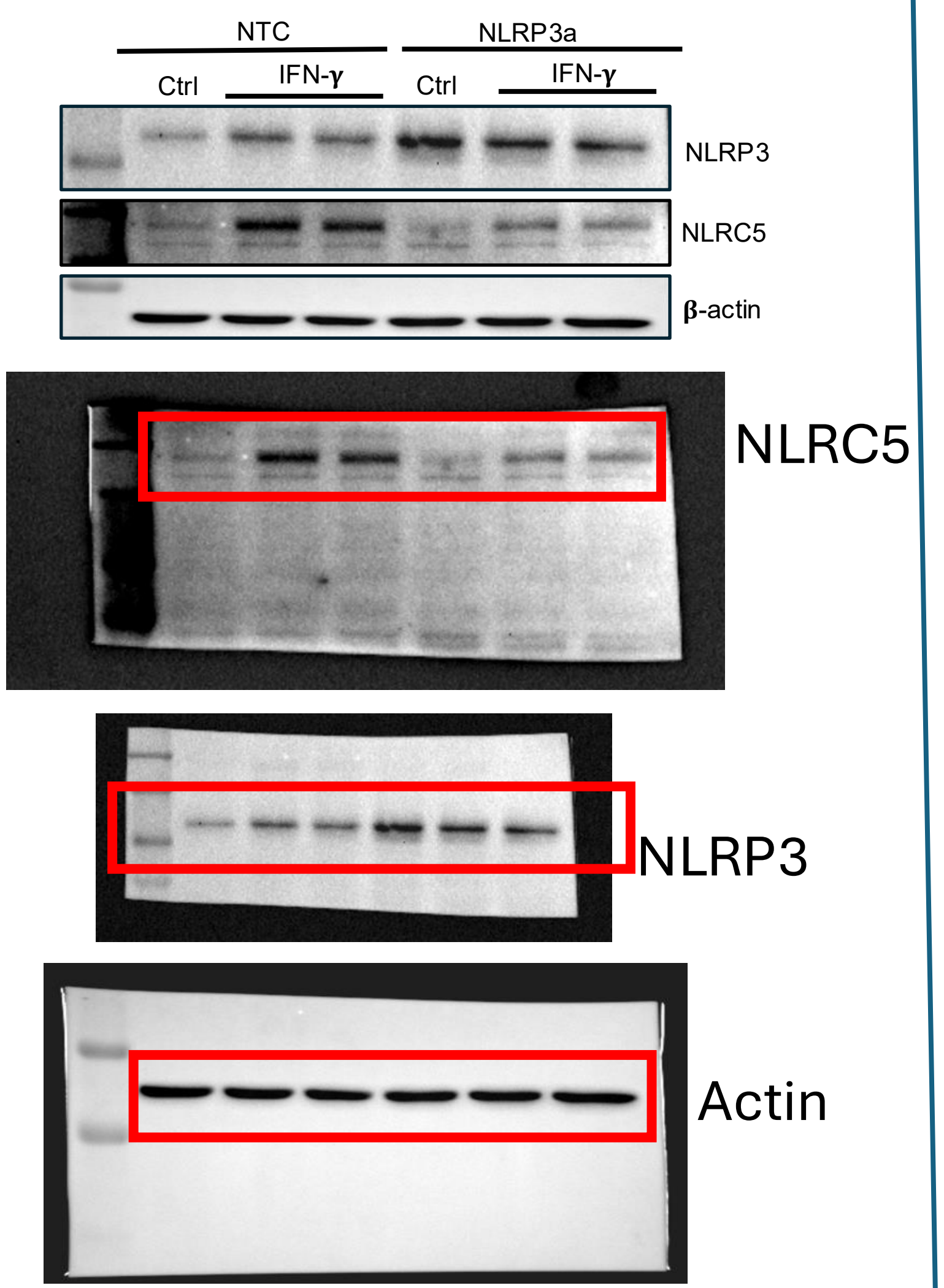

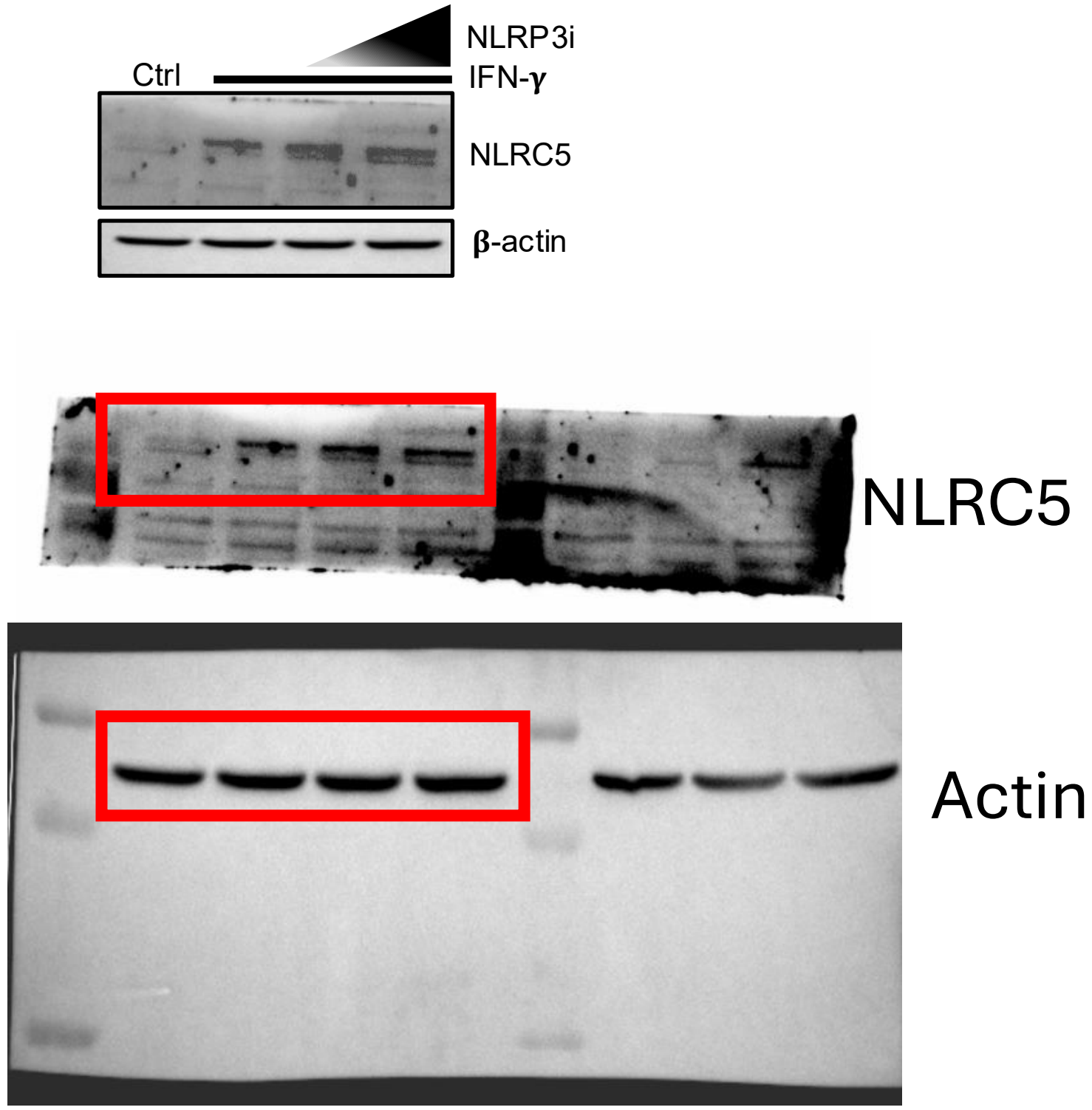

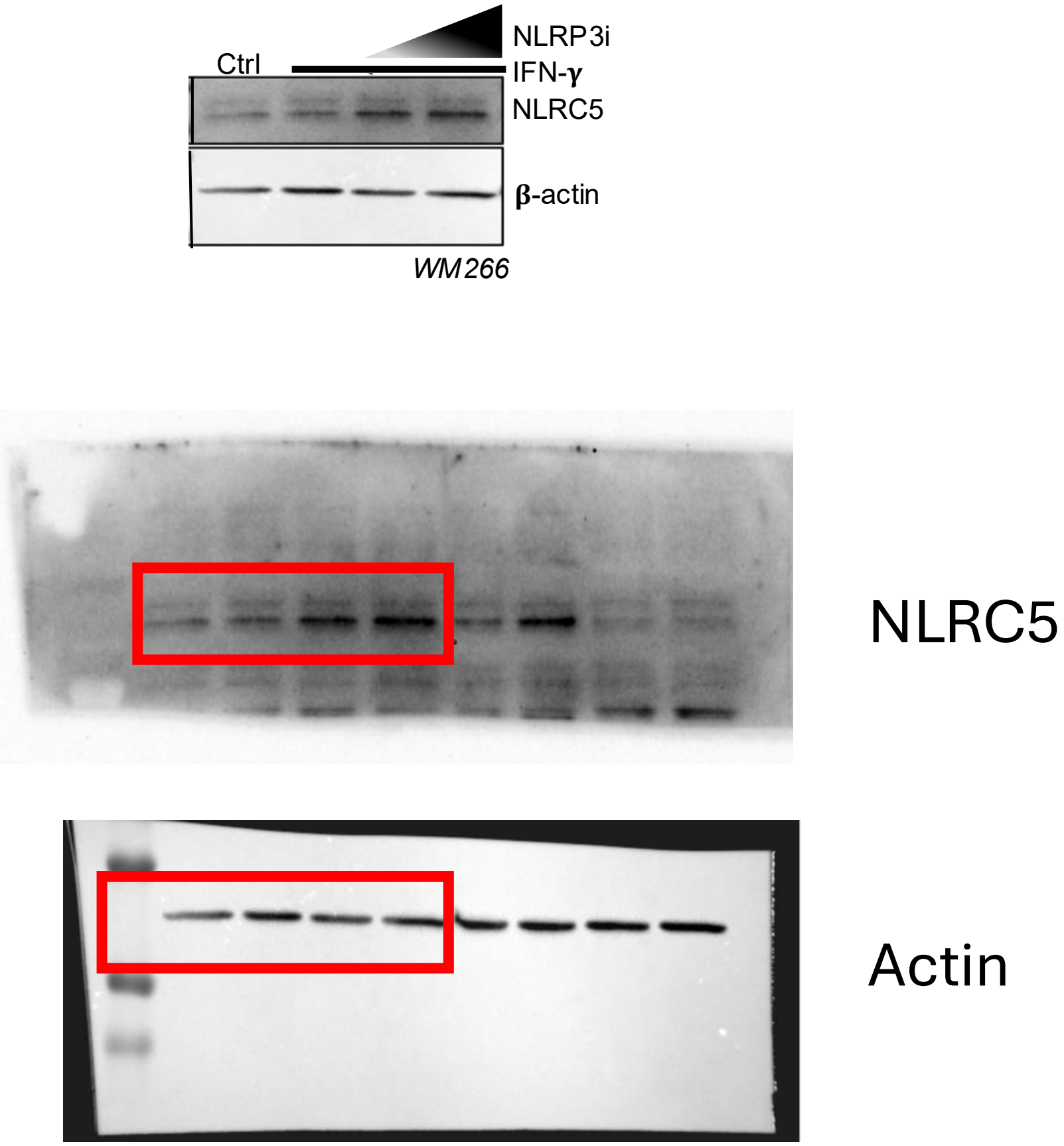

Figure 4

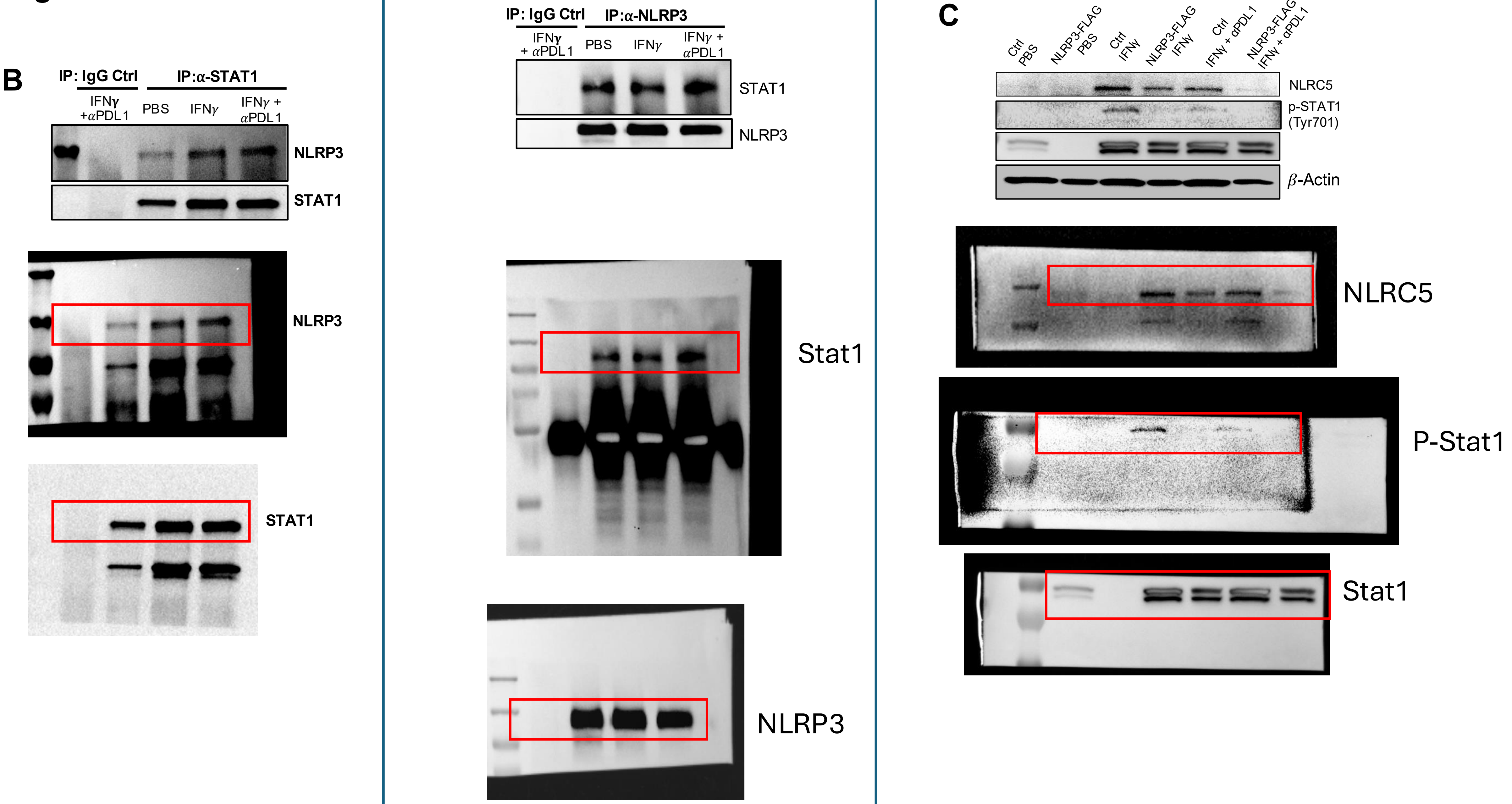
B
IP: IgG Ctrl
IP:α-STAT1
IFNγ +αPDL1
PBS
IFNγ
IFNγ + αPDL1
NLRP3
STAT1
NLRP3
STAT1
IP: IgG Ctrl
IP:α-NLRP3
IFNγ + αPDL1
PBS
IFNγ
IFNγ + αPDL1
STAT1
NLRP3
Stat1
NLRP3
C
Ctrl PBS
NLRP3-FLAG PBS
Ctrl IFNγ
NLRP3-FLAG IFNγ
Ctrl IFNγ + αPDL1
NLRP3-FLAG IFNγ + αPDL1
NLRC5
p-STAT1 (Tyr701)
β-Actin
NLRC5
P-Stat1
Stat1

Figure 4D

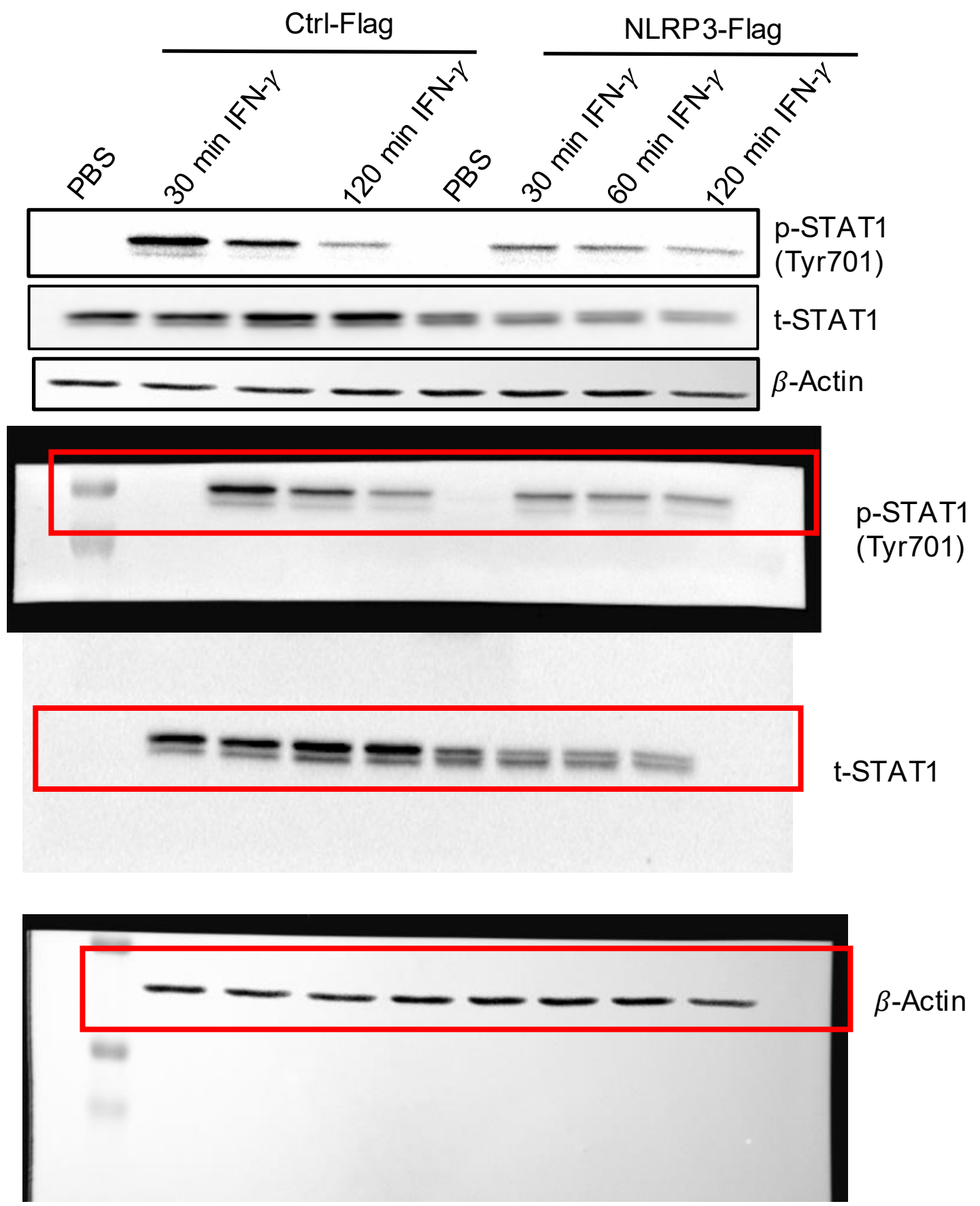

Figure 4E

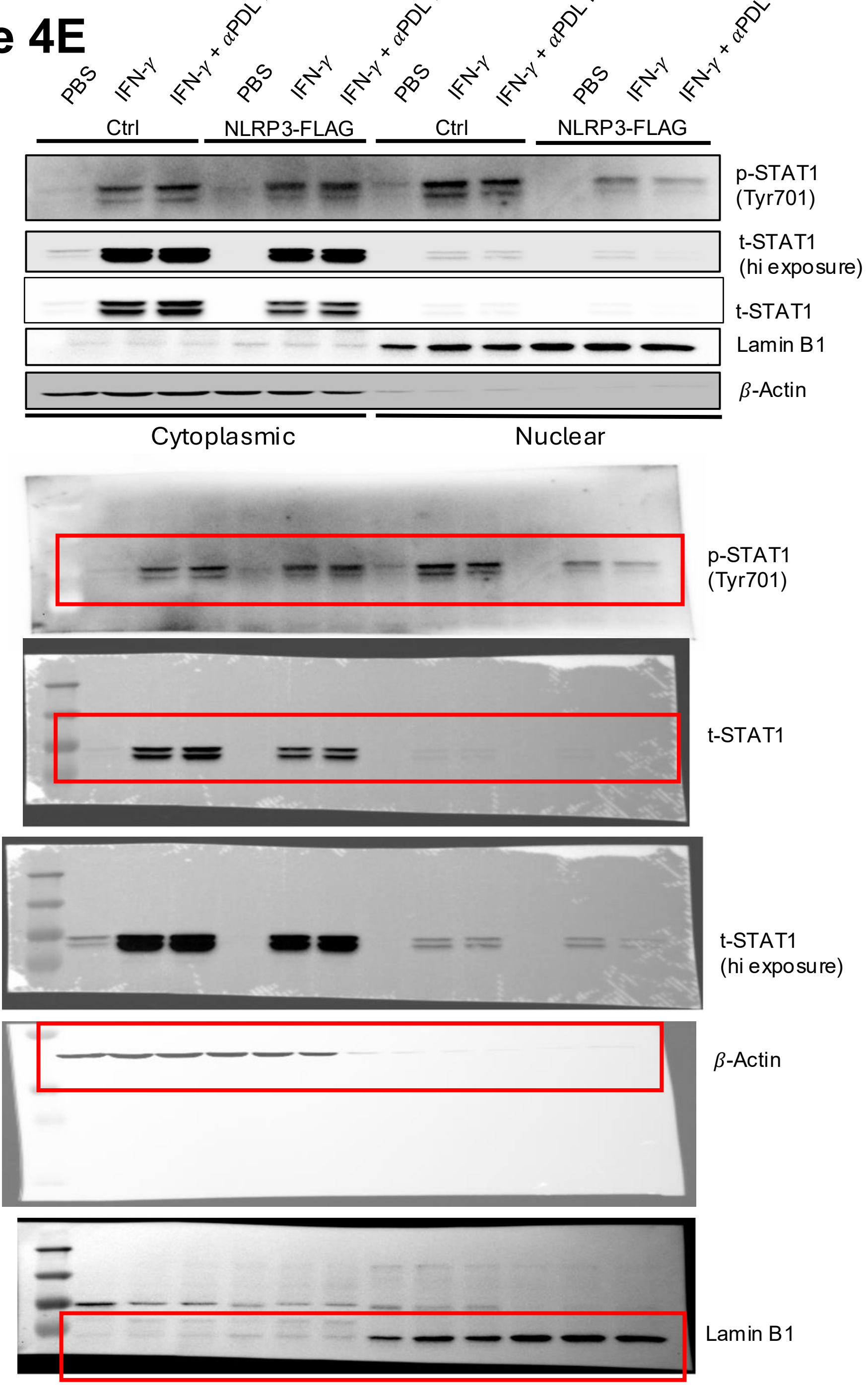

Figure 4F

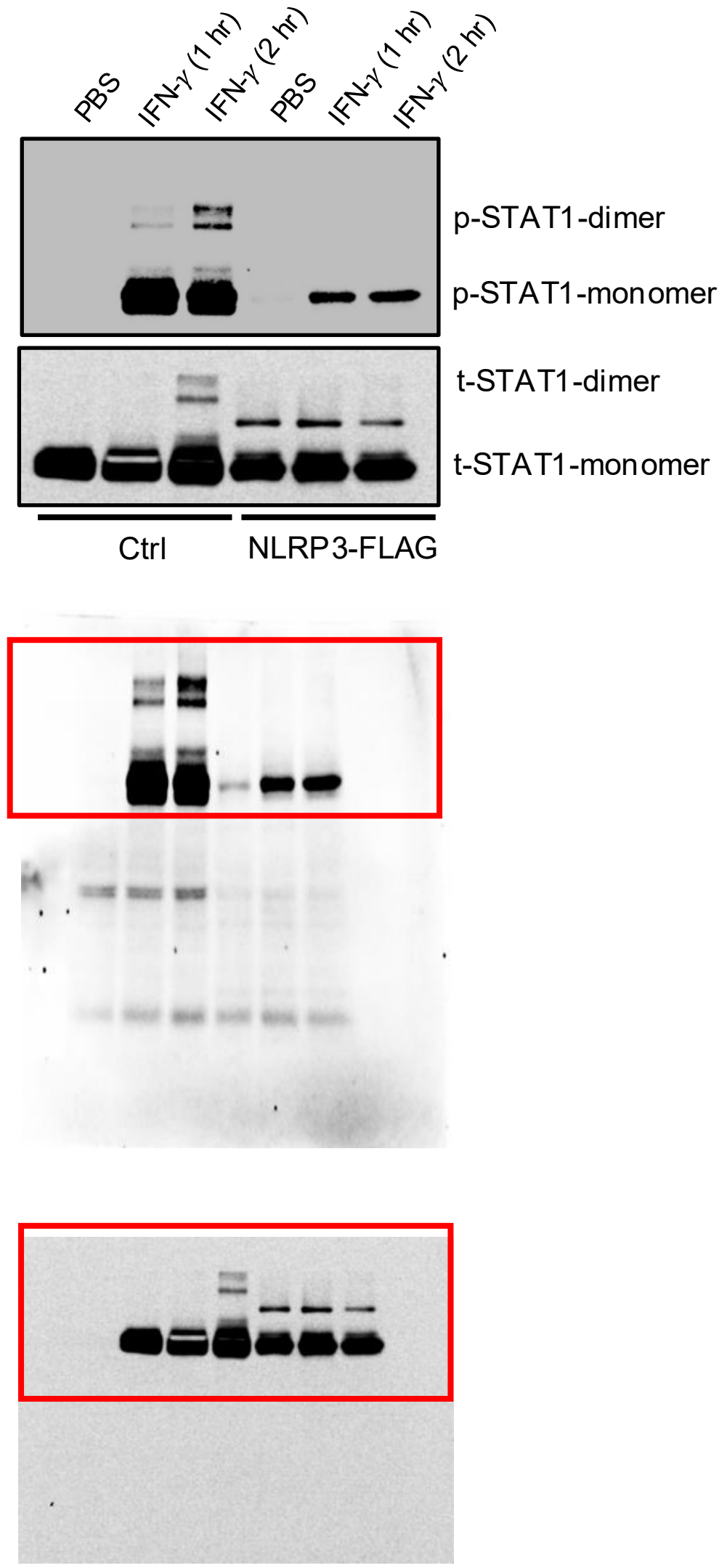

Figure 5A.

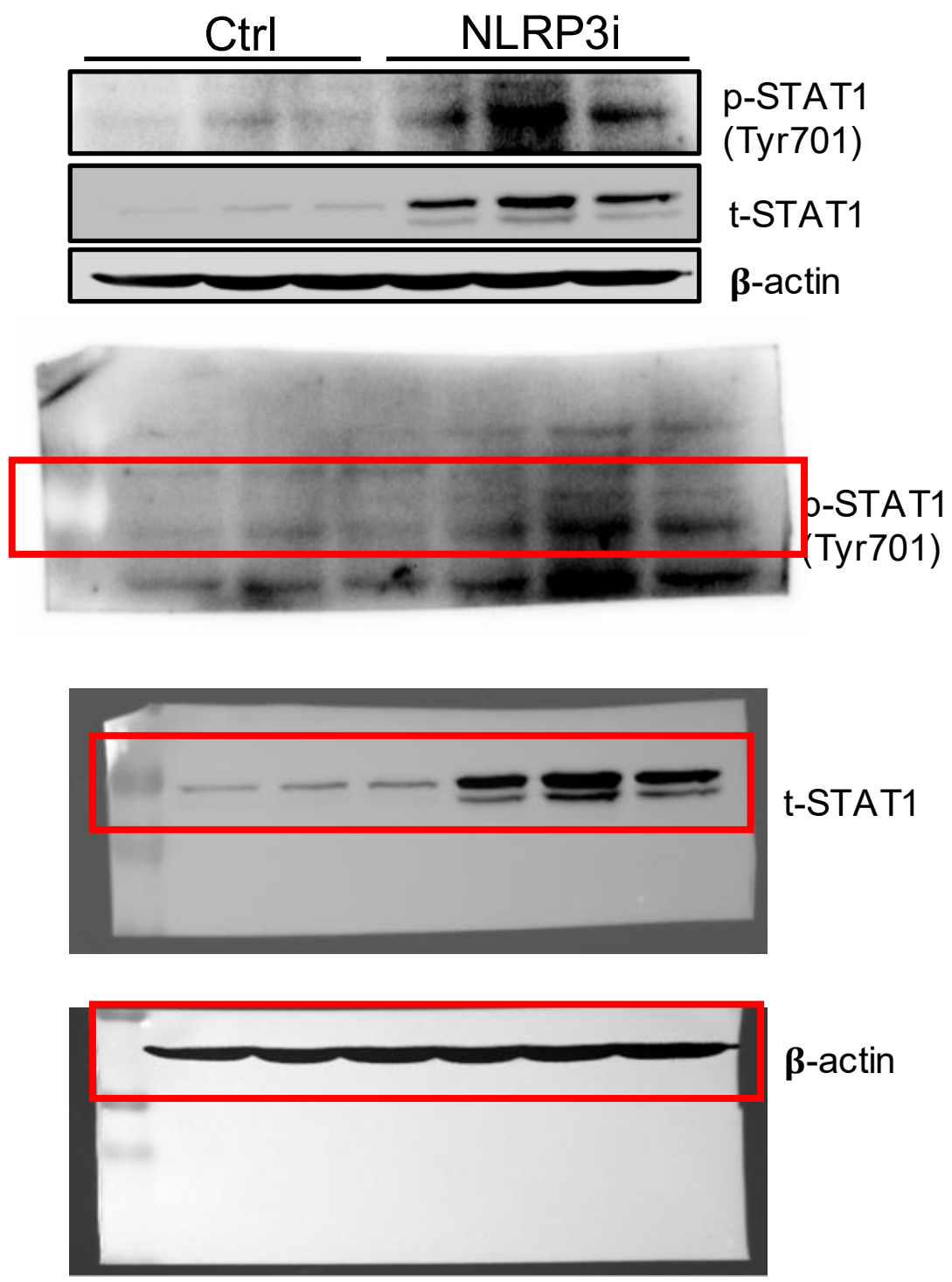

Ctrl
NLRP3i
p-STAT1
(Tyr701)
t-STAT1
β-actin
p-STAT1
(Tyr701)
t-STAT1
β-actin

Extended Data Fig. 1A

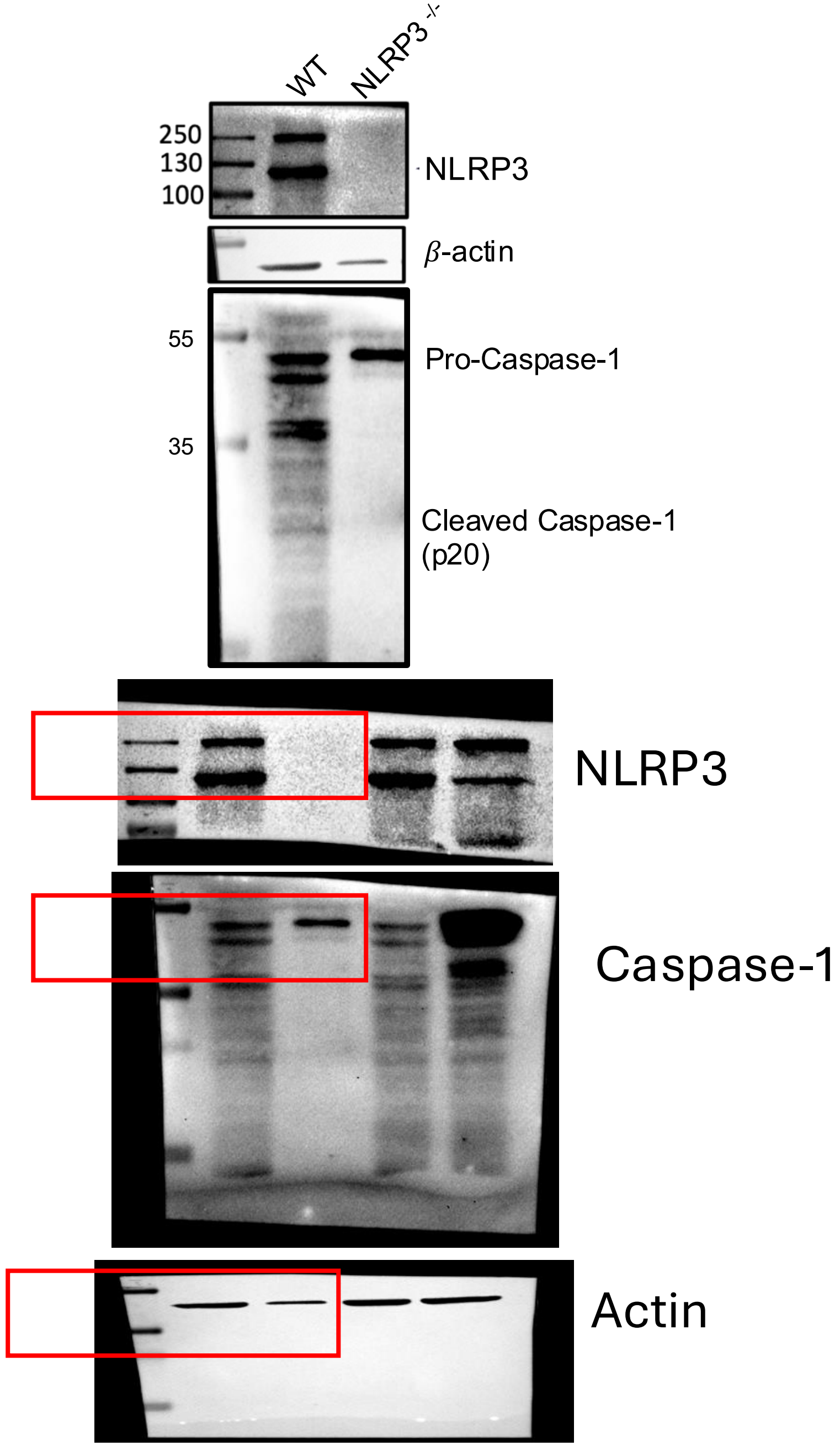

Extended Data Fig. 2A

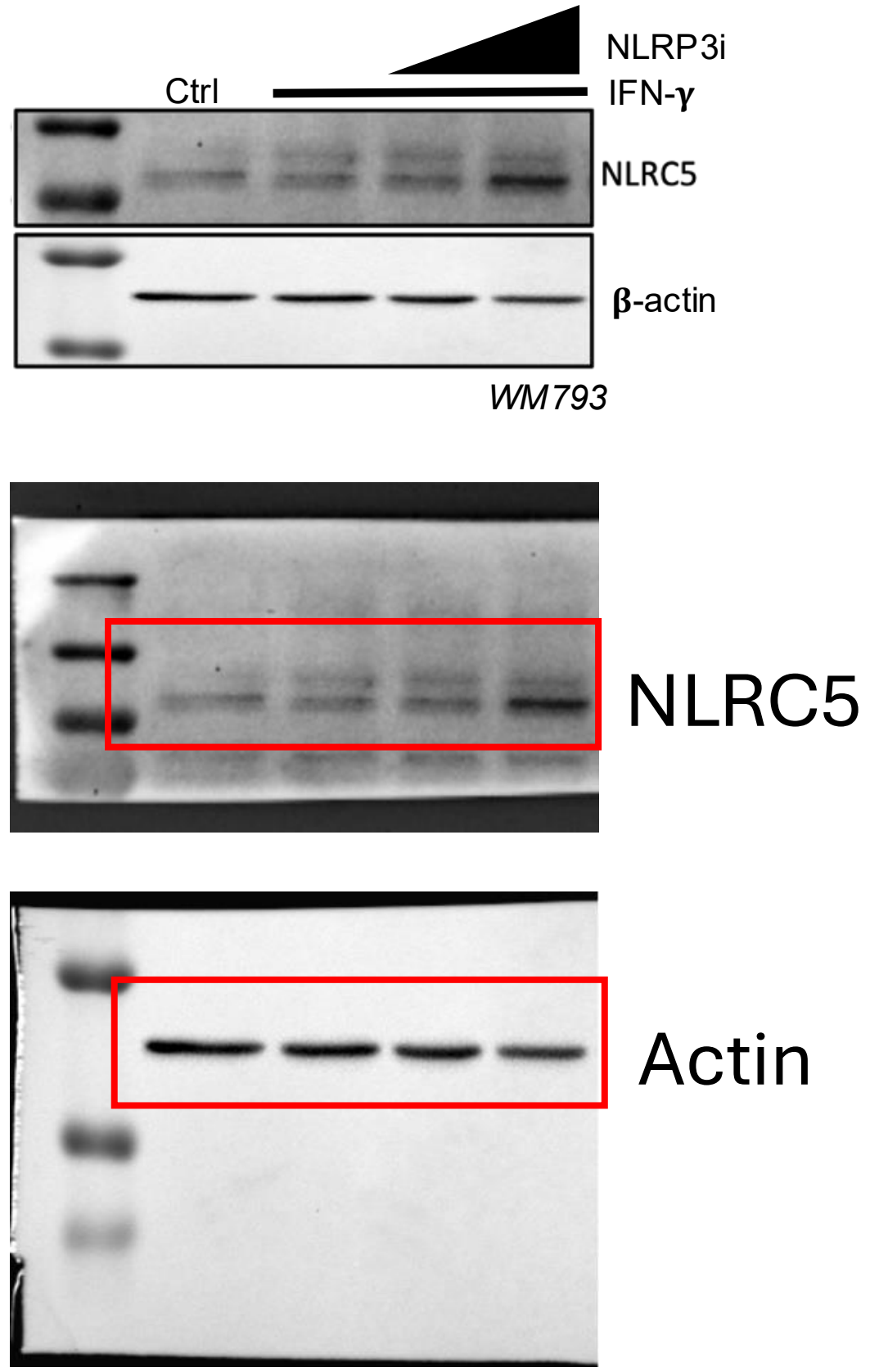

Extended Data Fig. 2B

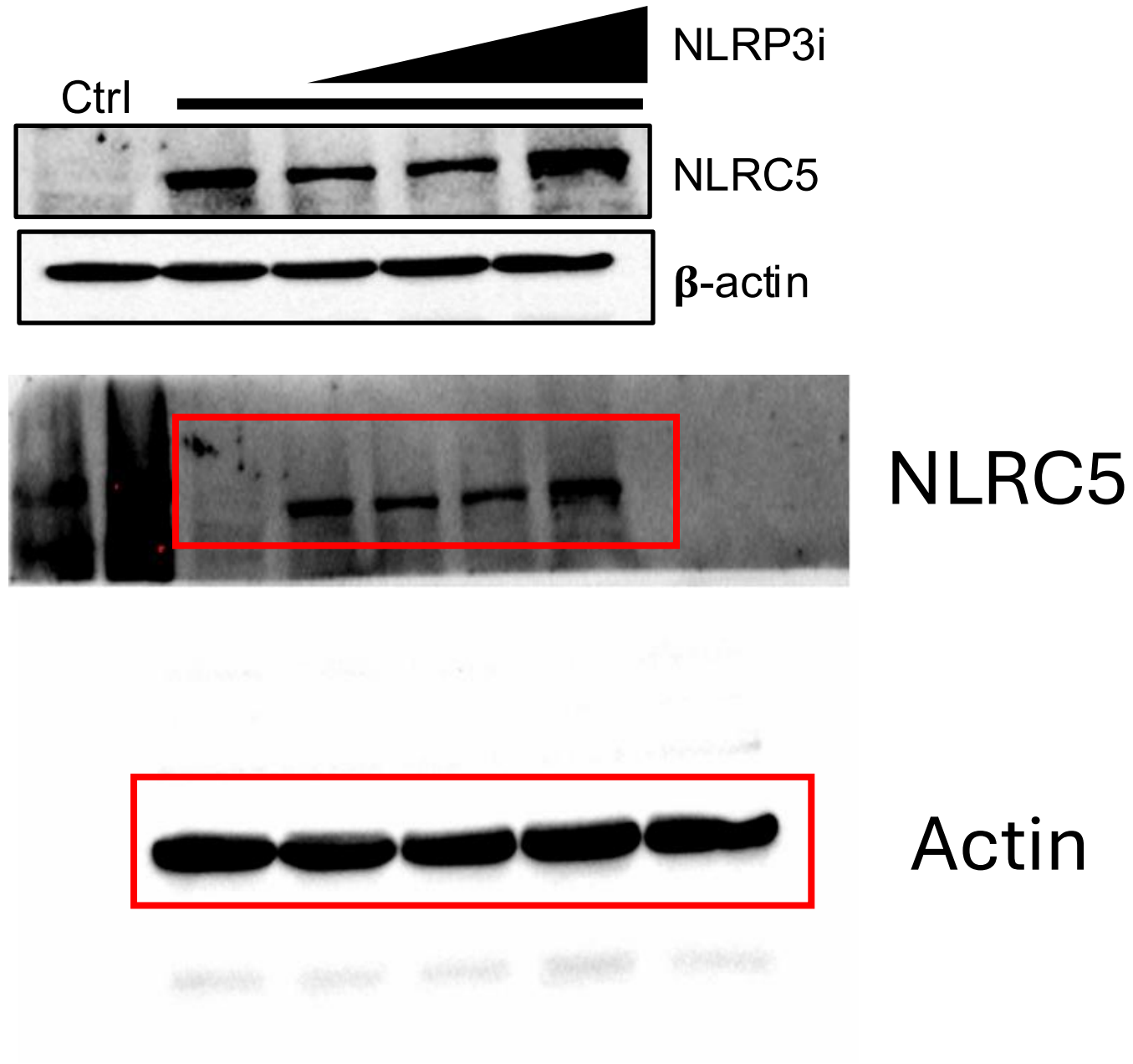

Extended Data Fig. 2E

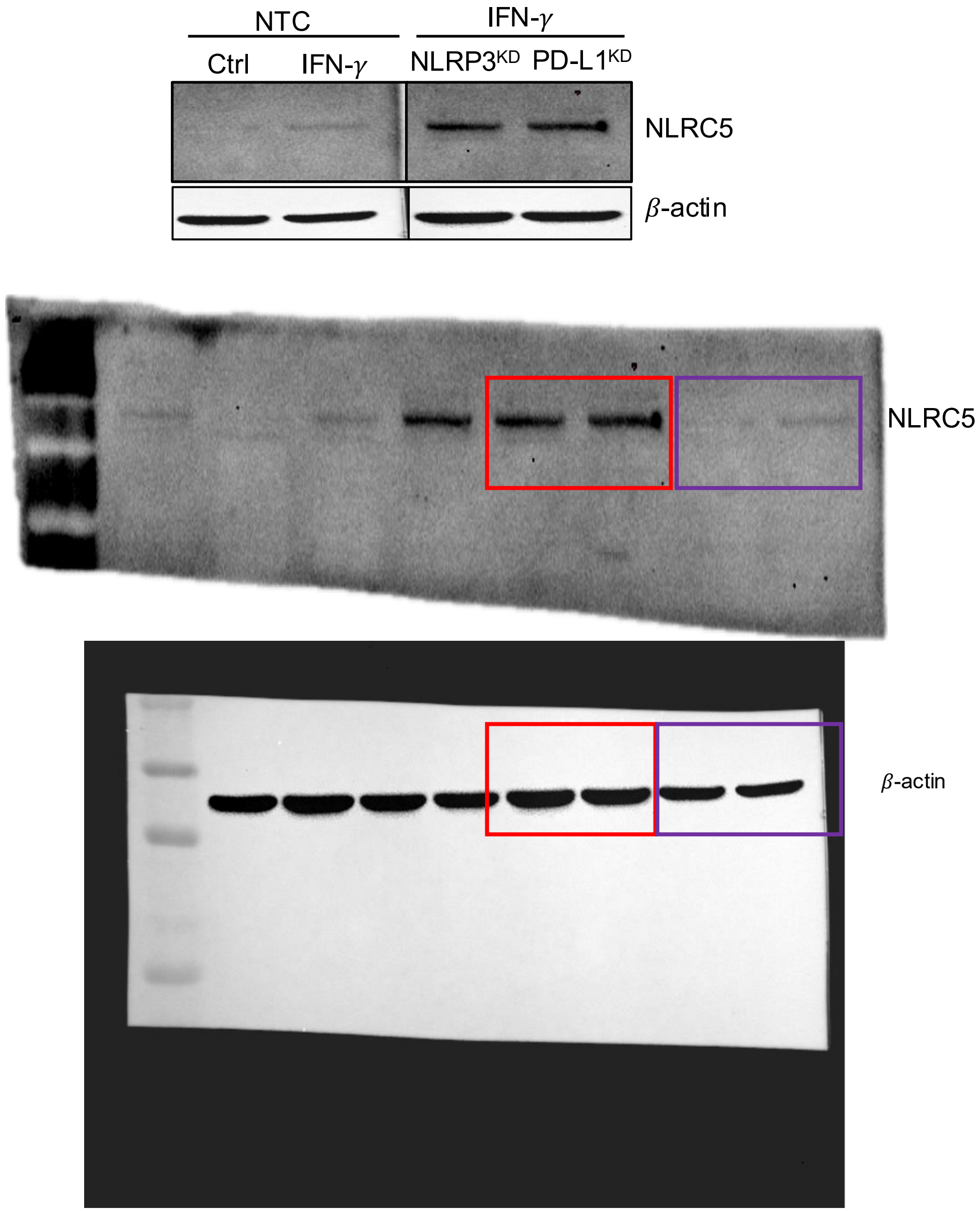

Extended Data Fig. 3A

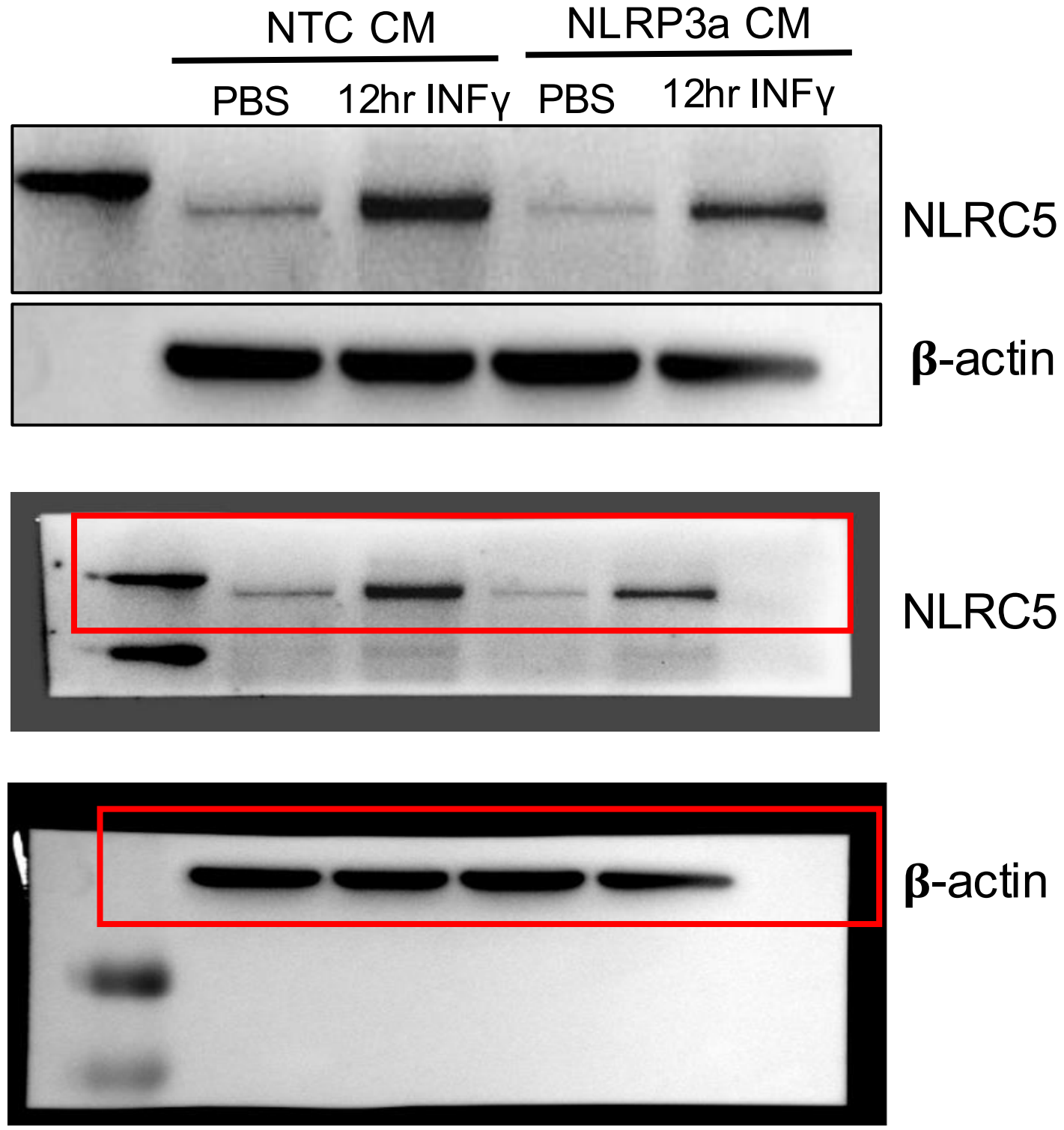

Extended Data Fig. 3C

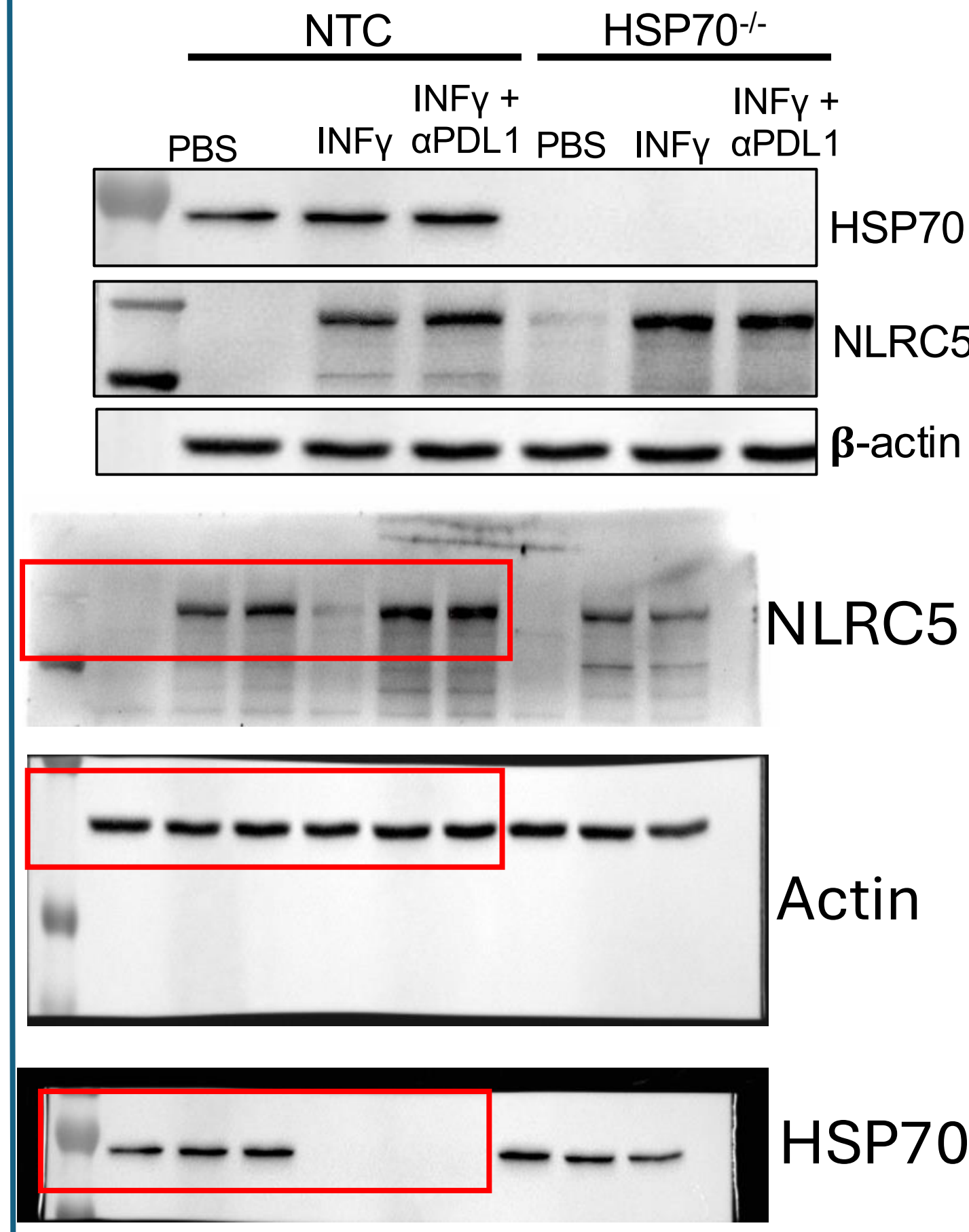

Extended Data Fig. 4B

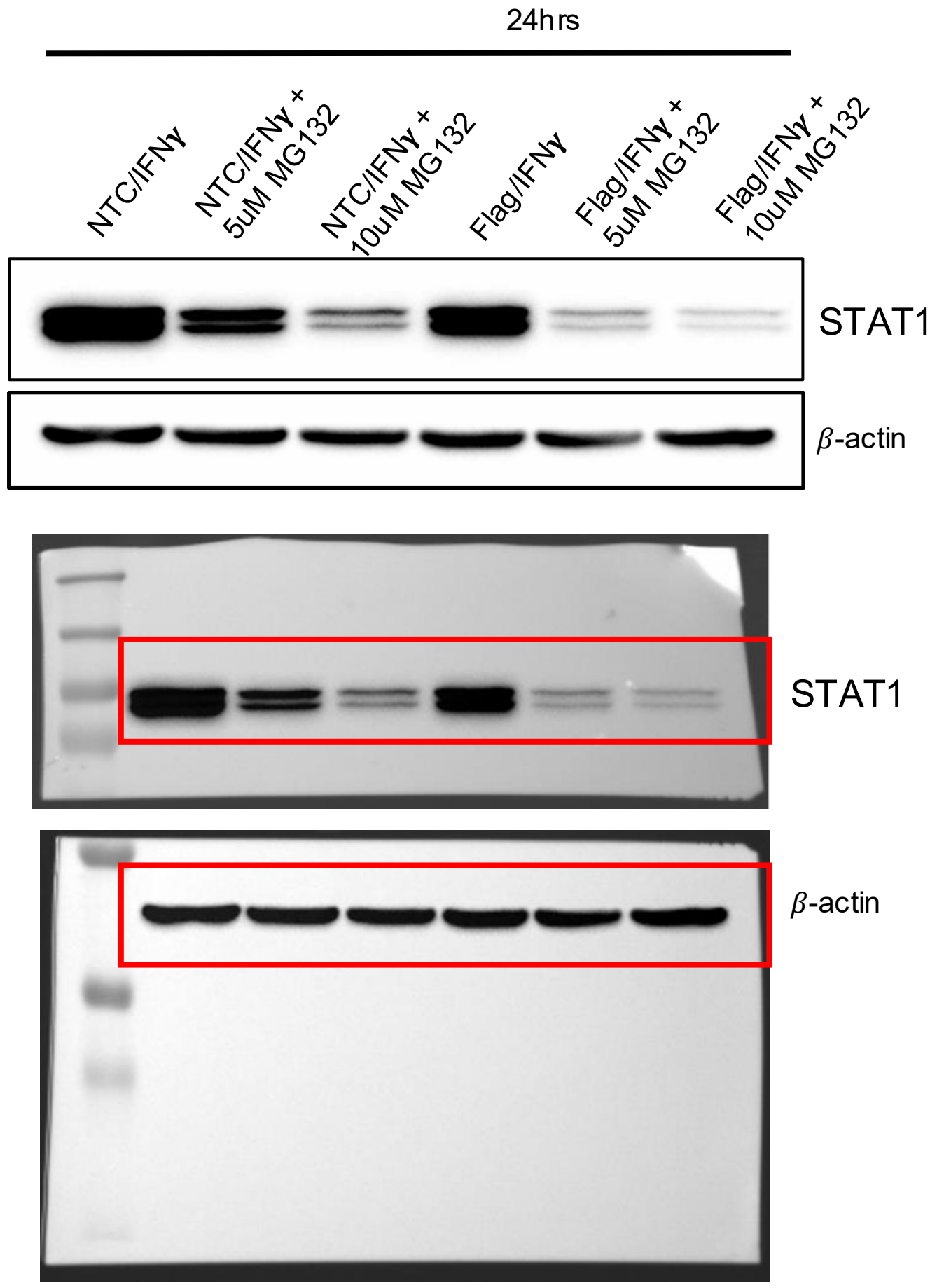

Extended Data Fig. 4C

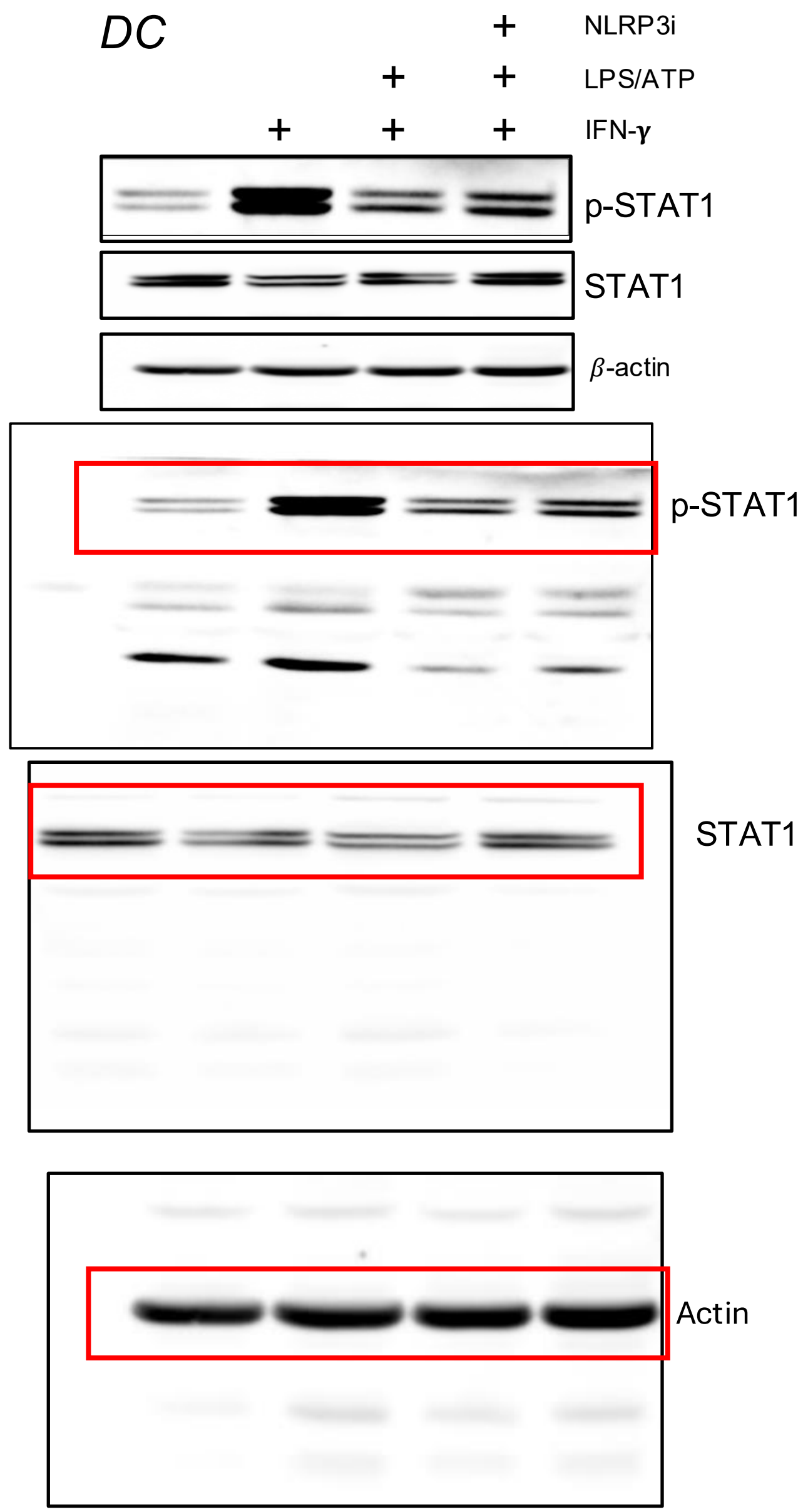

Extended Data Fig. 4F

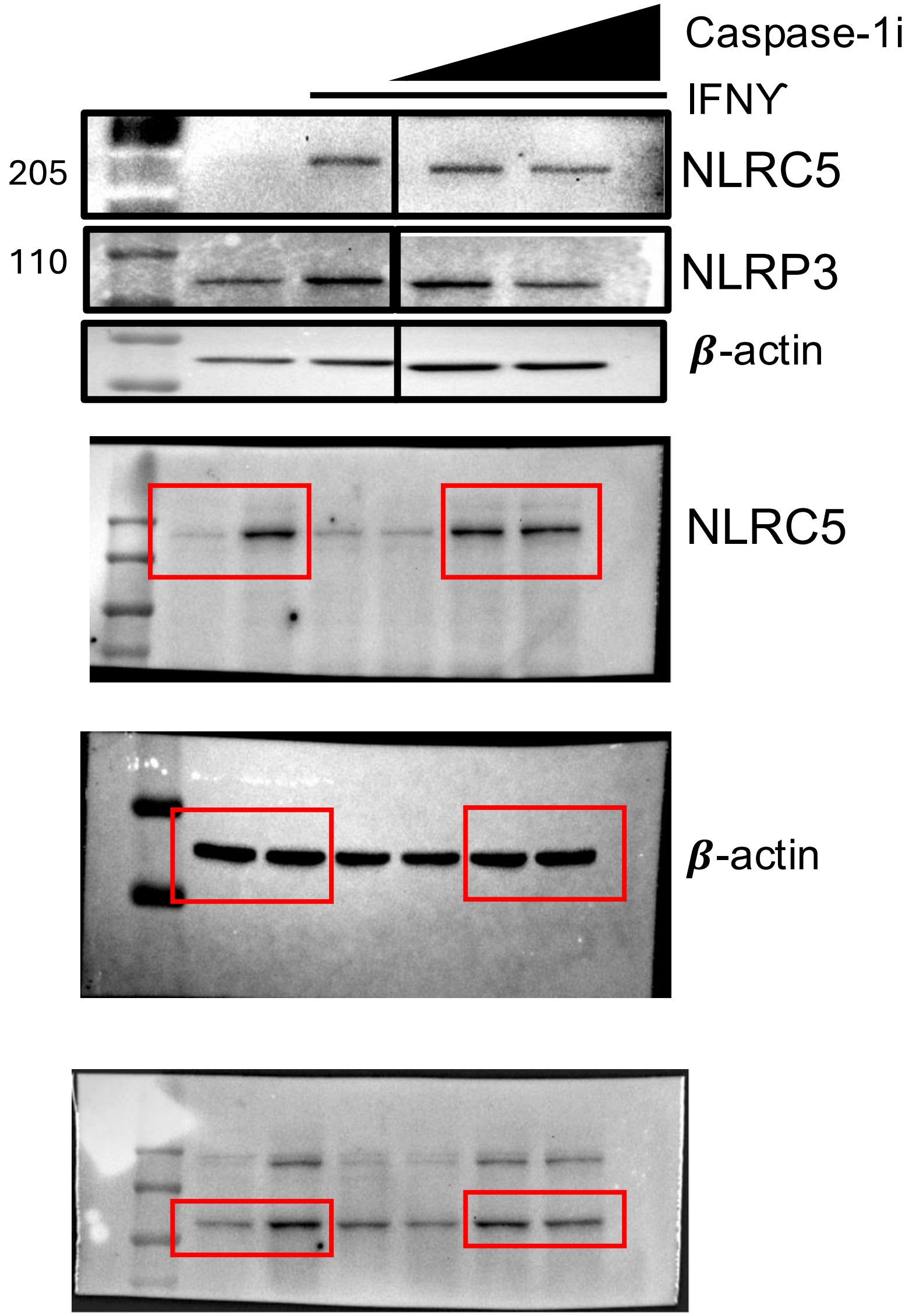

Extended Data Fig. 5B

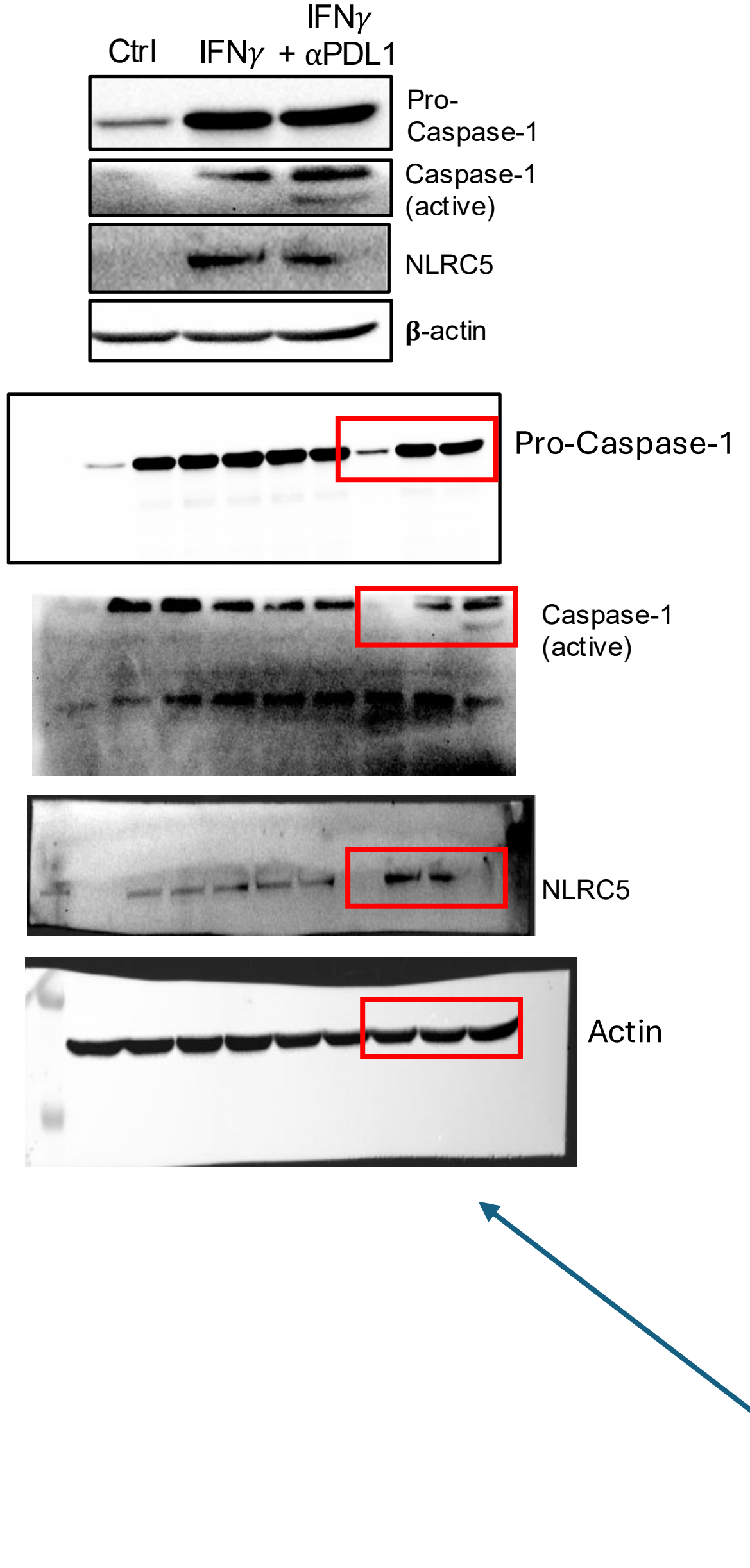

Extended Data Fig. 6A

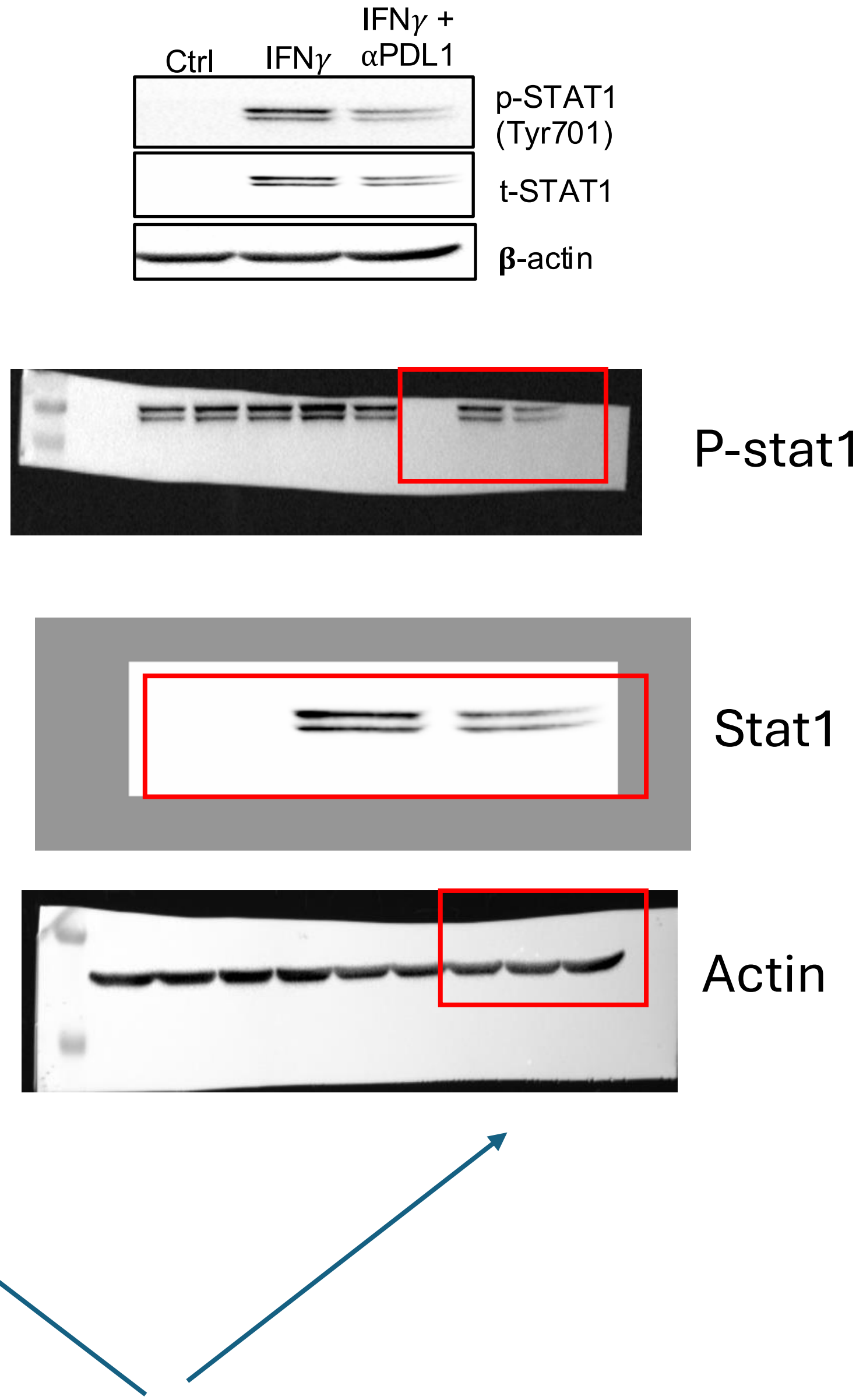

Extended Data Fig. 6B

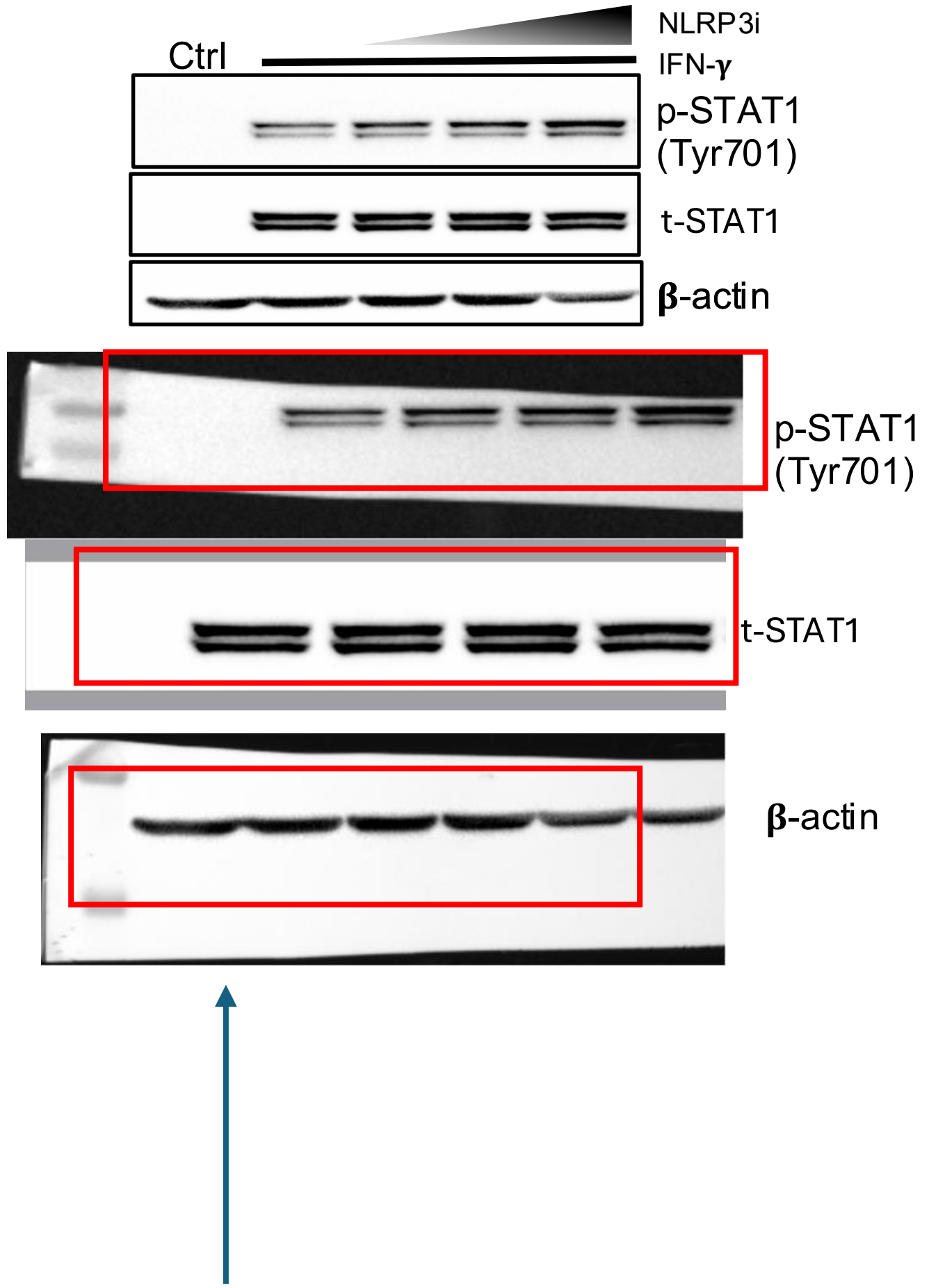

Same GEL/Same $\boldsymbol{\beta}$-actin **(S6B+ S6D)**

Same GEL/Same $\boldsymbol{\beta}$-actin **(S5B+ S6A)**

Extended Data Fig. 6C

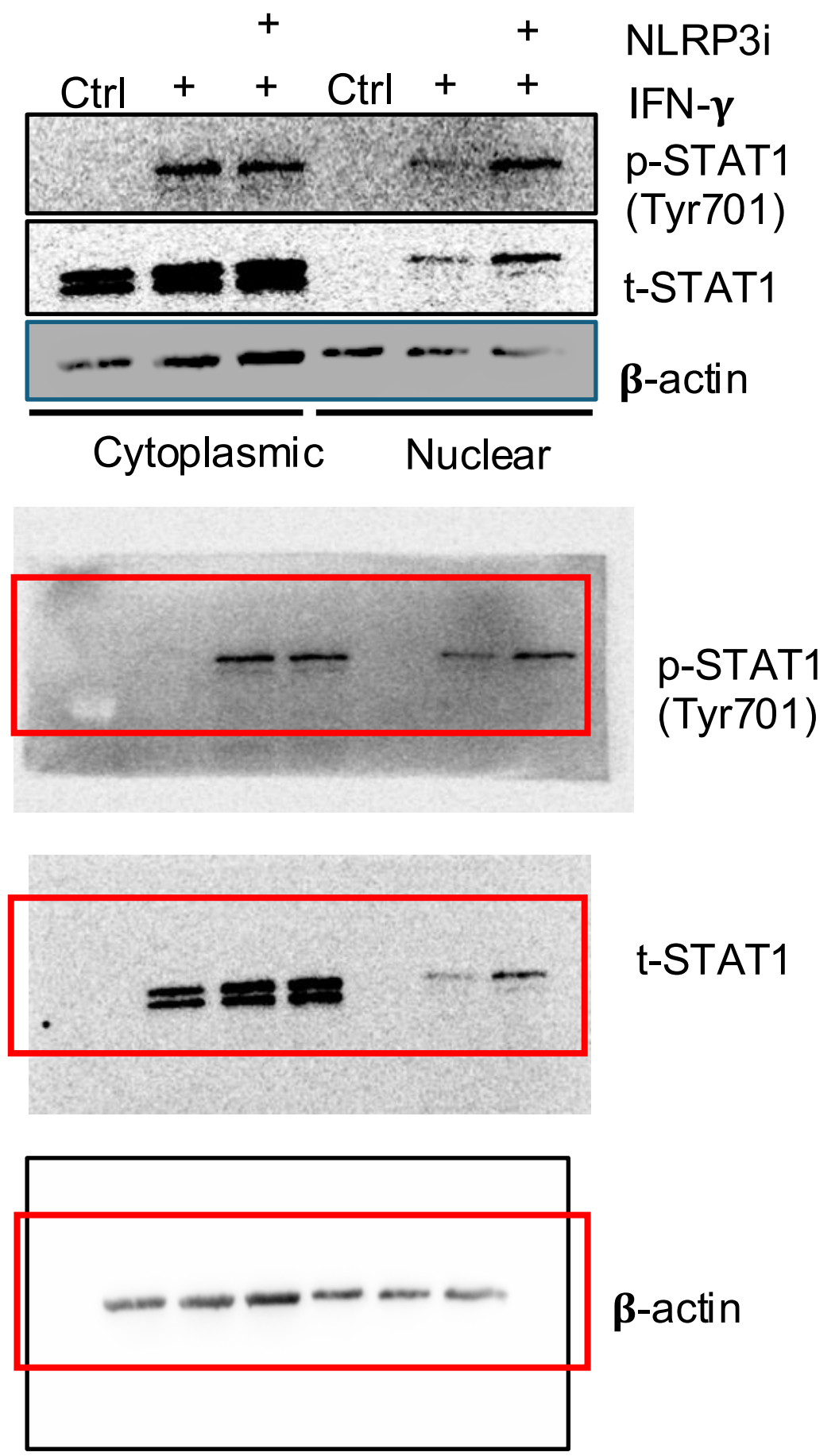

Extended Data Fig. 6D

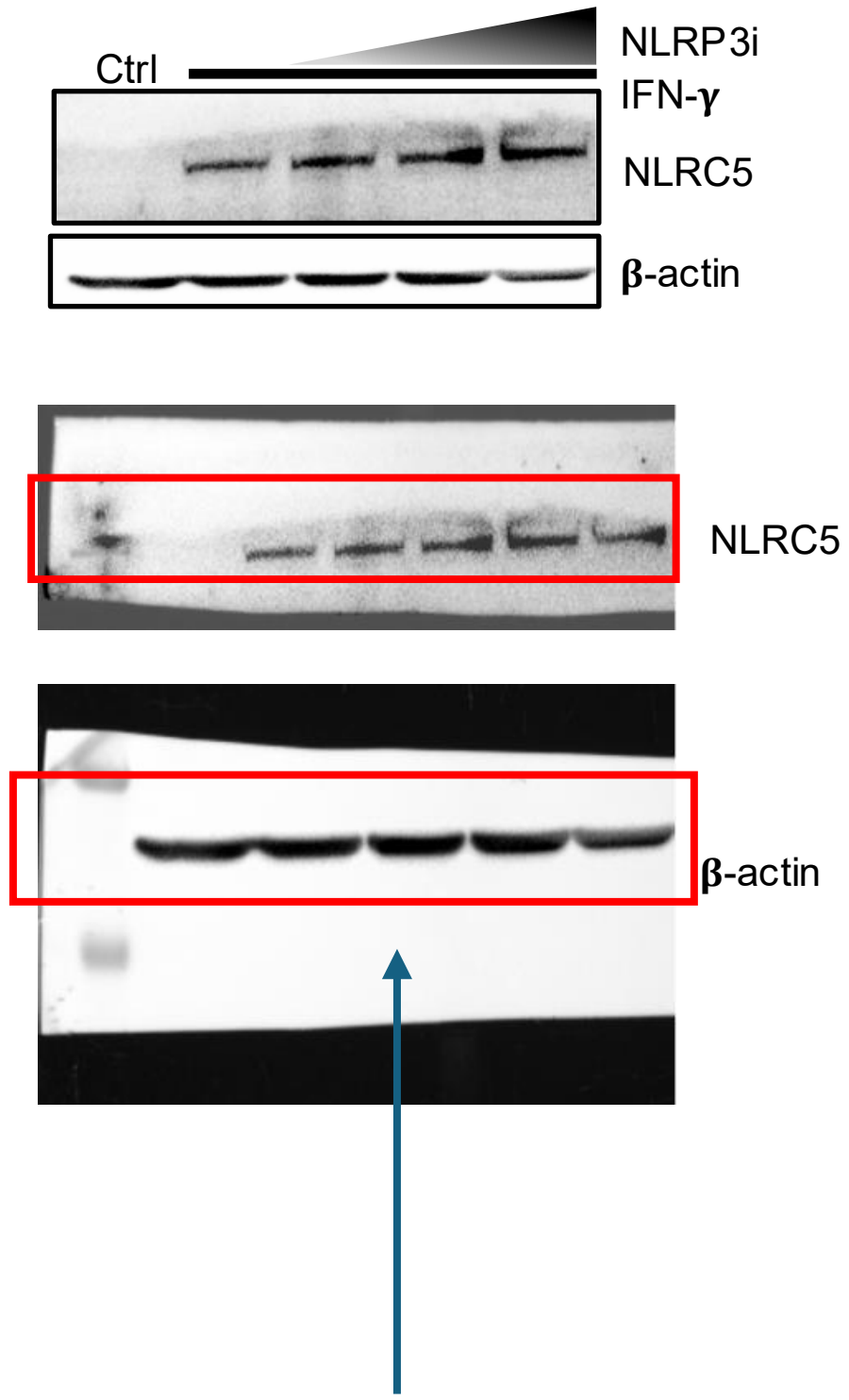

Same GEL/Same
β-actin **(S6B+ S6D)**

Extended Data Fig. 6K

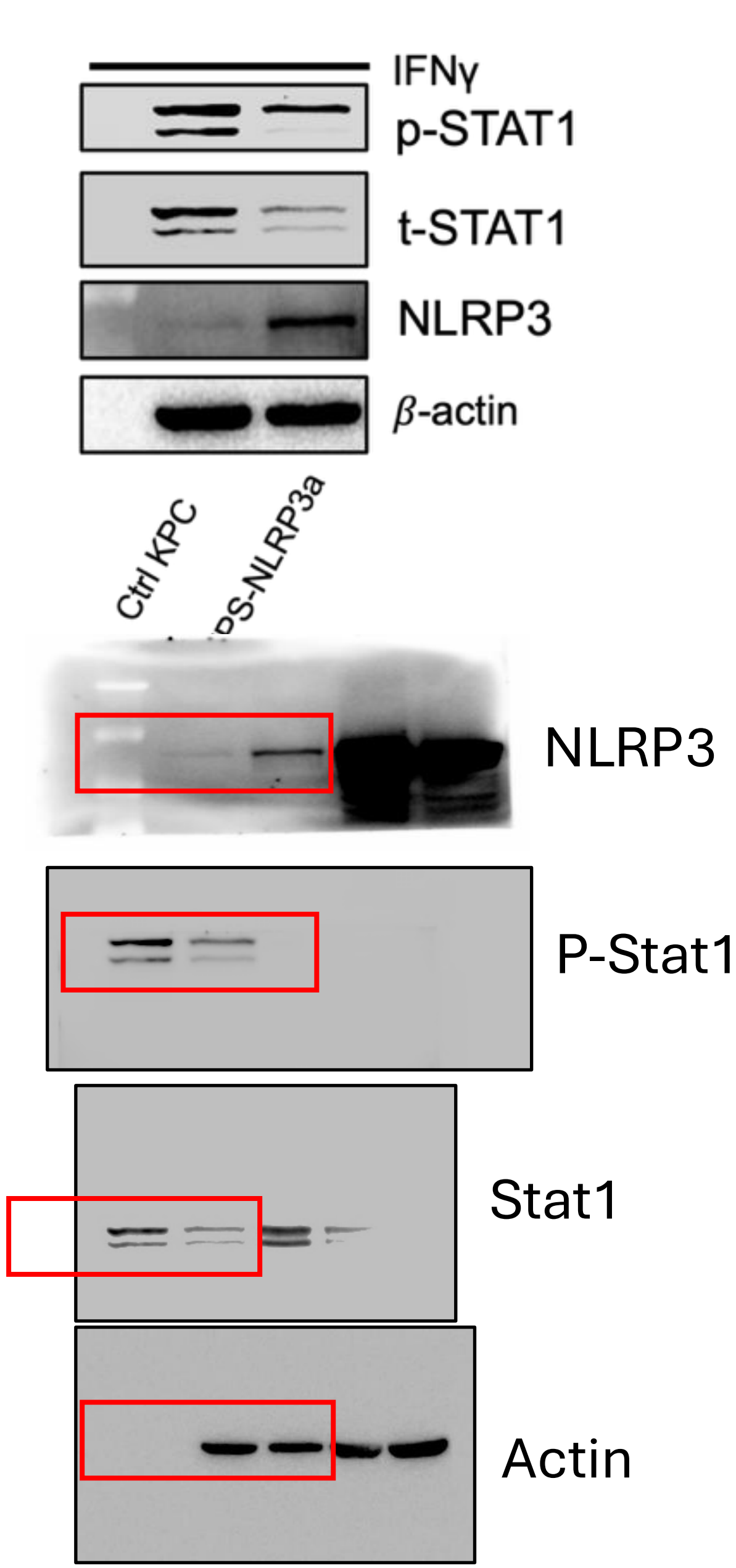

Supplement: Supplementary file 2 [file jci-136-201816-s134.pdf]
